# Optimization of meropenem continuous infusion based on Monte Carlo simulation integrating with degradation study

**DOI:** 10.1371/journal.pone.0313764

**Published:** 2024-12-23

**Authors:** Nguyen Tran Nam Tien, Vu Ngan Binh, Pham Thi Thanh Ha, Dang Thi Ngoc Lan, Yong-Soon Cho, Nguyen Phuoc Long, Jae-Gook Shin, Nguyen Hoang Anh, Truong Anh Quan, Do Ngoc Tuan, Nguyen Khac Tiep, Pham The Thach, Nguyen Hoang Anh, Vu Dinh Hoa

**Affiliations:** 1 National Drug Information and Adverse Drug Reactions Monitoring Centre, Hanoi University of Pharmacy, Hanoi, Vietnam; 2 Department of Analytical Chemistry and Drug Quality Control, Hanoi University of Pharmacy, Hanoi, Vietnam; 3 Department of Pharmacology and PharmacoGenomics Research Center, Inje University College of Medicine, Busan, Republic of Korea; 4 Department of Clinical Pharmacology, Inje University Busan Paik Hospital, Busan, Republic of Korea; 5 Department of Computing, Goldsmiths, University of London, London, United Kingdom; 6 Department of Pharmaceutical BioTechnology, Hanoi University of Pharmacy, Hanoi, Vietnam; 7 Center for Intensive Care Medicine, Bach Mai Hospital, Hanoi, Vietnam; 8 Clinical Pharmacy and Drug Information Unit, Department of Pharmacy, Bach Mai Hospital, Hanoi, Vietnam; Shiraz University of Medical Sciences, ISLAMIC REPUBLIC OF IRAN

## Abstract

**Objective:**

Meropenem degradation poses a challenge to continuous infusion (CI) implementation. However, data about the impact of degradation on the probability of target attainment (PTA) of meropenem has been limited. This study evaluated the stability of meropenem brands and the consequence of in-bottle degradation on PTA in different environmental scenarios.

**Method:**

Seven meropenem generic brands prepared at concentrations of 1 g/48mL and 2 g/48mL in saline were examined at 25, 30, and 37°C over 8 h. A linear mixed-effects model was used to estimate degradation rate constant and potential covariates. In-bottle stability data was subsequently integrated as input for a deterministic and stochastic simulation using a published population pharmacokinetic model of critical illness. The impact of the degradation on target attainment at 98%*f*T>MIC was assessed.

**Results:**

Time, temperature, and infusion concentration were factors affecting the stability of the meropenem solution for all products. The differences in the degradation of seven generics were subtle, so their simulated plasma concentrations were equal. Meropenem CI with 8 h renewal infusion achieved a higher PTA than the extended 3 h infusion, even at the highest degradation condition. The impact of meropenem degradation on PTA was minimal vis-à-vis the meropenem dose, patient’s renal function, and microbial susceptibility. Meropenem degradation reduced PTA by an observable magnitude in patients with augmented renal clearance and difficult-to-treat pathogens. Dose escalation up to 1.5–2g every 8 h could restore this reduction to the target 90% PTA.

**Conclusion:**

Meropenem CI with 8 h of renewal infusion, considering stability even in tropical areas, was feasible to maximize the efficacy to difficult-to-treat pathogens.

## Introduction

Meropenem is a crucial antibiotic treating life-threatening infections caused by Gram-negative bacteria. The emergence of reduced susceptible pathogens with a substantially high minimum inhibitory concentration (MIC) of meropenem hinders the effectiveness of this backbone antibiotic in a highly resistant environment such as the Intensive Care Unit (ICU) setting and the tropical area [1, 2]. Furthermore, alterations in meropenem pharmacokinetics in critically ill patients were reported due to changes in pathophysiological characteristics and the involvement of critical care interventions [1, 3]. Therefore, the sub- or supra-therapeutic concentration of meropenem might reduce its efficacy or put the patient at risk of toxicity [4]. Extending the infusion time of meropenem up to 3 h has been recommended to maximize the duration of time that the free drug concentration remains above the MIC during a dosing interval (*f*T>MIC), which is the proven Pharmacokinetics/Pharmacodynamics (PK/PD) index for meropenem [1, 5, 6]. Continuous infusion (CI) showed a further improvement in the probability of target attainment (PTA) in elevated MIC isolates when the target of 100%*f*T>MIC was applied [7–10]. Moreover, aggressive beta-lactams PK/PD target (i.e., 100%*f*T>4xMIC) demonstrated clinical benefits in critical illness [11]. However, the concern about the stability of meropenem in the prepared solution might prevent the implementation of CI, especially at the high temperatures of tropical environments. The reduction in concentration due to degradation might decrease microbiological activity and increase the risk of antimicrobial resistance emergence.

Previous studies on the physicochemical properties of meropenem demonstrated that this drug was unstable in aqueous solution due to hydrolysis [12]. Therefore, the length of meropenem prolonged infusion might be questioned, as meropenem commences to decay upon dissolution [13]. *In vitro* studies showed that degradation of meropenem depended mainly on temperature and infusion concentration and determined the appropriate length of infusion of 6 to 12 h based on a criterion of 90% remaining concentration compared to the original concentration [12, 14–18]. A study integrated the degradation equation with a population pharmacokinetics (popPK) model to show the simulated PK profile [19]. However, evidence about the impact of meropenem’s degradation on PTA has been limited using appropriate stochastic simulation. One up-to-date study reported that meropenem degradation affected plasma concentrations and bacterial load and highlighted that 8 h bottle renewal improved PK/PD target attainment [20]. Furthermore, questions about the stability of certain generic brands versus original/innovative brands have been raised [17, 18, 21]. Those approved in Europe have shown instability compared to the originator [18], and some others have also revealed therapeutic nonequivalence [22]. Different meropenem brands might have different plasma concentration profiles due to the degradation process. Therefore, the degradation process should be considered when choosing appropriate prolonged infusion modes (extended infusion (EI) or CI) and meropenem brands in clinical settings.

To provide information on the stability of different meropenem brands and address the concern about meropenem CI implementation, this study aimed to assess the *in vitro* degradation rate of meropenem in different generic products and its consequence on the PTA of clinical illness.

## Materials and methods

### Chemicals and reagents

Meropenem standard (certified reference material) was purchased from Sigma-Aldrich (Lot. LRAB7853, content 71.0%). Phosphoric acid and methanol were obtained from Merck (Darmstadt, Germany). Sodium chloride 0.9% intravenous infusion acquired from B-Braun (Vietnam, Lot. 204017741) was used as a solvent for meropenem solutions to mimic clinical administration. The in-house double-distilled water was freshly prepared.

Seven meropenem products were used for this study, including Meropenem Anfarm 1 g (Anfarm Hellas S.A, Greece, Lot. 20G159), Emerop 0.5 g (Euvipharm Joint Stock Company, Vietnam, Lot. 23148002), Pimenem 500 mg (Pymepharco Joint Stock Company, Vietnam, Lot. 010219), Q–PEM Inj. 1 g (Jiel Pharmaceutical Co.; Ltd., Korea, Lot. NEV701), Merovia (Remedina S.A., Greece, Lot. 20104), Meropenem 1 g (VCP Pharmaceutical Joint Stock Company, Vietnam, Lot. 630718), and Maxpenem Injection 1 g (JW Pharmaceutical Corporation, Korea, Lot. 18003). These brand names were codified as A, E, I, Q, R, V, and X, respectively.

### Stability studies

#### Investigated temperature, infusion concentration, and time

The selected concentrations of 1 g/48mL and 2 g/48mL corresponding to the dosage concentration of meropenem for common infection and severe infection in ICU were examined, respectively [12, 18]. Temperatures at 25°C and 37°C were commonly used to test meropenem infusion solution stability [12]. The temperature at 30°C was selected to present the room temperature in the tropical areas [23]. The tested time of up to 8 h was chosen because it is considered long enough for clinical practice [24].

Meropenem concentrations were measured via a validated High-Performance Liquid Chromatography (HPLC) method [25]. Briefly, 0.13 g and 0.26 g of the test powder (equivalent to 0.1 g and 0.2 g of meropenem) were accurately weighed into a 5 mL volumetric flask and dissolved with 0.9% sodium chloride to achieve the test solution with meropenem concentrations of 1 g/48mL and 2 g/48mL, respectively. The 0 h solution was prepared by immediately withdrawing a proper amount of the test solution and diluted with water to obtain a meropenem concentration of approximately 80 μg/mL. The remaining solutions were covered with aluminum foil and incubated at 25°C, 30°C, and 37°C. After 1, 2, 3, 4, 5, 6, 7, and 8 h, a certain amount of the solution was withdrawn and diluted with water in the same dilution factor as the 0 h solution. All samples were filtered through 0.45 μm cellulose. An injection volume of 50 μL was operated by an Agilent 1200 chromatography system consisting of column Inertsustain C8 (4.6 x 250 mm, 5μm) and a C8 guard column; mobile phase of 0.1% phosphoric acid: methanol (75:25); eluent rate of 1.4 mL/min for 10 min with UV detector monitoring at 310 nm. The method showed accuracy (bias of 0.2%– 0.6% at concentrations of 48, 64, and 80 μg/mL) and precision (0.53% and 0.85% for intraday and interday at a concentration of 80 μg/mL, respectively). The linearity range was 32 to 96 μg/mL. Details of method validation are shown in the S1 File. The concentration of meropenem in the tested samples was calculated based on the peak area of meropenem and the calibration curve prepared on the same day. The assay result of meropenem at various storage times (1 to 8 h) was calculated as a percentage of the initial concentration (0 h).

#### Model stability data

The decomposition of meropenem has been widely described using first-order kinetic (1) [26]. To investigate the impact of time, temperature, infusion concentration, and brand on meropenem degradation, a linear mixed-effects model was applied using the lmerTest package version 3.1–3 in R 4.2.3 [27]. These factors were tested as significant for degradation rate (k_deg_) (1) [16, 18, 26].

$$log\left(\frac{c}{c_{0}}\right)=\mu +\theta -\left(\beta_{0}+\beta_{1}*T+\beta_{2}*C_{infu}+\beta_{3}*B+\eta \right)*t+\varepsilon$$ (1)

where C is the concentration (mg/L) at time t (h), C_0_ is the initial concentration (mg/L), k_deg_ is the degradation rate constant (h^-1^), T is the temperature (°C), C_infu_ is the infusion concentration (mg/L), and B is the pharmaceutical products evaluated in this study. The random effects, denoted as θ, η, and ε for intercept, slope, and residuals, were assumed to follow the normal distributions with variances of ω_1_^2^, ω_2_^2^, and σ^2^, respectively. To estimate the 95% confidence intervals for the coefficients, a parametric bootstrap method was employed with 5,000 iterations, each using subsamples of size 365. The likelihood ratio test was conducted to select an optimal model. In addition, individual-level stability parameters were obtained as conditional modes using the final model to evaluate the extent of degradation for each generic brand.

### PK/PD simulation studies

#### The published popPK model

Meropenem CI was demonstrated to have better PK/PD targets in critically ill patients and difficult-to-treat pathogens [7–10]. Therefore, the popPK model developed by Ehmann *et al*. focussing on critically ill patients with severe infections and without continuous renal replacement therapy (CRRT) was considered appropriate [7]. The rationale is that the demographics of participants in this study were consistent with ICU patients in general [7]. This study included patients with renal function varying broadly (median creatinine clearance (Clcr) of 70.8 mL/min and 5%– 95% percentiles of 34.8 mL/min– 160 mL/min), which is typical for the critically ill population [7, 28]. A model without incorporating CRRT was also selected to reduce the complexity of the structural component of this popPK model without losing clinical interpretation. Moreover, the model used the rich sampling schedule (N_patients_ = 48, N_samples_ = 1376) [7] and was externally validated by Yang *et al*. to ensure the validity of the model in patients outside the study and the robustness of the simulation result [29].

#### Simulation procedure

**Fig 1:**
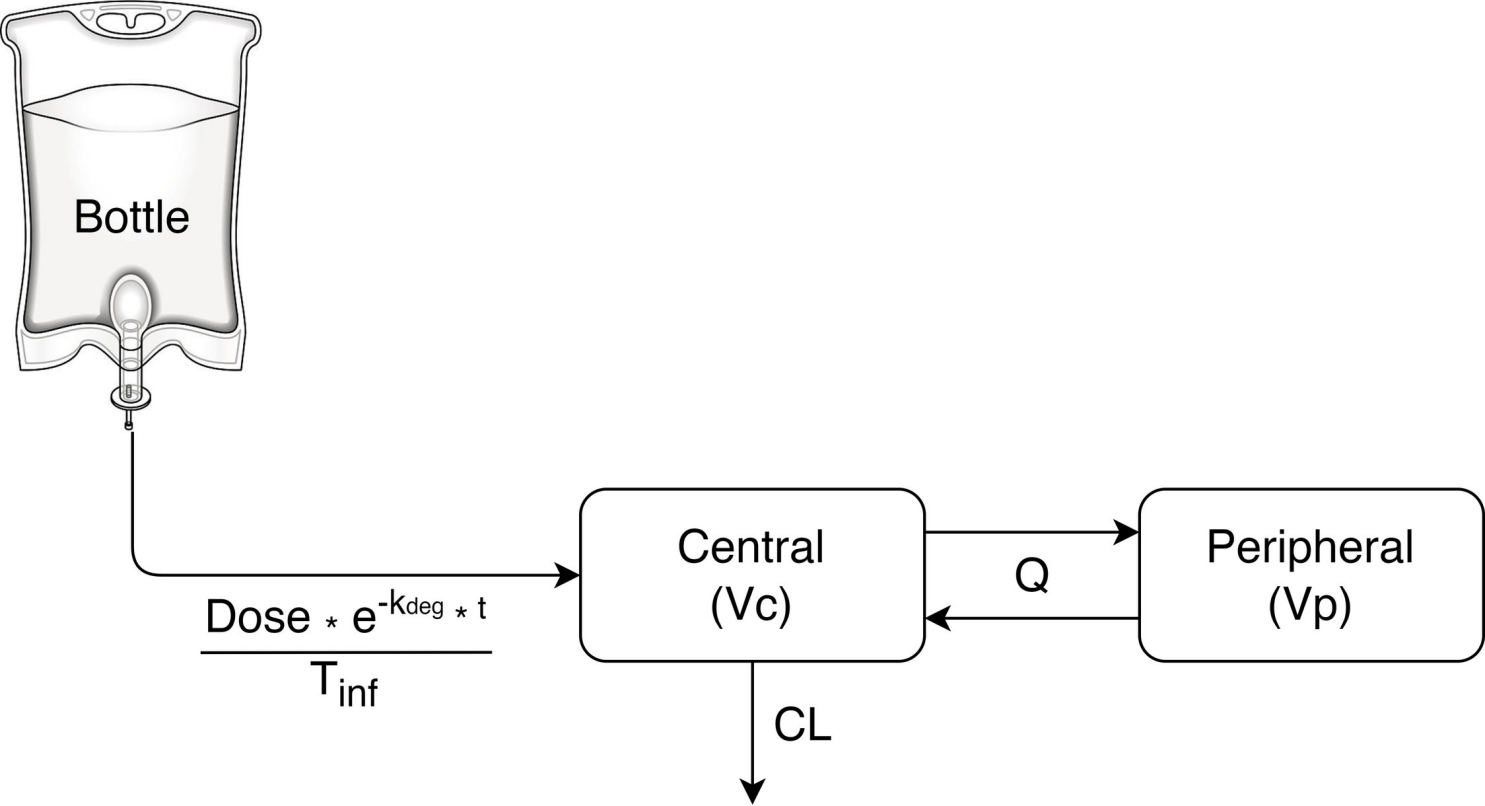
Schematic illustrating the integration of the in-bottle degradation and the two-compartment population pharmacokinetic model. The drug input from the bottle was simulated with different infusion dose regimens altered by degradation. The drug distribution and elimination followed the published pharmacokinetic model [7]. Dose, k_deg_, t, T_inf_, Vc, Vp, Cl, Q denoted prescribed dose (mg), degradation rate constant (h^-1^), time (h), length of infusion (h), central’s volume of distribution (L), peripheral’s volume of distribution (L), total clearance (L/h) and inter-compartmental clearance (L/h), respectively.

In order to explore the impact of in-bottle degradation on the meropenem pharmacokinetic profile and target attainment, the estimated degradation rate constant (k_deg_) from the *in vitro* stability study was integrated into the two-compartment popPK model during the infusion phase (Fig 1) as follows:

$$\frac{dA_{central}}{dt}=\frac{Dose*e^{-k_{deg}*t}}{T_{inf}}-A_{central}*\left(\frac{Cl}{Vc}+\frac{Q}{Vc}\right)+A_{peripheral}*\frac{Q}{Vp},t\leq T_{inf}$$

$$\frac{dA_{central}}{dt}=-A_{central}*\left(\frac{Cl}{Vc}+\frac{Q}{Vc}\right)+A_{peripheral}*\frac{Q}{Vp},t>T_{inf}$$

$$\frac{dA_{peripheral}}{dt}=A_{central}*\frac{Q}{Vc}-A_{peripheral}*\frac{Q}{Vp}$$

where *A*_*central*_ and *A*_*peripheral*_ are amounts (mg) of meropenem in the central and peripheral compartments with a corresponding volume of distribution (L) are *Vc* and *Vp*; *Dose* is a prescribed dose (mg); t is time (h); *T*_*inf*_ denoted infusion time (h); *Cl* is the total clearance; *Q* is the clearance between the central and peripheral compartments (L/h) and therefore the forward rate from central to peripheral compartment was denoted as *Q/Vc* and the reverse rate was *Q/Vp*; and *k*_*deg*_ is the degradation rate constant (h^-1^) obtained in the experimental study.

For dosage regimens, the 3 h, 4 h, 6 h, and 8 h prolonged infusion of meropenem were tested with a total daily dose of 3, 4.5, and 6 g/day. A loading dose of 500 mg over 30 min was applied for both CI and EI [7, 24]. The PK/PD target of 98%*f*T>MIC was used due to the inadequate protein affinity and the impossibility of achieving 100%*f*T>MIC at the infusion initiation on treatment day 1 (i.e., first 30 min (2% of 24 h) of the growing phase of concentration-time) [7, 24].

For deterministic simulation, a typical patient was applied with a Clcr of 80.8 mL/min, a body weight of 70 kg, and serum albumin of 2.8 g/dL, as described by Ehamann *et al*. [7]. For stochastic simulation, Monte Carlo simulation was performed with 1000 patients per dosage regimen and condition to show how the degradation could impact the PTA results. Random variables included Cl, Vc, and Vp; they were simulated following log-normal distributions with inter-individual variabilities (IIV) of 27.1%, 31.5%, and 16.9%, respectively (S4 File). The inter-occasion variability (IOV) of 12.5% was also included for Cl [7]. Clcr was also simulated using a uniform distribution. The PTA was calculated on the first day of treatment, and a threshold of 90% PTA was considered acceptable [7, 30]. The RxODE 1.1.6 in R 4.1.1 was used to recode the popPK model and perform simulation [31].

## Results

### Stability studies

**Fig 2:**
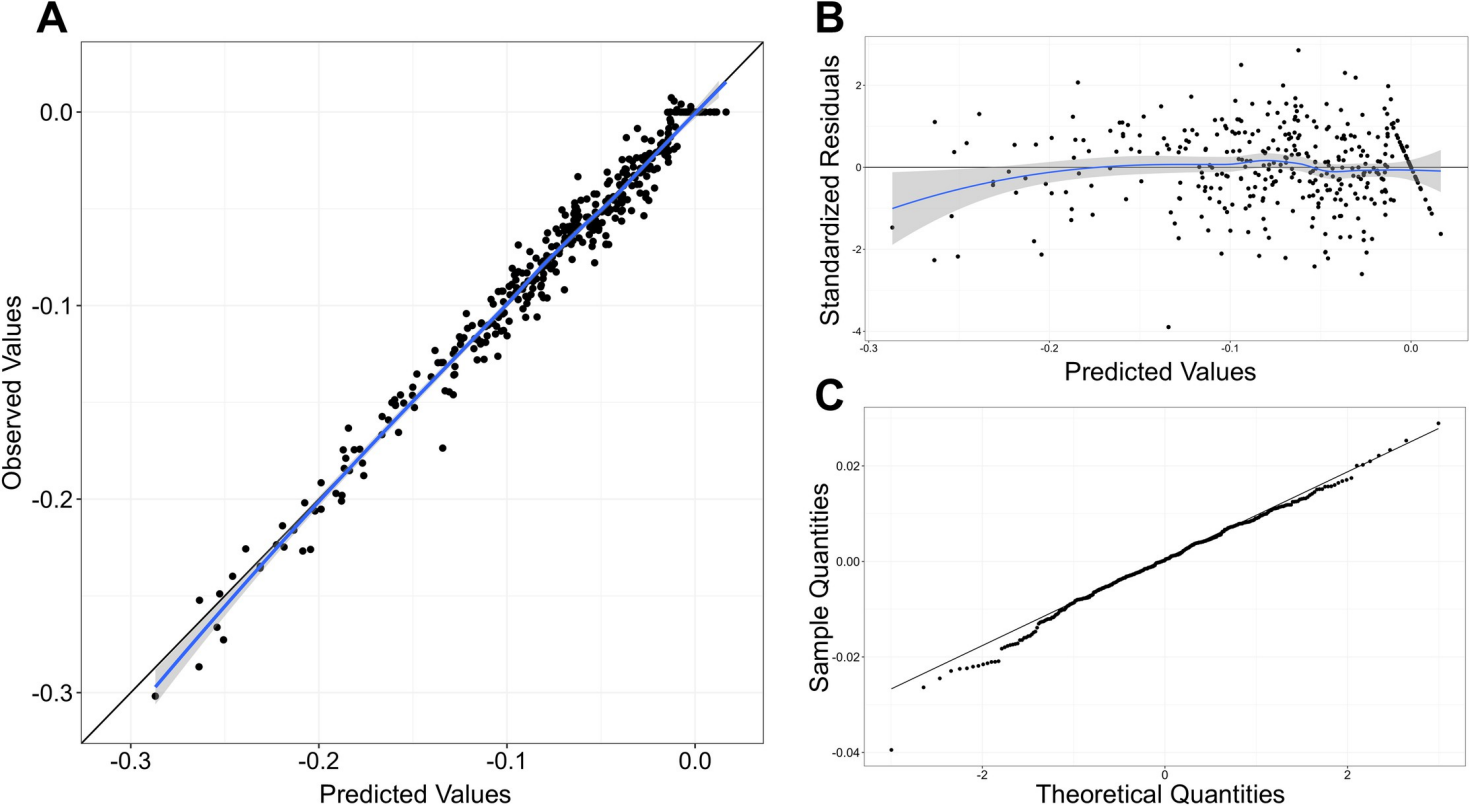
Diagnostic plots of the best-fit model. A. Goodness of fit plot. B. Standardized residuals plot. C. Normal Q-Q plot.

Due to sample error during sample collection and treatment, 13 data points out of a total of 378 data points were considered missing values and were removed in the modeling step (S2 and S3 Files). The model development and validation are shown in Table 1 and Fig 2. The inclusion of β_3_ did not improve the model’s performance (P-value = 0.14), and estimated β_3_ values were not significant (S1 Table). Therefore, model 3 was chosen for downstream analysis (Table 1).

**Table 1:** Linear mixed-effects model development for the stability study.

Model	AIC	BIC	logLik	Deviance	Chisq	Df	p-value
Model 1	-2077.0	-2053.6	1044.5	-2089.0			
Model 2	-2156.6	-2125.4	1086.3	-2172.6	83.7	2	<0.001
Model 3	-2185.1	-2150.0	1101.5	-2203.1	30.5	1	<0.001
Model 4	-2182.7	-2124.2	1106.4	-2212.7	9.7	6	0.14
Model 1: $log\left(C/C_{0}\right)=\mu +\theta -\left(\beta_{0}+\eta \right)*t+\varepsilon$ Model 2: $log\left(C/C_{0}\right)=\mu +\theta -\left(\beta_{0}+\beta_{1}*T+\eta \right)*t+\varepsilon$ Model 3: $log\left(C/C_{0}\right)=\mu +\theta -\left(\beta_{0}+\beta_{1}*T+\beta_{2}*C_{infu}+\eta \right)*t+\varepsilon$ Model 4: $log\left(C/C_{0}\right)=\mu +\theta -\left(\beta_{0}+\beta_{1}*T+\beta_{2}*C_{infu}+\beta_{3}*B+\eta \right)*t+\varepsilon$ logLik: log-likelihood, Chisq: chi-squared, Df: degree of freedom							

**Fig 3:**
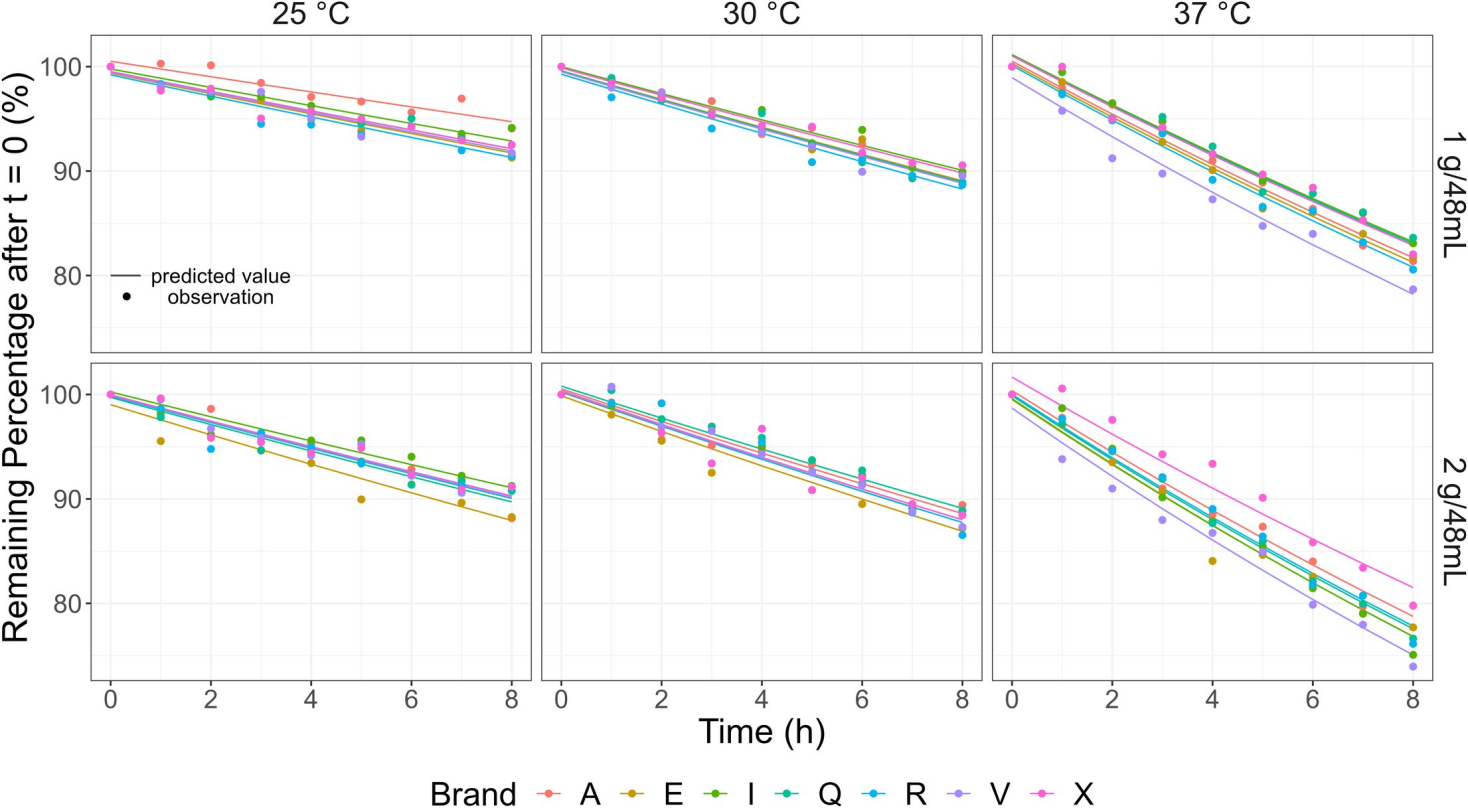
The remaining percentage of meropenem concentration for 7 brands by time under the investigated conditions.

The stability data of meropenem is depicted in Fig 3. Meropenem concentration in the prepared infusion solution declined gradually over time at different rates depending on the environment temperature and infusion concentration. No apparent differences in the degradation of meropenem among the 7 generic brands were encountered.

The estimated parameters of the final model are presented in Table 1. Time, temperature, and infusion concentration were significant factors in predicting the stability of the meropenem in the infusion solution. At 30°C and 37°C, its degradation rate constant increased significantly by 0.0045 and 0.0185 (h^-1^) compared to 25°C, respectively. In addition, the higher concentrated solution of 2 g/48mL was less stable than the solution diluted at 1 g/48 mL because a difference of 0.0042 (h^-1^) was observed in the degradation rate constant (Table 2).

**Table 2:** Estimated parameters for the stability study.

	Estimate	SE	95% Confidence Interval	p-value
**Fixed effects**				
Intercept (μ)	–0.0003	0.0014	–0.0024–0.0032	0.854
Time (β_0_)	0.0086	0.0006	0.0074–0.0099	<0.001
Temperature (β_1_)				
25°C	Reference			
30°C	0.0045	0.0007	0.0030–0.0059	<0.001
37°C	0.0185	0.0007	0.0171–0. 0201	<0.001
Infusion concentration (β_2_)				
1 g/48mL	Reference			
2 g/48mL	0.0042	0.0006	0.0030–0.0055	<0.001
**Random effects**				
ω_1_	0.0064		0.0041–0.0089	
ω_2_	0.0015		0.0008–0.0019	
σ	0.0101		0.0092–0.0108	
SE: Standard Error				

### PK/PD simulation studies

**Fig 4:**
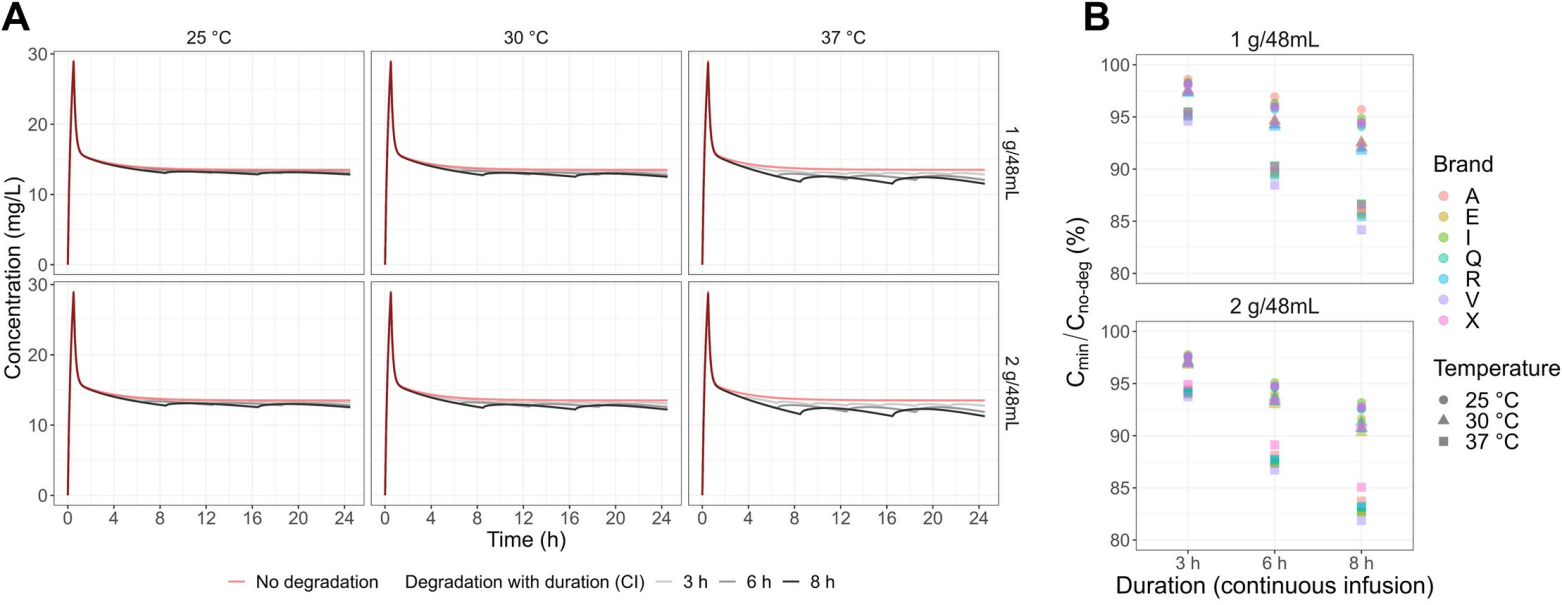
A. Meropenem simulated concentration profiles and B. The ratio of steady-state minimum concentration. Data were generated in different investigated conditions due to the decay process (C_min_) and steady-state concentration without degradation (C_no-deg_) for a typical patient (Clcr = 80.8 mL/min, body weight = 70 kg, serum albumin = 2.8 g/dL) applying continuous infusion with different infusion lengths (1 g every 3, 6, or 8 h) after a loading dose of 500 mg over 30 min.

The effect of the in-bottle degradation at different examined temperatures and infusion concentrations on the *in silico* pharmacokinetics profile of meropenem is shown in Fig 4A. The influence of temperature was more profound than the final concentrations for infusion. At 25°C and 30°C, regardless of the infusion concentration and bottle renewal interval, the simulated pharmacokinetic profiles showed a negligible deviation from the one simulated without degradation. At 37°C, such differences were more visually observed if the solution was infused for 8 h without renewal.

The ratio of steady-state minimum concentration created under different examined conditions due to the degradation process (C_min_) and steady-state concentration without degradation (C_no-deg_) was calculated to evaluate the extent of such an effect on the PTA. Deterministic simulation using individual-level stability parameters revealed no differences between brands in the C_min_/C_no-deg_ ratio (Fig 4B). When the length of infusion increased, this *in silico* ratio declined by 5–18%, depending mainly on the temperature.

**Fig 5:**
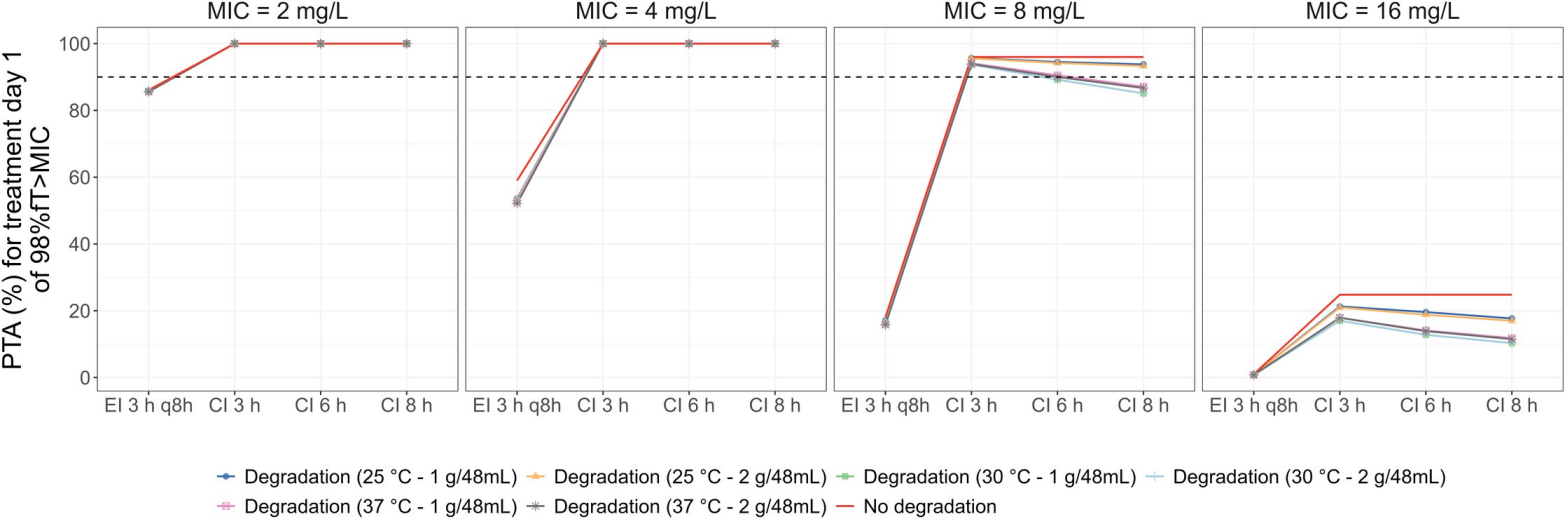
PTA treatment day 1 of 1 g every 8 h after LD of 500 mg over 30 min with various infusion lengths (3 h, 6 h, 8 h) and PK/PD target of 98%*f*T>MIC. Data were for a typical patient (Clcr = 80.8 mL/min, body weight = 70 kg, serum albumin = 2.8 g/dL) without and with degradation process at different conditions. Abbreviations: PTA, probability of target attainment; MIC, minimum inhibitory concentration.

The PTA of 98%*f*T>MIC in various investigated scenarios is depicted in Fig 5. CI showed more advance than EI as higher PTA was observed even when introducing meropenem degradation. For the CI strategy, the PTA was 100% with pathogens of MIC equal to 2 mg/L or 4 mg/L. With an elevated MIC of 8 mg/L, the PTA varied around the 90% threshold depending on the extent of degradation. The degradation in most tested conditions had a negligible impact on the PTA regardless of the administration modes. The most significant effect was observed in the case of 8 h CI and MIC of 16 mg/L, whose PTA was considerably below 90%.

**Fig 6:**
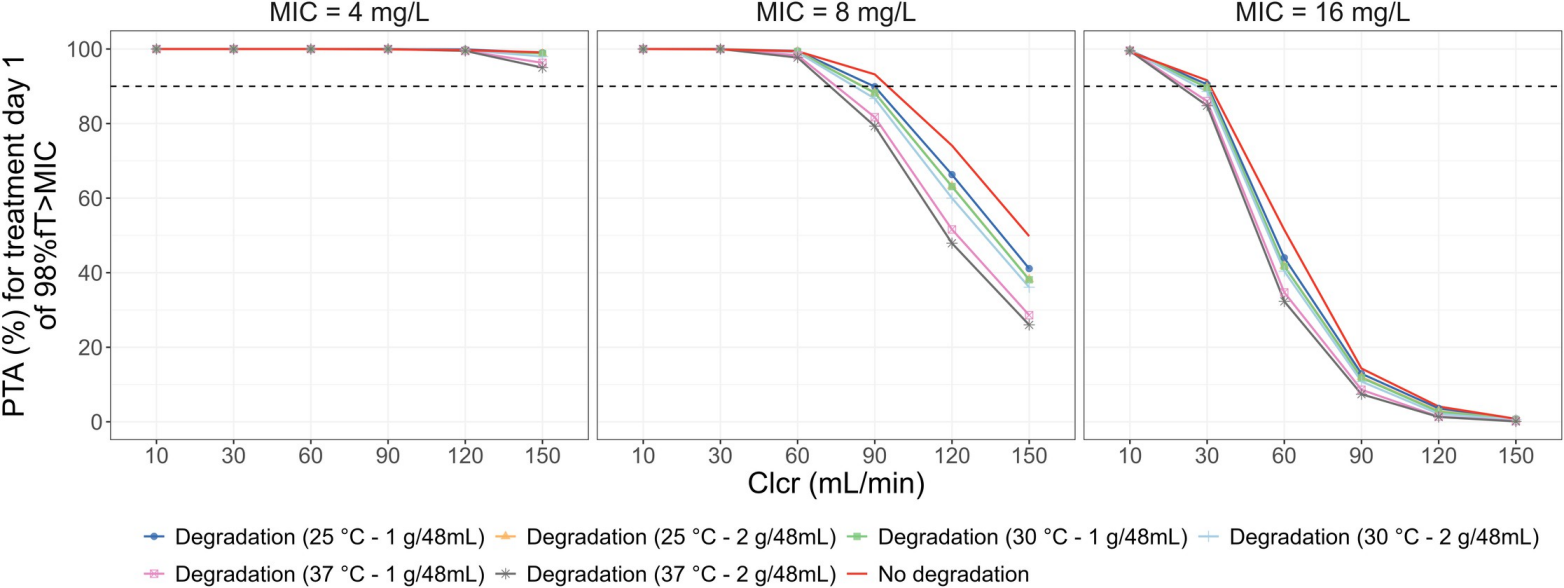
Impact of creatinine clearance on PTA of meropenem continuous infusion. PTA was evaluated at treatment day 1 with a target of 98%*f*T>MIC for Clcr-based grouped patients (weight of 70 kg and serum albumin of 2.8 g/dL). A continuous infusion with 1 g every 8 h after an LD of 500 mg over 30 min was used, with or without degradation process at different conditions. Abbreviations: PTA, probability of target attainment; MIC, minimum inhibitory concentration; Clcr, creatinine clearance.

Given that Clcr has been reported as the main covariate to predict meropenem clearance and achievability of the PTA [9, 10], it is crucial to consider the renal function in the Monte Carlo simulation (Fig 6). It should also be noted that the results need to be interpreted with caution in the case of CRRT patients where the relationship between Clcr and CRRT clearance is complex. In general, the PTA was significantly affected by renal function. Patients with augmented renal clearance (ARC) might not achieve the target with MIC of 8 mg/L or higher. With MIC of 4 mg/L, the target could be attained even with a high renal clearance, e.g., CLcr of 150 mL/min. Besides, the impact of degradation on the PTA could be observed in a specific situation (i.e., MIC of 8 mg/L and Clcr of over 90 mL/min).

**Fig 7:**
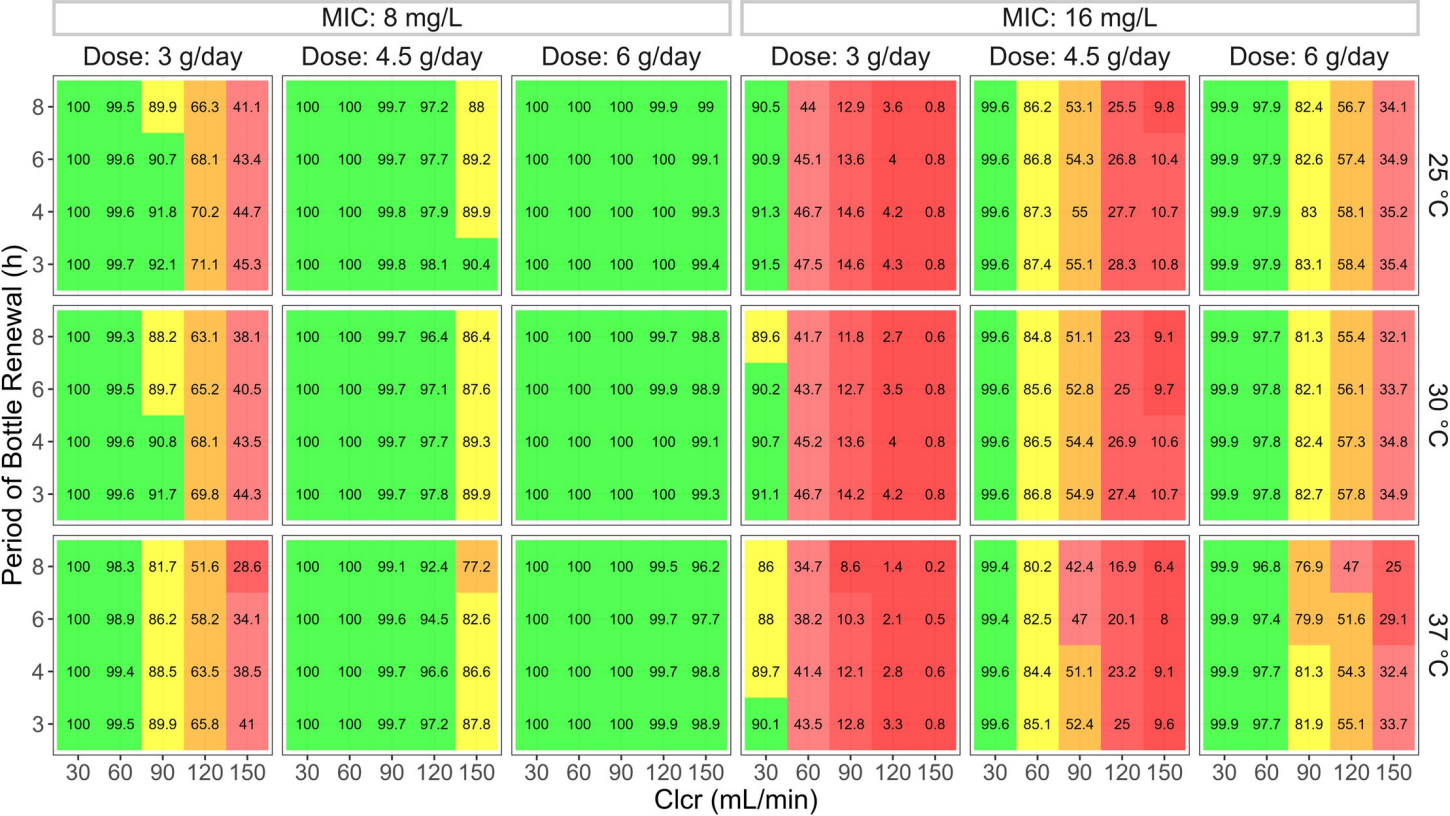
Impact of prescribed dose on PTA of meropenem continuous infusion. PTA was evaluated at treatment day 1 with a target of 98%*f*T>MIC for a total daily dose of 3, 4.5, and 6 g administered via continuous infusion after a loading dose of 500 mg over 30 min. Three temperatures (25, 30, and 37°C) and four periods of bottle renewal (3, 4, 6, and 8 h) were evaluated for elevated MIC pathogens (8 and 16 mg/L) and Clcr-based grouped patients (weight of 70 kg and serum albumin of 2.8 g/dL). Infusion concentration was kept at 1 g/48mL. Red, orange, yellow, and green colors are used when PTA < 50%, 50% ≤ PTA < 80%, 80% ≤ PTA < 90%, and PTA ≥ 90%, respectively. Abbreviations: PTA, probability of target attainment; MIC, minimum inhibitory concentration; Clcr, creatinine clearance.

The impact of the prescribed dose on CI was also examined and the simulation results are illustrated in Fig 7. The temperature was the only environmentally dependent factor analyzed among the two factors. The influence of meropenem’s degradation on the target attainment was negligible vis-à-vis the impact of the administered dose, Clcr of patients, and microbial MIC. As a result, infusion solution renewal earlier than 8 h showed a marginal impact on the PTA regardless of the renal function and microbiological susceptibility.

## Discussion

The degradation of meropenem resulted in a considerable reduction in its concentration of about 10–25% in the aqueous solution, losing the dose administered into the general circulation. The degradation rate depended on time, temperature, and concentration of the infusion solution, while no significant differences were found between the tested pharmaceutical products in this study. However, the meropenem degradation within 8 h stored under different conditions was likely to have a marginal impact on the PK/PD target attainment. This depended mainly on the prescribed dose of meropenem, the patient’s renal function, and microbial susceptibility. Therefore, the meropenem continuous infusion with bottle renewal every 8 h was practically feasible to target less susceptible isolates, especially in tropical areas.

Our stability study showed that the concentration of meropenem gradually decreased over time and remained below 80% after 8 h. The two main factors affecting the reduction rate were ambient temperature and infusion concentration. These findings aligned with studies examining similar conditions [17, 18, 21], except for the 2 g/48mL concentration at 25°C [18]. At this condition, the *in vitro* decomposition of meropenem was adequate for 4–6 h in the study of Delattre *et al*. [18], yet it could be acceptable for up to 8h, as observed in our study. In our study, seven meropenem generics had equivalent degradation profiles, which was in line with other published data [17, 21]. Therefore, meropenem products could be used in clinical settings without concern about the differences in their stability. According to our findings, consistent with those of other experimental studies, infusion concentration and temperature should be kept as low as possible to slow down the meropenem degradation rate during infusion [17, 18, 26, 32, 33]. Regarding the hard-to-control factor of temperature, previous data showed that cold temperatures resulted in insignificant degradation of meropenem concentration, even lasting up to 24 h [14, 15, 19, 34]. As a result, the infusion bag can be kept cold by sandwiching it between ice pouches throughout the infusion in the clinical setting to guarantee that the degradation of meropenem can be minimized. This could further facilitate the implementation of a full-day meropenem infusion. In addition, the infusion solution should be prepared with NaCl 0.9% [35] and administered in either daylight or darkness [21]. Limited data was reported on how the degradation of the prepared infusion solution could affect the patients’ simulated concentration-time profile [19]. Therefore, the impact of meropenem decay on PTA should be elucidated.

Due to the in-bottle degradation during infusion, the simulated concentration of meropenem produced by CI was not constant at steady-state. It reached the minimum level at the end of the infusion before the solution was renewed. Notably, steady state C_min_ compared to C_no-deg_ would be associated with a lower PTA of 98%*f*T>MIC. The Monte Carlo simulation also showed that degradation of meropenem in the infusion solution had a negligible impact on the PTA of patients and was only observed for certain circumstances, such as ARC patients and elevated MIC organisms. The renal function, the susceptibility of pathogens, and the prescribed dose of meropenem were demonstrated to be significant factors contributing to the attainment of the PK/PD target. Therefore, dose escalation was suggested to maximize the PTA and eliminate the impact of degradation. In addition, continuous infusion up to 8 h produced a significantly higher PTA value than the widely implemented 3 h EI despite the degradation effect. This finding enabled clinicians to be more confident with easy-to-administration 3 times per day of meropenem continuous infusion at the bedside in terms of PK/PD target attainment for prolonged infusion of such an unstable drug. Of note is that the up-to-date study had a consistent conclusion with our findings, which advocated the 8 h renewal of bottle infusion [20]. The benefits of prolonged infusion compared to intermittent infusion have been documented from clinical and microbiological aspects [36]. However, the clinical benefit of continuous infusion compared with extended infusion has yet to be proven, and further studies are required. Our study also paves the way for investigating the impact of degradation for other time-dependent antibiotics leveraging from available degradation information, especially when administrating at the ICU setting via continuous infusion [12, 35]. For example, imipenem is a potential candidate for such an approach because it showed the least stability and a higher probability of getting toxicity among beta-lactam antibiotics [12, 32].

Our study has several limitations that need to be taken into account. First, the examined conditions were limited to the temperature, infusion concentration, and a period of less than 8 h. However, meropenem infusion is routinely administered every 6 or 8 h for critical illness [24]. Therefore, stability up to 8 h was examined to mimic the clinical condition for evaluating the current practice. Second, the impact of controlled factors, including the type of reconstitution solution, light, and solution containers on the PTA of meropenem, was not elucidated. Furthermore, our study did not consider other potential confounding factors influencing the PTA of meropenem, such as patient variability in metabolism or drug-drug interaction. Third, metabolite levels were not measured to evaluate the *in vivo* degradation, and their impacts on the patient were not assessed. Fourth, suggestions have been based primarily on simulation results supported by experimental data. Therefore, more clinical data are warranted to validate the findings.

## Conclusions

By integrating stability information of meropenem into a popPK model, we have elucidated the impact of in-bottle meropenem’s degradation on the simulated PTA, thus supporting the implementation of continuous infusion at the bedside. The differences in the degradation of seven meropenem generics evaluated in our study were subtle. Meropenem CI with solution renewal every 8 h was advocated over EI, taken into account the degradation in the tropical conditions. However, in patients with augmented renal clearance and difficult-to-treat pathogens, meropenem degradation reduced PTA by an observable magnitude. Using a meropenem dose as high as 6 g per day could help ensure the PTA is higher than the 90% optimal level regardless of the degradation effect.

## Supporting information

S1 File Bioanalytical method development and validation. (DOCX)

S2 File Meropenem stability data. (XLSX)

S3 File R code for linear mixed-effects for the stability study. (PDF)

S4 File R code for PK/PD simulation. (PDF)

S1 Table Estimated parameters for the model 4 in the stability study. (DOCX)

## References

[1] Roberts JA, Abdul-Aziz MH, Lipman J, Mouton JW, Vinks AA, Felton TW, et al. Individualised antibiotic dosing for patients who are critically ill: challenges and potential solutions. The Lancet Infectious diseases. 2014;14(6):498–509. doi: 10.1016/S1473-3099(14)70036-2 24768475 PMC4181663

[2] Semret M, Haraoui L-P. Antimicrobial resistance in the tropics. Infectious Disease Clinics of North America. 2019;33(1):231–45. doi: 10.1016/j.idc.2018.10.009 30712764

[3] Blot SI, Pea F, Lipman J. The effect of pathophysiology on pharmacokinetics in the critically ill patient—concepts appraised by the example of antimicrobial agents. Advanced Drug Delivery Reviews. 2014;77:3–11. doi: 10.1016/j.addr.2014.07.006 25038549

[4] Timsit J-F, Bassetti M, Cremer O, Daikos G, De Waele J, Kallil A, et al. Rationalizing antimicrobial therapy in the ICU: a narrative review. Intensive care medicine. 2019;45(2):172–89. doi: 10.1007/s00134-019-05520-5 30659311

[5] Roberts JA, Kirkpatrick CM, Roberts MS, Robertson TA, Dalley AJ, Lipman J. Meropenem dosing in critically ill patients with sepsis and without renal dysfunction: intermittent bolus versus continuous administration? Monte Carlo dosing simulations and subcutaneous tissue distribution. Journal of Antimicrobial Chemotherapy. 2009;64(1):142–50. doi: 10.1093/jac/dkp139 19398460

[6] Mattioli F, Fucile C, Del Bono V, Marini V, Parisini A, Molin A, et al. Population pharmacokinetics and probability of target attainment of meropenem in critically ill patients. European journal of clinical pharmacology. 2016;72(7):839–48. doi: 10.1007/s00228-016-2053-x 27048201

[7] Ehmann L, Zoller M, Minichmayr IK, Scharf C, Huisinga W, Zander J, et al. Development of a dosing algorithm for meropenem in critically ill patients based on a population pharmacokinetic/pharmacodynamic analysis. International journal of antimicrobial agents. 2019;54(3):309–17. doi: 10.1016/j.ijantimicag.2019.06.016 31229669

[8] Truong AQ, Dao XC, Vu DH, Nguyen HA, Do THG, Tran NT, et al. Optimizing Meropenem in Highly Resistant *Klebsiella pneumoniae* Environments: Population Pharmacokinetics and Dosing Simulations in Critically Ill Patients. Antimicrobial agents and chemotherapy. 2022:e0032122. 36197095 10.1128/aac.00321-22 PMC9664861

[9] Cojutti P, Sartor A, Righi E, Scarparo C, Bassetti M, Pea F. Population pharmacokinetics of high-dose continuous-infusion meropenem and considerations for use in the treatment of infections due to KPC-producing *Klebsiella pneumoniae*. Antimicrobial agents and chemotherapy. 2017;61(10):e00794–17. 28760900 10.1128/AAC.00794-17 PMC5610526

[10] Minichmayr IK, Roberts JA, Frey OR, Roehr AC, Kloft C, Brinkmann A. Development of a dosing nomogram for continuous-infusion meropenem in critically ill patients based on a validated population pharmacokinetic model. Journal of Antimicrobial Chemotherapy. 2018;73(5):1330–9. doi: 10.1093/jac/dkx526 29425283

[11] Gatti M, Cojutti PG, Pea F. Impact of attaining aggressive vs. conservative PK/PD target on the clinical efficacy of beta-lactams for the treatment of Gram-negative infections in the critically ill patients: a systematic review and meta-analysis. Critical Care. 2024;28(1):123. doi: 10.1186/s13054-024-04911-5 38627763 PMC11020314

[12] Viaene E, Chanteux H, Servais H, Mingeot-Leclercq M-P, Tulkens PM. Comparative stability studies of antipseudomonal β-lactams for potential administration through portable elastomeric pumps (home therapy for cystic fibrosis patients) and motor-operated syringes (intensive care units). Antimicrobial agents and chemotherapy. 2002;46(8):2327–32. 12121900 10.1128/AAC.46.8.2327-2332.2002 PMC127357

[13] Fawaz S, Barton S, Whitney L, Swinden J, Nabhani-Gebara S. Stability of meropenem after reconstitution for administration by prolonged infusion. Hospital Pharmacy. 2019;54(3):190–6. doi: 10.1177/0018578718779009 31205331 PMC6535930

[14] Smith DL, Bauer SM, Nicolau DP. Stability of meropenem in polyvinyl chloride bags and an elastomeric infusion device. American Journal of Health-System Pharmacy. 2004;61(16):1682–5. doi: 10.1093/ajhp/61.16.1682 15540479

[15] Kuti JL, Nightingale CH, Knauft RF, Nicolau DP. Pharmacokinetic properties and stability of continuous-infusion meropenem in adults with cystic fibrosis. Clinical therapeutics. 2004;26(4):493–501. doi: 10.1016/s0149-2918(04)90051-3 15189746

[16] Berthoin K, Le Duff CS, Marchand-Brynaert J, Carryn S, Tulkens PM. Stability of meropenem and doripenem solutions for administration by continuous infusion. Journal of Antimicrobial Chemotherapy. 2010;65(5):1073–5. doi: 10.1093/jac/dkq044 20176578

[17] Carlier M, Stove V, Verstraete A, De Waele J. Stability of generic brands of meropenem reconstituted in isotonic saline. Minerva Anestesiologica. 2015;81(3):283–7. 25220554

[18] Delattre IK, Briquet C, Wallemacq P, Tulkens PM, Van Bambeke F. Comparative *in vitro* antimicrobial potency, stability, colouration and dissolution time of generics versus innovator of meropenem in Europe. International Journal of Antimicrobial Agents. 2020;55(1):105825. 31634551 10.1016/j.ijantimicag.2019.10.006

[19] Manning L, Wright C, Ingram PR, Whitmore TJ, Heath CH, Manson I, et al. Continuous infusions of meropenem in ambulatory care: clinical efficacy, safety and stability. PloS one. 2014;9(7):e102023. doi: 10.1371/journal.pone.0102023 25019523 PMC4096762

[20] Minichmayr IK, Friberg LE. Impact of continuous-infusion meropenem degradation and infusion bag changes on bacterial killing of Pseudomonas aeruginosa based on model-informed translation. International Journal of Antimicrobial Agents. 2024;64(2):107236. doi: 10.1016/j.ijantimicag.2024.107236 38851463

[21] Tomasello C, Leggieri A, Cavalli R, Di Terri G, D’avolio A. *In vitro* stability evaluation of different pharmaceutical products containing meropenem. Hospital Pharmacy. 2015;50(4):296–303. 26448659 10.1310/hpj5004-296 PMC4589882

[22] Agudelo M, Rodriguez C, Pelaez C, Vesga O. Even apparently insignificant chemical deviations among bioequivalent generic antibiotics can lead to therapeutic nonequivalence: the case of meropenem. Antimicrobial agents and chemotherapy. 2014;58(2):1005–18. doi: 10.1128/AAC.00350-13 24277034 PMC3910812

[23] Bajaj S, Singla D, Sakhuja N. Stability Testing of Pharmaceutical Products. Journal of Applied Pharmaceutical Science. 2012;2(3):129–38.

[24] Abdul-Aziz MH, Alffenaar JC, Bassetti M, Bracht H, Dimopoulos G, Marriott D, et al. Antimicrobial therapeutic drug monitoring in critically ill adult patients: a Position Paper. Intensive Care Med. 2020;46(6):1127–53. doi: 10.1007/s00134-020-06050-1 32383061 PMC7223855

[25] ICH harmonised tripartite guideline. Validation of analytical procedures: text and methodology Q2(R1) 2005. Available at: https://database.ich.org/sites/default/files/Q2(R1)%20Guideline.pdf.

[26] Mendez AS, Dalomo J, Steppe M, Schapoval EE. Stability and degradation kinetics of meropenem in powder for injection and reconstituted sample. Journal of pharmaceutical and biomedical analysis. 2006;41(4):1363–6. doi: 10.1016/j.jpba.2006.02.017 16533586

[27] Kuznetsova A, Brockhoff PB, Christensen RH. lmerTest package: tests in linear mixed effects models. Journal of statistical software. 2017;82:1–26.

[28] Ehmann L, Zoller M, Minichmayr IK, Scharf C, Maier B, Schmitt MV, et al. Role of renal function in risk assessment of target non-attainment after standard dosing of meropenem in critically ill patients: a prospective observational study. Critical Care. 2017;21(1):263. doi: 10.1186/s13054-017-1829-4 29058601 PMC5651591

[29] Yang N, Wang J, Xie Y, Ding J, Wu C, Liu J, et al. External Evaluation of Population Pharmacokinetic Models to Inform Precision Dosing of Meropenem in Critically Ill Patients. Frontiers in Pharmacology. 2022;13. doi: 10.3389/fphar.2022.838205 35662716 PMC9157771

[30] European Medicines Agency. Guideline on the use of pharmacokinetics and pharmacodynamics in the development of antimicrobial medicinal products 2016. Available at: https://www.ema.europa.eu/en/documents/scientific-guideline/guideline-use-pharmacokinetics-pharmacodynamics-development-antimicrobial-medicinal-products_en.pdf.

[31] Wang W, Hallow K, James D. A tutorial on RxODE: simulating differential equation pharmacometric models in R. CPT: pharmacometrics & systems pharmacology. 2016;5(1):3–10. doi: 10.1002/psp4.12052 26844010 PMC4728294

[32] Keel RA, Sutherland CA, Crandon JL, Nicolau DP. Stability of doripenem, imipenem and meropenem at elevated room temperatures. International journal of antimicrobial agents. 2011;2(37):184–5. doi: 10.1016/j.ijantimicag.2010.06.043 20702066

[33] Franceschi L, Cojutti P, Baraldo M, Pea F. Stability of generic meropenem solutions for administration by continuous infusion at normal and elevated temperatures. Therapeutic drug monitoring. 2014;36(5):674–6. doi: 10.1097/FTD.0000000000000054 24637698

[34] Grant EM, Zhong M-K, Ambrose PG, Nicolau DP, Nightingale CH, Quintiliani R. Stability of meropenem in a portable infusion device in a cold pouch. American Journal of Health-System Pharmacy. 2000;57(10):992–5. doi: 10.1093/ajhp/57.10.992 10832500

[35] Loeuille G, D’Huart E, Vigneron J, Nisse Y-E, Beiler B, Polo C, et al. Stability Studies of 16 Antibiotics for Continuous Infusion in Intensive Care Units and for Performing Outpatient Parenteral Antimicrobial Therapy. Antibiotics. 2022;11(4):458. doi: 10.3390/antibiotics11040458 35453211 PMC9030478

[36] Yu Z, Pang X, Wu X, Shan C, Jiang S. Clinical outcomes of prolonged infusion (extended infusion or continuous infusion) versus intermittent bolus of meropenem in severe infection: A meta-analysis. PloS one. 2018;13(7):e0201667. doi: 10.1371/journal.pone.0201667 30059536 PMC6066326

