# Supplementary material for: Optimization of meropenem continuous infusion based on Monte Carlo simulation integrating with degradation study
Source: PLoS One. 2024 Dec 23;19(12):e0313764. doi: 10.1371/journal.pone.0313764 (PMC11666027; doi:10.1371/journal.pone.0313764)
Supplement: S1 File — (DOCX) [file pone.0313764.s001.docx]

**Optimization of meropenem continuous infusion based on Monte Carlo simulation integrating with degradation study**

Nguyen Tran Nam Tien^1¶^, Vu Ngan Binh^2¶^, Pham Thi Thanh Ha^2^, Dang Thi Ngoc Lan^2^, Yong-Soon Cho^3^, Nguyen Phuoc Long^3^, Jae-Gook Shin^3,4^, Nguyen Hoang Anh (Jr)^1^, Truong Anh Quan^1^, Do Ngoc Tuan^5^, Nguyen Khac Tiep^6^, Pham The Thach^7^, Nguyen Hoang Anh^1,8^, Vu Dinh Hoa^1*^

^1^National Drug Information and Adverse Drug Reactions Monitoring Centre, Hanoi University of Pharmacy, Hanoi, Vietnam

^2^Department of Analytical Chemistry and Drug Quality Control, Hanoi University of Pharmacy, Hanoi, Vietnam

^3^Department of Pharmacology and PharmacoGenomics Research Center, Inje University College of Medicine, Busan, Republic of Korea

^4^ Department of Clinical Pharmacology, Inje University Busan Paik Hospital, Busan, Republic of Korea

^5^Department of Computing, Goldsmiths, University of London

^6^Department of Pharmaceutical BioTechnology, Hanoi University of Pharmacy, Hanoi, Vietnam

^7^Center for Intensive Care Medicine, Bach Mai Hospital, Hanoi, Vietnam

^8^Clinical Pharmacy and Drug Information Unit, Department of Pharmacy, Bach Mai Hospital, Hanoi, Vietnam

*Corresponding Author: Dr. Vu Dinh Hoa. Email: [vudinhhoa@gmail.com](mailto:vudinhhoa@gmail.com)

^¶^These authors contributed equally to this work.

# Bioanalytical method development and validation

## HPLC apparatus

The liquid chromatography was performed using an Agilent High-Performance Liquid Chromatography (HPLC) 1200 system with a Diode-Array Detection (DAD) detector. Data were processed with Chemstation data analysis software. The assay used an analytical Inertsustain C8 column (250 mm x 4.6 mm, 5 um particle size) and a C8 guard column (GL Sciences Inc., Japan). The mobile phase consisted of methanol and 0.1 % phosphoric acid with an isocratic ratio of 25:75 (v/v) and was pumped at a flow rate of 1.4 mL/min. The run time for one sample was 10 min and 50 µL samples were injected into the column. The detection wavelength was 310 nm.

## Method validation

The method was validated according to ICH guidelines in the following aspects: selectivity, linearity, repeatability, and recovery [1].

For selectivity, the following solutions were prepared: (i) standard solution containing meropenem at a concentration of approximately 80 mg/L, (ii) sample solution containing meropenem at a concentration of roughly 80 mg/L, (iii) distilled water, (iv) meropenem solution under several stresses: meropenem in HCl 0.1M; meropenem in NaOH 0.1M, meropenem in H_2_O_2_ 10 % solution, meropenem solution stressed under UV light 254 nm in 3 h; meropenem solution under thermal stress (50 ^o^C in 3 h). Analytical conditions were screened and selected so that all peaks of the degradation products of meropenem were separated from the meropenem peak.

The calibration curve was constructed by using the linear regression of the peak areas (y) versus the standard concentrations (x), ranging from about 40 mg/L to around 100 mg/L. The intra-day and inter-day repeatability were assessed based on the peak area of six replicated samples. Sample solutions (n = 3 for each quality control sample) were spiked with standard meropenem to achieve a solution containing meropenem of about 60 %, 80 %, and 100 % concentration of meropenem to evaluate the recovery. Since meropenem is unstable in aqueous solutions, the reference standard solutions (at known concentrations) and product samples were always freshly prepared on the day of analysis.

## Results

Meropenem peaks in the standard solution (i) and sample solution (ii) were both at about
4.0 minutes. Meropenem peak in the sample solution was fully separated from all degradation products. In the ranges under investigation, the method was determined to be linear (R^2^ = 0.998). The relative standard deviation (RSD) was lower than 2 % both for inter-day and intra-day precision (**S1 Table**). All the recovery percentages were between 98 - 102 % with an RSD of less than 2 %. (**S2 Table**).

**S1 Table.** Intra- and inter-day repeatability.

|  | **No.** | **Retention time (min)** | **Peak area** | **Content (%)** |
| --- | --- | --- | --- | --- |
| **Day 1** | 1 | 4.04 | 3283.5 | 103.5 |
|  | 2 | 4.03 | 3251.4 | 103.3 |
|  | 3 | 4.05 | 3325.3 | 103.1 |
|  | 4 | 4.05 | 3351.1 | 104.4 |
|  | 5 | 4.05 | 3356.7 | 104.5 |
|  | 6 | 4.06 | 3252.7 | 103.4 |
|  | **Average (n=6)** | | | **103.7** |
|  | **RSD (%) (n=6)** | | | **0.57** |
| **Day 2** | 1 | 4.03 | 3349.9 | 103.4 |
|  | 2 | 4.03 | 3402.6 | 104.1 |
|  | 3 | 4.03 | 3410.5 | 105.6 |
|  | 4 | 4.01 | 3408.7 | 105.6 |
|  | 5 | 4.01 | 3443.6 | 105.0 |
|  | 6 | 4.02 | 3380.2 | 103.9 |
|  | **Average (n=6)** | | | **104.6** |
|  | **RSD (%) (n=6)** | | | **0.91** |
| **Average (n=12)** | | | | **104.1** |
| **RSD (%) (n=12)** | | | | **0.85** |
| RSD: Relative Standard Deviation | | | | |

**S2 Table.** Recovery for 3 levels of concentration.

| **Sample code** | **Peak Area** | **C_measured_ (ppm)** | **C_spiked_ (ppm)** | **C_recovered_ (ppm)** | **% Recovered** | **Average  (%)** | **RSD (%)** |
| --- | --- | --- | --- | --- | --- | --- | --- |
| 60-1 | 1994.2 | 47.05 | 16.20 | 16.42 | 101.4 | 100.6 | 0.68 |
| 60-2 | 1987.4 | 46.89 | 16.20 | 16.26 | 100.4 |  |  |
| 60-3 | 1985.1 | 46.83 | 16.20 | 16.21 | 100.0 |  |  |
| 80-1 | 2726.5 | 64.23 | 32.40 | 32.96 | 101.7 | 99.9 | 1.62 |
| 80-2 | 2683.5 | 63.22 | 32.40 | 31.95 | 98.6 |  |  |
| 80-3 | 2694.2 | 63.47 | 32.40 | 32.20 | 99.4 |  |  |
| 100-1 | 3374.8 | 79.44 | 48.61 | 47.86 | 98.5 | 99.8 | 1.16 |
| 100-2 | 3417.8 | 80.45 | 48.61 | 48.87 | 100.6 |  |  |
| 100-3 | 3414.5 | 80.38 | 48.61 | 48.79 | 100.4 |  |  |
| RSD: Relative Standard Deviation | | | | | | | |

# References

1. ICH harmonised tripartite guideline. Validation of analytical procedures: text and methodology Q2(R1) 2005. Available at: <https://database.ich.org/sites/default/files/Q2%28R1%29%20Guideline.pdf>.
