# Supplementary material for: Optimization of meropenem continuous infusion based on Monte Carlo simulation integrating with degradation study
Source: PLoS One. 2024 Dec 23;19(12):e0313764. doi: 10.1371/journal.pone.0313764 (PMC11666027; doi:10.1371/journal.pone.0313764)
Supplement: S3 File — (PDF) [file pone.0313764.s003.pdf]

Optimization of meropenem continuous infusion based on Monte Carlo simulation integrating with degradation study

Linear mixed-effects model for stability study

Person-in-charge: Nguyen Tran Nam Tien, *National DI and ADR Centre, HUP*
Validation: Do Ngoc Tuan, *Department of Computing, Goldsmiths, University of London*
Supervision: Vu Dinh Hoa, *National DI and ADR Centre, HUP*

26 August, 2024

Contents

1 Load packages **2**

2 Pharmacokinetics equation **2**

3 Stability parameters **2**

4 Perform mixed-effects model **4**
4.1 Test to include random effects 4
4.2 Perfom mixed effects 6

5 Model Selection **6**
5.1 Perform LRT 6
5.2 Check normality of random effects 7
5.3 Obtain parameters and their 95CI 8

6 Model Diagnostics **9**
6.1 Derive prediction dataframe 9
6.2 Goodness of Fit Plot 10
6.3 Individual Fit 11
6.4 Standardized residuals 14

7 Extract parameters to introduce into popPK model **15**
7.1 Population-level parameters 15
7.2 `EBE` parameters 16

8 sessionInfo 18

1 Load packages

```
library(tidyverse)
library(readxl)
# library(lme4)
library(lmerTest)
library(performance)

library(foreach)
library(doParallel)

`%!in%` = Negate(`%in%`)
```

2 Pharmacokinetics equation

- First-order kinetic

$$C = C_0 * e^{-kt} * e^{\varepsilon}, \varepsilon \sim Norm(0, \sigma^2)$$
$$log(C) = log(C_0) - kt + \varepsilon, \varepsilon \sim Norm(0, \sigma^2)$$
$$log(C/C_0) = -k * t + \varepsilon, \varepsilon \sim Norm(0, \sigma^2)$$
$$log(C/C_0) = \mu + \theta - (\beta_0 + \beta 1 * Temp + \beta_2 * Conc + \beta_3 * Brand + \eta) * time + \varepsilon;$$
$$\theta \sim Norm(0, \omega_1^2), \eta \sim Norm(0, \omega_2^2), \varepsilon \sim Norm(0, \sigma^2)$$

3 Stability parameters

- Read from stability studies

```
mero.stability = readxl::read_excel(
  "C:/Users/Tien
  ↪  Nguyen/OneDrive/ADR/meropenem/GitHub/Mixed_effect_stability/meropenem_stability_tinydata.xlsx",
  sheet = "Data"
) %>%
  # Rename variable
  dplyr::rename(
    Brand = B, temp = T, conc = iC
  ) %>%
  # Add needed variables
  mutate(
    Conc = log(Conc), # transform to log scale
    y = log(Perc/100)
  ) %>%
  # Remove NA value in data
  na.omit()
```

```r
# Print 10 first rows
mero.stability %>%
  head(10) %>%
  knitr::kable()
```

Brand	conc	temp	time	C0	Conc	Perc	y
A	1g/48mL	25 °C	0	87.94	4.476655	100.00000	0.0000000
A	1g/48mL	25 °C	1	87.94	4.479494	100.28428	0.0028388
A	1g/48mL	25 °C	2	87.94	4.477905	100.12509	0.0012501
A	1g/48mL	25 °C	3	87.94	4.460953	98.44212	-0.0157014
A	1g/48mL	25 °C	4	87.94	4.447229	97.10030	-0.0294258
A	1g/48mL	25 °C	5	87.94	4.442651	96.65681	-0.0340035
A	1g/48mL	25 °C	6	87.94	4.431888	95.62202	-0.0447671
A	1g/48mL	25 °C	7	87.94	4.445588	96.94110	-0.0310666
A	1g/48mL	25 °C	8	87.94	4.415582	94.07551	-0.0610725
E	1g/48mL	25 °C	0	87.59	4.472667	100.00000	0.0000000

- Add ID variable. We had 42 (7 brands * 2 temperature * 2 tested concentration) IDs.

```r
mero.stability %>%
    filter(time == 0) %>%
    mutate(ID = row_number()) %>%
    select(Brand, temp, conc, ID) %>%
    right_join(mero.stability) -> mero.stability
```

- After log-transformed, concentration data have distributed normally.

```r
mero.stability %>%
    ggplot(aes(x = Conc)) + geom_density(size = 1.2) + theme_mero_sta() + labs(x =
    ↪  "Log(Concentration)")
```

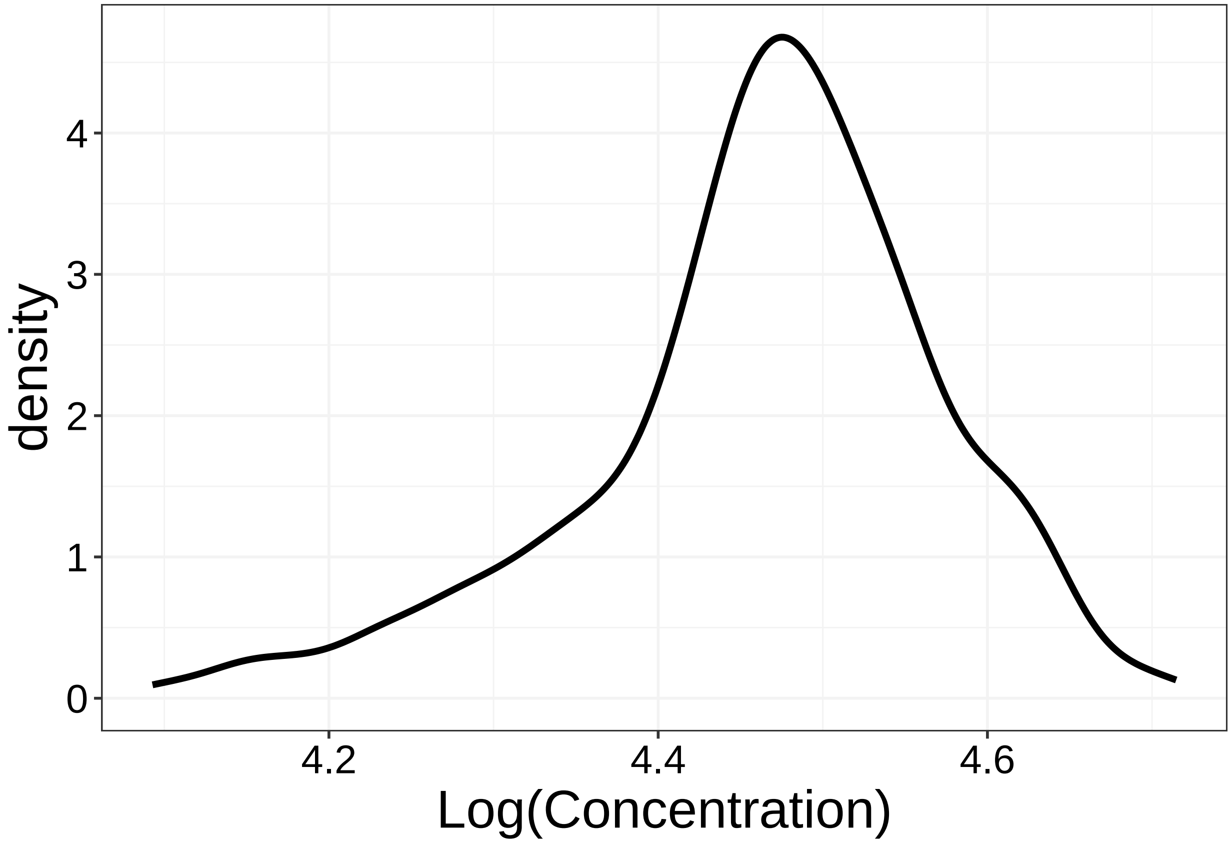

4 Perform mixed-effects model

4.1 Test to include random effects

```
m0.glm <- glm(y ~ 1, family = gaussian, data = mero.stability)
m0.lmer = lmer(y ~ 1 + (1 | ID), REML = T, data = mero.stability)  # random intercept
m0.lmer_2 = lmer(y ~ 1 + (0 + time | ID), REML = T, data = mero.stability)  #random slope
m0.lmer_3 = lmer(y ~ 1 + (1 + time | ID), REML = T, data = mero.stability)  # random
↪  intercept and random slope
```

- AIC values

```
AIC(logLik(m0.glm))
```

```
## [1] -996.9119
```

```
AIC(logLik(m0.lmer))
```

```
## [1] -1008.607
```

```
AIC(logLik(m0.lmer_2))
```

```
## [1] -1979.473
```

```
AIC(logLik(m0.lmer_3))
```

```
## [1] -1993.105
```

- Via base `anova()` function

```
anova(m0.lmer_3, m0.lmer_2, m0.lmer, m0.glm)
```

```
## Data: mero.stability
## Models:
## m0.glm: y ~ 1
## m0.lmer_2: y ~ 1 + (0 + time | ID)
## m0.lmer: y ~ 1 + (1 | ID)
## m0.lmer_3: y ~ 1 + (1 + time | ID)
##           npar      AIC      BIC  logLik deviance  Chisq Df Pr(>Chisq)
## m0.glm       2  -996.91  -989.11  500.46  -1000.9
## m0.lmer_2    3 -1991.38 -1979.68  998.69  -1997.4 996.47  1  < 2.2e-16 ***
## m0.lmer      3 -1017.45 -1005.75  511.72  -1023.5   0.00  0
## m0.lmer_3    5 -2004.57 -1985.07 1007.29  -2014.6 991.13  2  < 2.2e-16 ***
## ---
## Signif. codes:  0 '***' 0.001 '**' 0.01 '*' 0.05 '.' 0.1 ' ' 1
```

```
anova(m0.lmer_3, m0.lmer_2, m0.glm)
```

```
## Data: mero.stability
## Models:
## m0.glm: y ~ 1
## m0.lmer_2: y ~ 1 + (0 + time | ID)
## m0.lmer_3: y ~ 1 + (1 + time | ID)
##           npar      AIC      BIC  logLik deviance   Chisq Df Pr(>Chisq)
## m0.glm       2  -996.91  -989.11  500.46  -1000.9
## m0.lmer_2    3 -1991.38 -1979.68  998.69  -1997.4 996.472  1  < 2.2e-16 ***
## m0.lmer_3    5 -2004.57 -1985.07 1007.29  -2014.6  17.188  2  0.0001852 ***
## ---
## Signif. codes:  0 '***' 0.001 '**' 0.01 '*' 0.05 '.' 0.1 ' ' 1
```

```
anova(m0.lmer_3, m0.lmer, m0.glm)
```

```
## Data: mero.stability
## Models:
## m0.glm: y ~ 1
## m0.lmer: y ~ 1 + (1 | ID)
## m0.lmer_3: y ~ 1 + (1 + time | ID)
##           npar      AIC      BIC  logLik deviance   Chisq Df Pr(>Chisq)
```

```
## m0.glm       2  -996.91  -989.11  500.46  -1000.9
## m0.lmer      3 -1017.45 -1005.75  511.72  -1023.5  22.534  1  2.065e-06 ***
## m0.lmer_3    5 -2004.57 -1985.07 1007.29  -2014.6 991.127  2  < 2.2e-16 ***
## ---
## Signif. codes:  0 '***' 0.001 '**' 0.01 '*' 0.05 '.' 0.1 ' ' 1
```

=> `m0.lmer_3` (random intercept and random slope) has been selected for further investigation.

4.2 Perfom mixed effects

- Nested models comparison

```
set.seed(123)
fit4 = lmer(data = mero.stability, y ~ time + temp:time + conc:time + Brand:time +
    (1 + time | ID), REML = 0  # we want to compare nested models -> use maximum
↪   likelihood estimate (MLE) instead of restricted maximum likelihood
)

fit3 = lmer(data = mero.stability, y ~ time + temp:time + conc:time + (1 + time |
    ID), REML = 0)

fit2 = lmer(data = mero.stability, y ~ time + temp:time + (1 + time | ID), REML = 0)

fit1 = lmer(data = mero.stability, y ~ time + (1 + time | ID), REML = 0)
```

5 Model Selection

5.1 Perform LRT

- Via base `anova()` function

```
anova(fit4, fit3, fit2, fit1)
```

```
## Data: mero.stability
## Models:
## fit1: y ~ time + (1 + time | ID)
## fit2: y ~ time + temp:time + (1 + time | ID)
## fit3: y ~ time + temp:time + conc:time + (1 + time | ID)
## fit4: y ~ time + temp:time + conc:time + Brand:time + (1 + time | ID)
##      npar     AIC     BIC logLik deviance   Chisq Df Pr(>Chisq)
## fit1    6 -2077.0 -2053.6 1044.5  -2089.0
## fit2    8 -2156.6 -2125.4 1086.3  -2172.6 83.6599  2  < 2.2e-16 ***
## fit3    9 -2185.1 -2150.0 1101.5  -2203.1 30.4513  1  3.424e-08 ***
## fit4   15 -2182.7 -2124.2 1106.4  -2212.7  9.6536  6       0.14
## ---
## Signif. codes:  0 '***' 0.001 '**' 0.01 '*' 0.05 '.' 0.1 ' ' 1
```

=> Results showed that `fit3` have been a *best* model.

5.2 Check normality of random effects

Random intercept

```
r_int <- ranef(fit3)$ID$`(Intercept)`
qqnorm(r_int)
qqline(r_int)
```

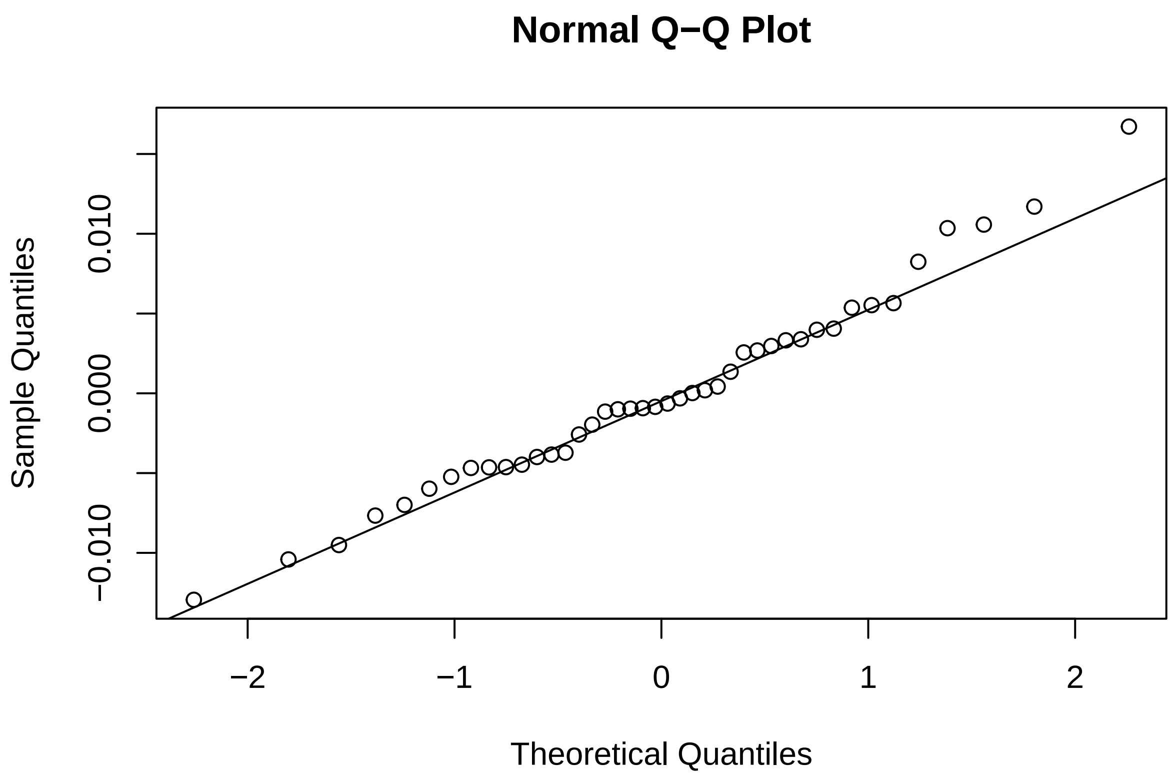


```
shapiro.test(r_int)
```

```
##
##  Shapiro-Wilk normality test
##
## data:  r_int
## W = 0.98155, p-value = 0.7214
```

Random slope

```
r_slope <- ranef(fit3)$ID$time
qqnorm(r_slope)
qqline(r_slope)
```

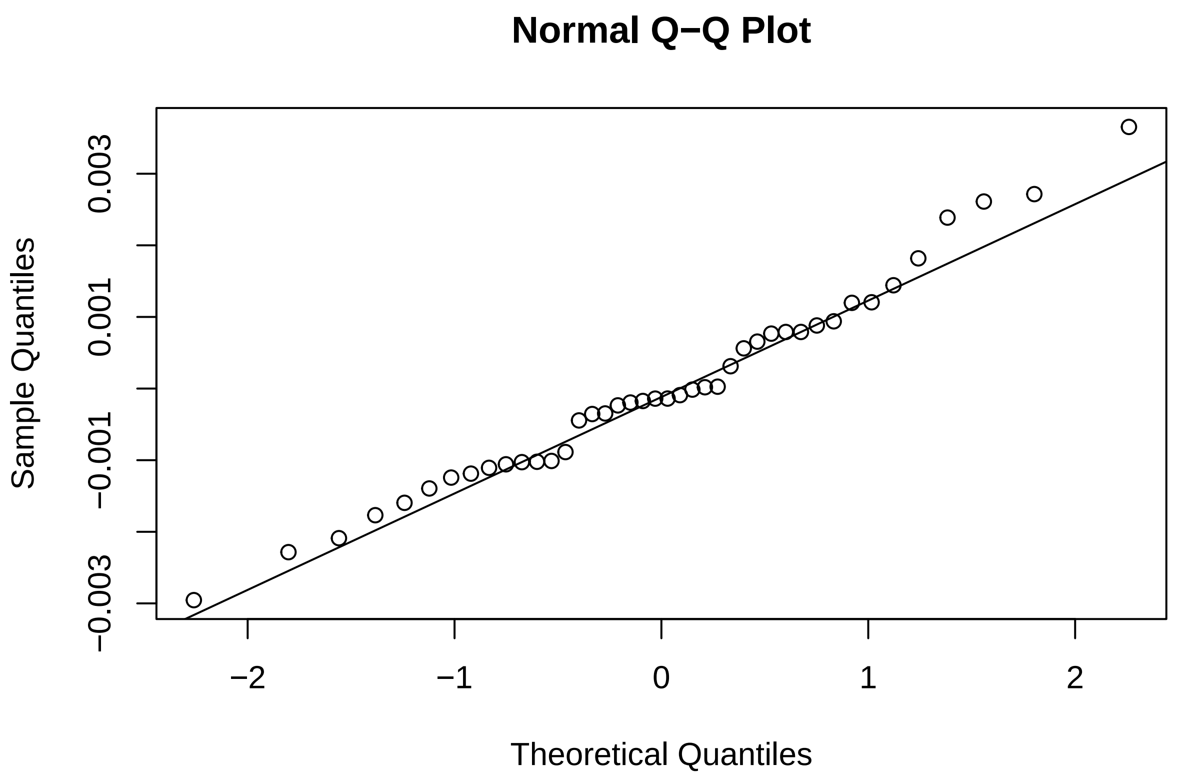


```
shapiro.test(r_slope)
```

```
##
##  Shapiro-Wilk normality test
##
## data:  r_slope
## W = 0.98017, p-value = 0.6682
```

5.3 Obtain parameters and their 95CI

```
summary(fit3)
```

```
## Linear mixed model fit by maximum likelihood . t-tests use Satterthwaite's
##   method [lmerModLmerTest]
## Formula: y ~ time + temp:time + conc:time + (1 + time | ID)
##    Data: mero.stability
##
##      AIC      BIC   logLik deviance df.resid
##  -2185.1  -2150.0   1101.5  -2203.1      356
##
## Scaled residuals:
##     Min      1Q  Median      3Q     Max
```

```
## -3.8977 -0.5500  0.0235  0.6619  2.8554 
## 
## Random effects:
##  Groups   Name        Variance  Std.Dev. Corr
##  ID       (Intercept) 4.119e-05 0.006418     
##           time        2.149e-06 0.001466 0.94
##  Residual             1.026e-04 0.010127     
## Number of obs: 365, groups:  ID, 42
## 
## Fixed effects:
##                    Estimate Std. Error         df t value Pr(>|t|)    
## (Intercept)      -0.0002568  0.0013867 42.6383244  -0.185    0.854    
## time             -0.0086083  0.0006084 41.9807850 -14.149  < 2e-16 ***
## time:temp30 °C   -0.0044789  0.0007469 42.3557144  -5.997  3.9e-07 ***
## time:temp37 °C   -0.0185050  0.0007407 41.0761198 -24.982  < 2e-16 ***
## time:conc2g/48mL -0.0041512  0.0006079 41.8556068  -6.828  2.6e-08 ***
## ---
## Signif. codes:  0 '***' 0.001 '**' 0.01 '*' 0.05 '.' 0.1 ' ' 1
## 
## Correlation of Fixed Effects:
##             (Intr) time   t:30°C t:37°C
## time         0.054                     
## tim:tmp30°C -0.008 -0.611              
## tim:tmp37°C  0.001 -0.611  0.497       
## tm:cnc2/48L  0.000 -0.505  0.011  0.001
```

- 95CI

```
set.seed(123)
confint.merMod(fit3, method = "boot", nsim = 5000)
```

```
##                          2.5 %       97.5 %
## .sig01            0.0040624650  0.008924078
## .sig02            0.3416622229  1.000000000
## .sig03            0.0008481927  0.001927924
## .sigma            0.0092394433  0.010818640
## (Intercept)      -0.0030205429  0.002434777
## time             -0.0098142426 -0.007394240
## time:temp30 °C   -0.0059262165 -0.002986678
## time:temp37 °C   -0.0199810835 -0.017035933
## time:conc2g/48mL -0.0053659961 -0.002934670
```

6 Model Diagnostics

6.1 Derive prediction dataframe

```
tibble(pred_value = fitted(fit3)) %>%
    bind_cols(mero.stability %>%
        mutate(ID = as.character(ID))) -> pred_df
```

6.2 Goodness of Fit Plot

```
fitted(fit3) %>%
    as_tibble() %>%
    rename(pred_value = value) %>%
    bind_cols(mero.stability) %>%
    ggplot(aes(pred_value, y)) + geom_point() + geom_abline(size = 0.5) +
    ↪  theme_mero_sta() +
    labs(y = "Observed Value", x = "Predicted Value") + geom_smooth(method = "loess") +
    scale_y_continuous(limits = c(-0.31, 0.02)) + scale_x_continuous(limits = c(-0.31,
    0.02))
```

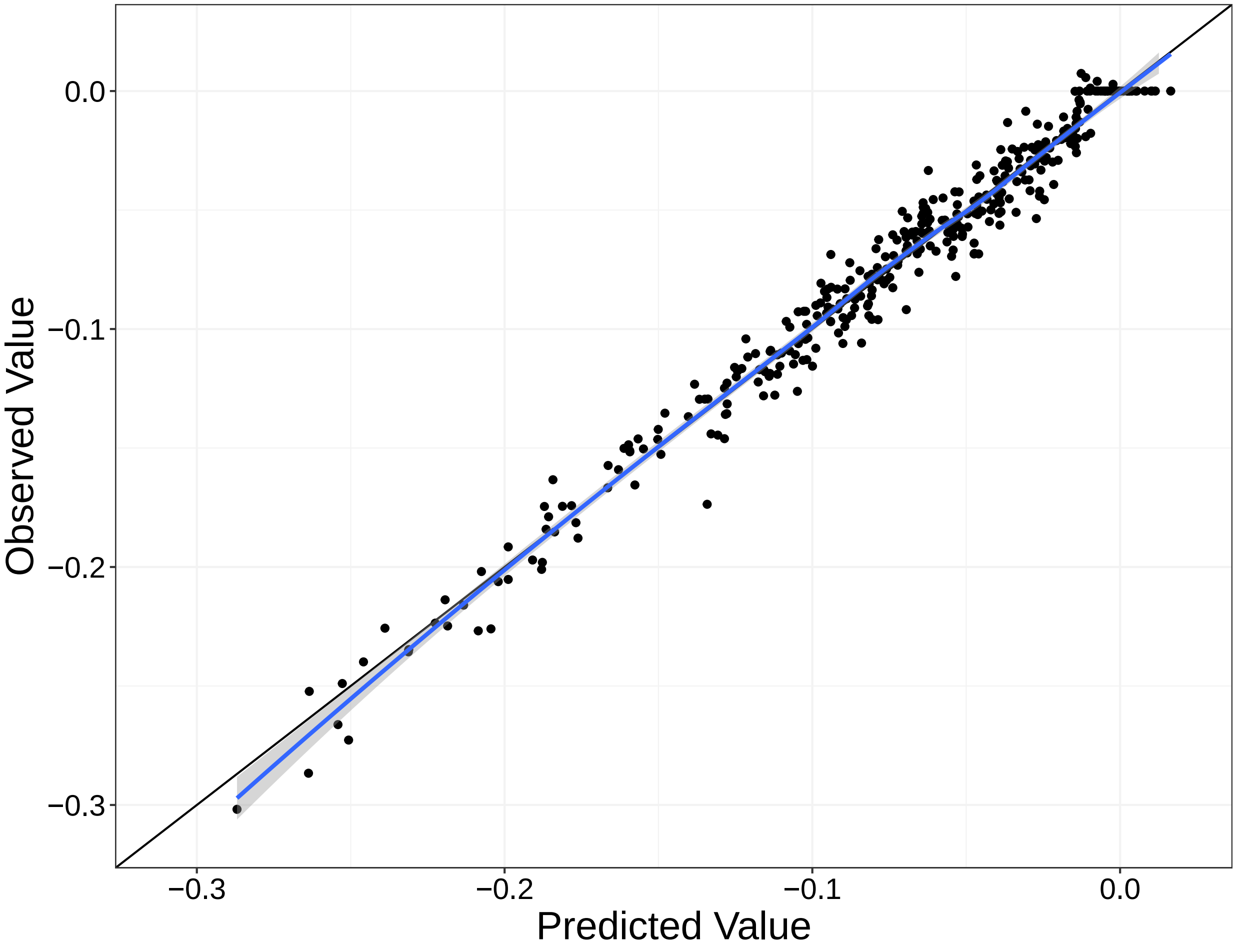


```
# ggsave('C:/Users/Tien
# N.T.Nguyen/OneDrive/ADR/meropenem/graph_report/20230226_GOF_mixeff.jpeg',
# width = 6.8, height = 6.8, dpi = 800)
```

6.3 Individual Fit

6.3.1 Via fitted

```
tibble(pred_value = fitted(fit3)) %>%
    bind_cols(mero.stability) %>%
    mutate(conc = ifelse(conc == "1g/48mL", "1 g/48mL", "2 g/48mL")) %>%
    ggplot(aes(x = time)) + facet_grid(conc ~ temp) + geom_line(aes(y = exp(pred_value) *
    100, color = Brand), size = 0.4) + geom_point(aes(y = exp(y) * 100, color = Brand)) +
    theme_bw() + theme(axis.title = element_text(size = 20), axis.text =
    ↪  element_text(size = 15),
    plot.caption = element_text(size = 18), strip.text = element_text(size = 18),
    legend.text = element_text(size = 18), legend.title = element_text(size = 19),
    legend.position = "bottom", panel.grid = element_line(colour = "#F3F3F3"),
    ↪  strip.background = element_blank()) +
    guides(color = guide_legend(nrow = 1)) + labs(x = "Time (h)", y = "Remaining
    ↪  Percentage after t = 0 (%)",
    color = "Brand") -> p

ano.predicted.text = data.frame(x = 2.2, y = 80/100 * 100, temp = "25 °C", conc = "1
↪  g/48mL")

ano.predicted.segment = data.frame(x = 0, y = 80/100 * 100, xend = 0.6, yend = 80/100 *
    100, temp = "25 °C", conc = "1 g/48mL")

ano.observation.text = data.frame(x = 2.2, y = 78/100 * 100, temp = "25 °C", conc = "1
↪  g/48mL")

ano.observation.point = data.frame(x = 0.32, y = 78/100 * 100, temp = "25 °C", conc = "1
↪  g/48mL")

p + geom_text(data = ano.predicted.text, aes(x = x, y = y), label = "predicted value",
    size = 4.2) + geom_text(data = ano.observation.text, aes(x = x, y = y), label =
    ↪  "observation",
    size = 4.2) + geom_segment(data = ano.predicted.segment, aes(x = x, y = y, xend =
    ↪  xend,
    yend = yend), alpha = 0.68) + geom_point(data = ano.observation.point, aes(x = x,
    y = y), alpha = 6)
```

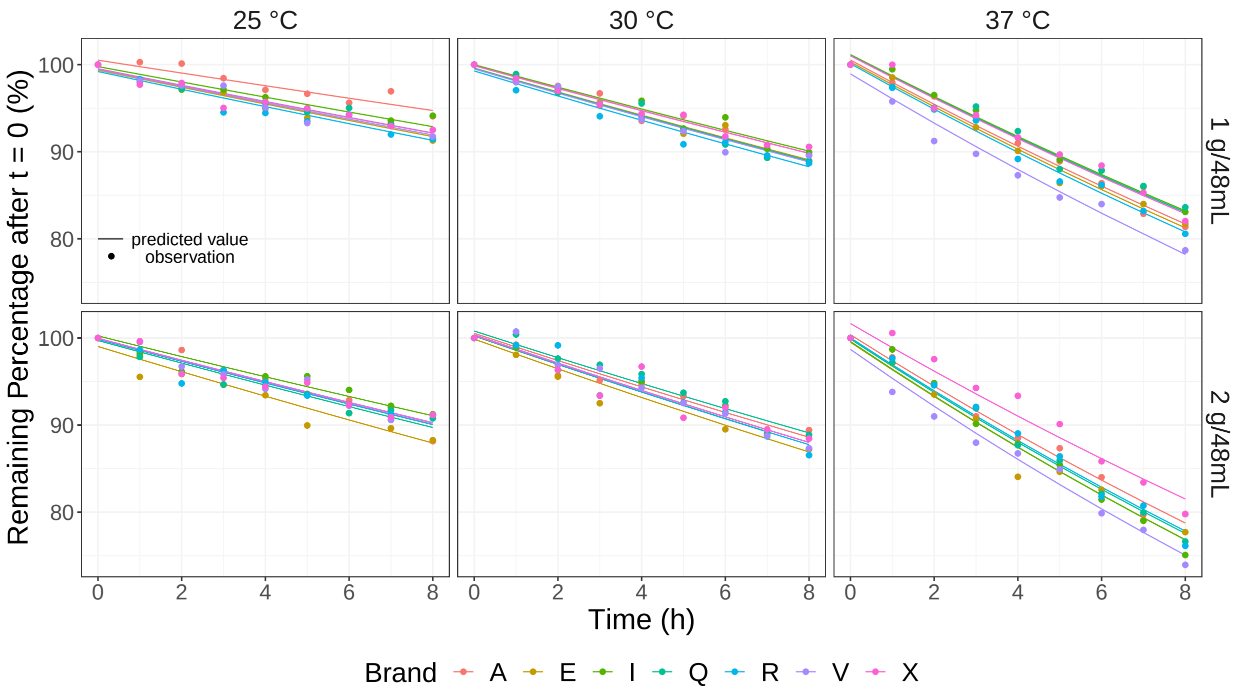


```
# ggsave('C:/Users/Tien
# Nguyen/OneDrive/ADR/meropenem/graph_report/Stability_EBE.png', width = 12,
# height = 6.8, dpi = 800)
```

6.3.2 Make extrapolation

```
#### Check several ways to make a prediction #### identical(predict(fit3),
#### fitted(fit3), predict(fit3, newdata = mero.stability), predict(fit3,
#### newdata = mero.stability, re.form = ~(1 + time | ID))) [1] TRUE

#### Make a new data frame ##### Create data.frame from 9 to 24 h first
time_points_exp = 9:24
data.frame(ID = rep(1:length(unique(mero.stability$ID)), each = length(time_points_exp)),
    time = rep(time_points_exp, length(unique(mero.stability$ID)))) -> mero.stability.exp

# Then merge with original data
mero.stability %>%
    bind_rows(mero.stability.exp) %>%
    arrange(ID) %>%
    group_by(ID) %>%
    fill(c(Brand, temp, conc, temp.value, C0), .direction = "down") -> mero.stability.exp

#### Make a prediction and used for visualization ####
tibble(pred_value = predict(fit3, newdata = mero.stability.exp)) %>%
    bind_cols(mero.stability.exp) %>%
    mutate(conc = ifelse(conc == "1g/48mL", "1 g/48mL", "2 g/48mL")) %>%
    ggplot(aes(x = time)) + facet_grid(conc ~ temp) + geom_line(aes(y = exp(pred_value) *
    100, color = Brand), size = 0.4) + geom_point(aes(y = exp(y) * 100, color = Brand)) +
```

```
    theme_bw() + scale_x_continuous(breaks = seq(0, 24, 4)) + theme(axis.title =
    ↪  element_text(size = 20),
    axis.text = element_text(size = 15), plot.caption = element_text(size = 18),
    strip.text = element_text(size = 18), legend.text = element_text(size = 18),
    legend.title = element_text(size = 19), legend.position = "bottom", panel.grid =
    ↪  element_line(colour = "#F3F3F3"),
    strip.background = element_blank()) + guides(color = guide_legend(nrow = 1)) +
    labs(x = "Time (h)", y = "Remaining Percentage after t = 0 (%)", color = "Brand") ->
        p

ano.predicted.text = data.frame(x = 2.2 * 3, y = 80/100 * 100, temp = "25 °C", conc = "1
↪  g/48mL")

ano.predicted.segment = data.frame(x = 0, y = 80/100 * 100, xend = 0.6 * 3, yend = 80/100
↪  *
    100, temp = "25 °C", conc = "1 g/48mL")

ano.observation.text = data.frame(x = 2.2 * 3, y = 75/100 * 100, temp = "25 °C",
    conc = "1 g/48mL")

ano.observation.point = data.frame(x = 0.32 * 3, y = 75/100 * 100, temp = "25 °C",
    conc = "1 g/48mL")

p + geom_text(data = ano.predicted.text, aes(x = x, y = y), label = "predicted value",
    size = 4.2) + geom_text(data = ano.observation.text, aes(x = x, y = y), label =
    ↪  "observation",
    size = 4.2) + geom_segment(data = ano.predicted.segment, aes(x = x, y = y, xend =
    ↪  xend,
    yend = yend), alpha = 0.68) + geom_point(data = ano.observation.point, aes(x = x,
    y = y), alpha = 6)
```

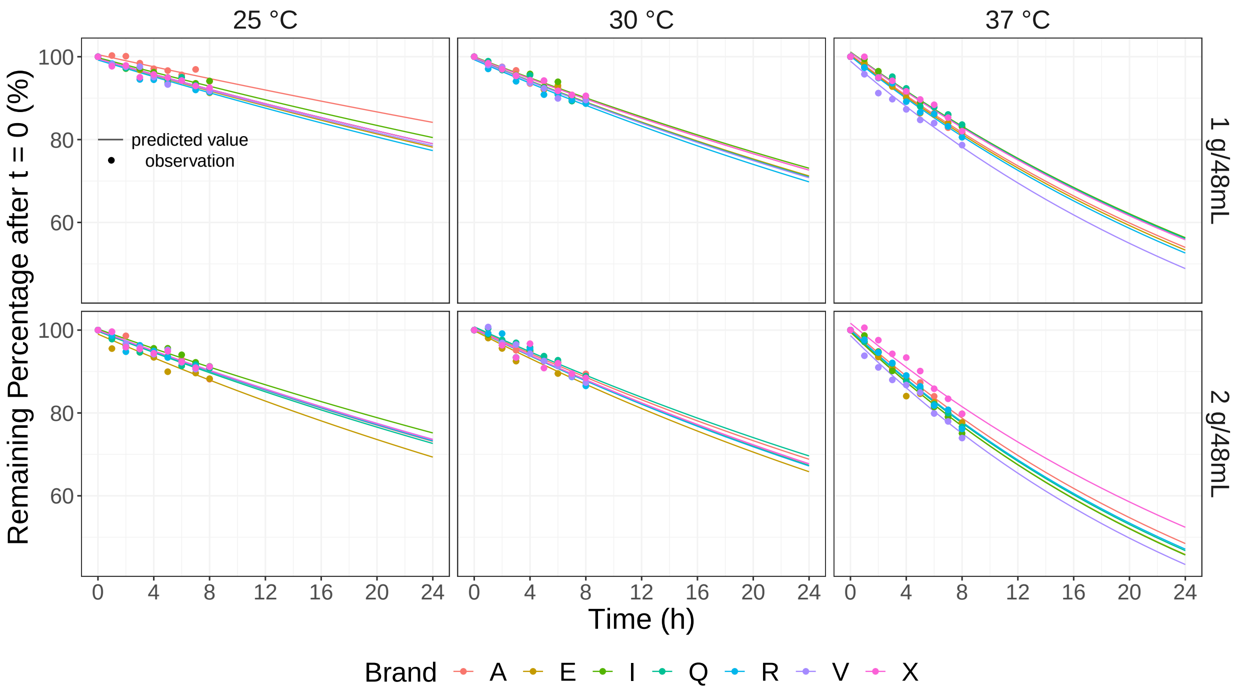

```
# ggsave('C:/Users/Tien
# Nguyen/OneDrive/ADR/meropenem/graph_report/Stability_EBE_exp.png', width =
# 12, height = 6.8, dpi = 800)
```

6.4 Standardized residuals

```
tibble(resid_value = resid(fit3, scaled = TRUE), pred_value = fitted(fit3)) %>%
    ggplot(aes(x = pred_value, y = resid_value)) + geom_point(size = 2.2) +
    ↪  geom_hline(yintercept = 0) +
    theme_mero_sta() + labs(x = "Predicted Value", y = "Standardised residuals") +
    geom_smooth(method = "loess")
```

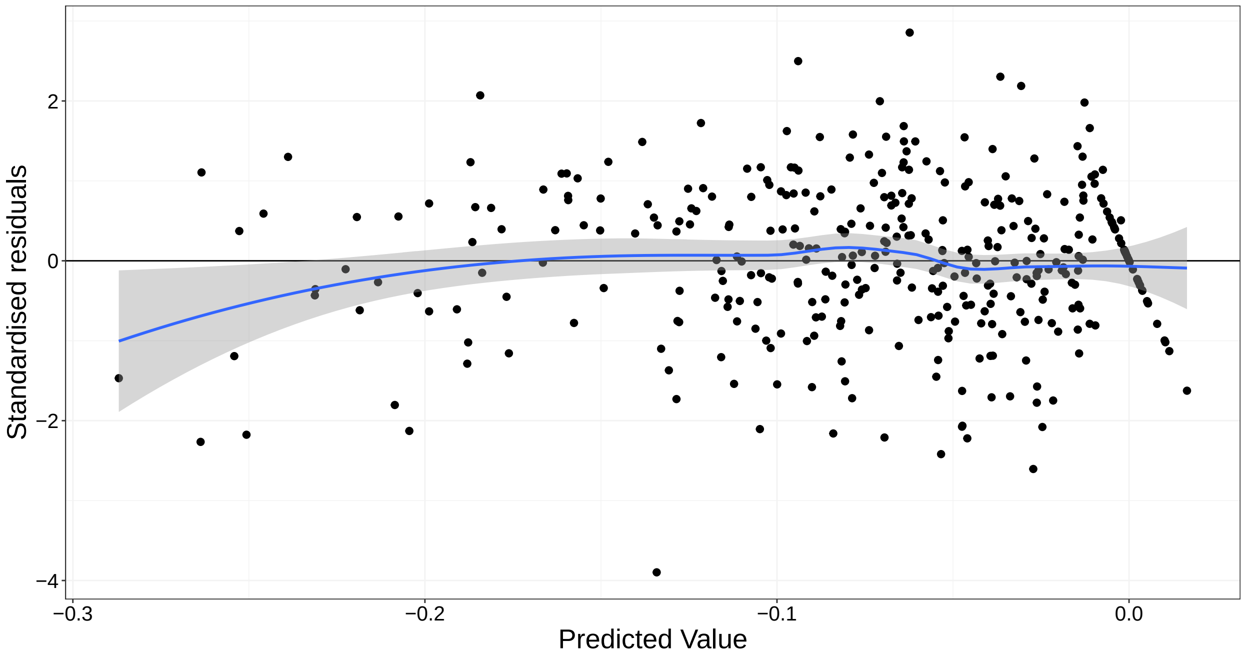


```
# ggsave('C:/Users/Tien
# N.T.Nguyen/OneDrive/ADR/meropenem/graph_report/20230226_Residual_mixeff.jpeg',
# width = 12.8, height = 6.8, dpi = 800)
```

- QQ plot

```
resid(fit3) %>%
    as_tibble() %>%
    ggplot(aes(sample = value)) + stat_qq() + stat_qq_line() + theme_mero_sta() +
    labs(x = "Theoretical Quantities", y = "Sample Quantities")
```

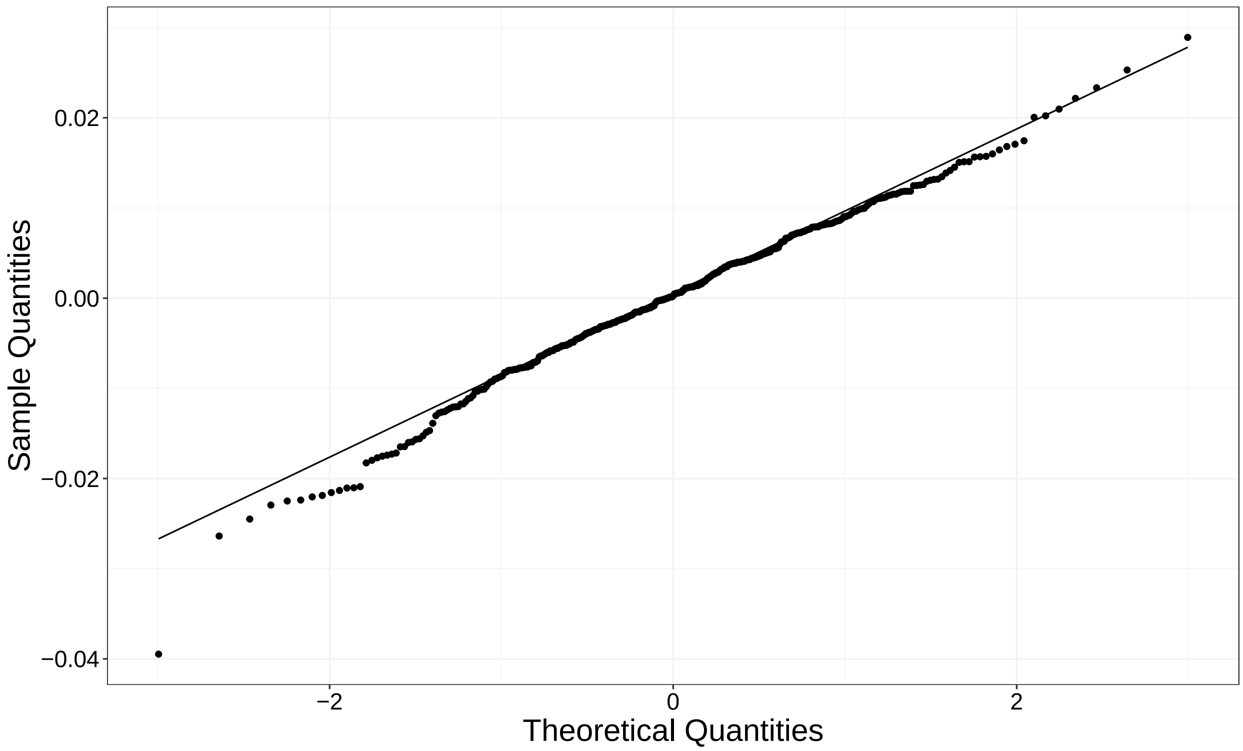


```
# ggsave('C:/Users/Tien
# N.T.Nguyen/OneDrive/ADR/meropenem/graph_report/20221209_QQ_mixeff.jpeg',
# width = 11.2, height = 6.8, dpi = 800)

# qqnorm(resid(fit2),pch=16) qqline(resid(fit2))
```

- RMSE

```
sqrt(mean(resid(fit3)^2))
```

```
## [1] 0.009546736
```

7 Extract parameters to introduce into popPK model

7.1 Population-level parameters

```
fitted_params = fit3@beta %>%
    t() %>%
    as_tibble()

tibble(Temp = rep(c("25 °C", "30 °C", "37 °C"), each = 2), Conc = rep(c("1g/48mL",
    "2g/48mL"), 3)) %>%
    mutate(intercept = fitted_params$V1, b0 = fitted_params$V2, b1 = ifelse(Temp ==
        25, 0, ifelse(Temp == 30, fitted_params$V3, fitted_params$V4)), b2 = ifelse(Conc
        ↪  ==
```

```r
        1, 0, fitted_params$V5)) -> df_stability_param

df_stability_param %>%
    knitr::kable()
```

Temp	Conc	intercept	b0	b1	b2
25 °C	1g/48mL	-0.0002568	-0.0086083	-0.018505	-0.0041512
25 °C	2g/48mL	-0.0002568	-0.0086083	-0.018505	-0.0041512
30 °C	1g/48mL	-0.0002568	-0.0086083	-0.018505	-0.0041512
30 °C	2g/48mL	-0.0002568	-0.0086083	-0.018505	-0.0041512
37 °C	1g/48mL	-0.0002568	-0.0086083	-0.018505	-0.0041512
37 °C	2g/48mL	-0.0002568	-0.0086083	-0.018505	-0.0041512

- Save as csv file

```r
# write.csv(df_stability_param, file = 'C:/Users/Tien
# Nguyen/OneDrive/ADR/meropenem/Mixed_effect_stability/stability_param_mixed_eff.csv',
# row.names = FALSE)
```

7.2 EBE parameters

```r
coef(fit3)$ID %>%
  mutate(ID = row.names(coef(fit3)$ID)) %>%
  rename(beta_time = time) %>%
  right_join(
    pred_df %>% dplyr::select(ID, Brand, temp, conc)
  ) %>%
  distinct(ID, .keep_all = TRUE) %>%
  select(Brand, temp, conc, everything()) %>%
  rename(
    Temp = temp, Conc = conc,
    intercept = `(Intercept)`,
    b0 = beta_time
  ) %>%
  # Add b1, b2
  mutate(
    # b1: Temperature
    b1 = case_when(
      Temp == "25 \u00B0C" ~ 0,
      Temp == "30 \u00B0C" ~ `time:temp30 °C`,
      Temp == "37 \u00B0C" ~ `time:temp37 °C`
    ),
    # b2: infusion concentration
    b2 = ifelse(Conc == "1g/48mL", 0, `time:conc2g/48mL`)
  ) %>%
  dplyr::select(Brand, Temp, Conc, intercept,   b0, b1, b2, ID) ->
↪   stability_param_mixed_eff_EBE_each_brand

# Show top 15 rows
stability_param_mixed_eff_EBE_each_brand %>%
```

```
  head(15) %>%
  knitr::kable()
```

Brand	Temp	Conc	intercept	b0	b1	b2	ID
A	25 °C	1g/48mL	0.0051087	-0.0074035	0.0000000	0	1
E	25 °C	1g/48mL	-0.0062346	-0.0100032	0.0000000	0	2
I	25 °C	1g/48mL	-0.0022188	-0.0089561	0.0000000	0	3
Q	25 °C	1g/48mL	-0.0047273	-0.0096307	0.0000000	0	4
R	25 °C	1g/48mL	-0.0079200	-0.0103768	0.0000000	0	5
V	25 °C	1g/48mL	-0.0054902	-0.0098515	0.0000000	0	6
X	25 °C	1g/48mL	-0.0049341	-0.0096190	0.0000000	0	7
A	30 °C	1g/48mL	-0.0011041	-0.0088034	-0.0044789	0	8
E	30 °C	1g/48mL	-0.0039788	-0.0094950	-0.0044789	0	9
I	30 °C	1g/48mL	-0.0000657	-0.0085820	-0.0044789	0	10
Q	30 °C	1g/48mL	-0.0042481	-0.0096361	-0.0044789	0	11
R	30 °C	1g/48mL	-0.0072489	-0.0102039	-0.0044789	0	12
V	30 °C	1g/48mL	-0.0048976	-0.0097155	-0.0044789	0	13
X	30 °C	1g/48mL	-0.0012184	-0.0087819	-0.0044789	0	14
A	37 °C	1g/48mL	0.0052705	-0.0074116	-0.0185050	0	15

```
# Export for PKPD simulation
# stability_param_mixed_eff_EBE_each_brand %>%
#   write.csv(
#     "C:/Users/Tien
↪  Nguyen/OneDrive/ADR/meropenem/Mixed_effect_stability/stability_param_mixed_eff_EBE_each_brand.csv",
#     row.names = FALSE
#   )
```

Show individual-level random effects to check

```
ranef(fit3)$ID %>%
    head(15) %>%
    knitr::kable()
```

(Intercept)	time
0.0053655	0.0012048
-0.0059778	-0.0013949
-0.0019620	-0.0003478
-0.0044705	-0.0010223
-0.0076632	-0.0017685
-0.0052334	-0.0012431
-0.0046773	-0.0010107
-0.0008473	-0.0001951
-0.0037220	-0.0008867
0.0001911	0.0000263
-0.0039913	-0.0010278
-0.0069921	-0.0015955
-0.0046409	-0.0011072
-0.0009616	-0.0001735
0.0055273	0.0011967

8 sessionInfo

```
sessionInfo()
```

```
## R version 4.2.3 (2023-03-15 ucrt)
## Platform: x86_64-w64-mingw32/x64 (64-bit)
## Running under: Windows 10 x64 (build 22631)
##
## Matrix products: default
##
## locale:
## [1] LC_COLLATE=English_United States.utf8
## [2] LC_CTYPE=English_United States.utf8
## [3] LC_MONETARY=English_United States.utf8
## [4] LC_NUMERIC=C
## [5] LC_TIME=English_United States.utf8
##
## attached base packages:
## [1] parallel  stats     graphics  grDevices utils     datasets  methods
## [8] base
##
## other attached packages:
##  [1] doParallel_1.0.17  iterators_1.0.14   foreach_1.5.2      performance_0.12.2
##  [5] lmerTest_3.1-3     lme4_1.1-34        Matrix_1.6-1       readxl_1.4.3
##  [9] lubridate_1.9.2    forcats_1.0.0      stringr_1.5.0      dplyr_1.1.2
## [13] purrr_1.0.2        readr_2.1.4        tidyr_1.3.0        tibble_3.2.1
## [17] ggplot2_3.4.3      tidyverse_2.0.0    knitr_1.43
##
## loaded via a namespace (and not attached):
##  [1] tidyselect_1.2.0    xfun_0.43           splines_4.2.3
##  [4] lattice_0.20-45     colorspace_2.1-0    vctrs_0.6.3
##  [7] generics_0.1.3      htmltools_0.5.6     mgcv_1.8-42
## [10] yaml_2.3.7          utf8_1.2.3          rlang_1.1.1
## [13] pillar_1.9.0        nloptr_2.0.3        glue_1.6.2
## [16] withr_2.5.0         lifecycle_1.0.3     munsell_0.5.0
## [19] gtable_0.3.3        cellranger_1.1.0    codetools_0.2-19
## [22] evaluate_0.21       labeling_0.4.2      tzdb_0.4.0
## [25] fastmap_1.1.1       fansi_1.0.4         highr_0.10
## [28] Rcpp_1.0.11         scales_1.2.1        formatR_1.14
## [31] farver_2.1.1        hms_1.1.3           digest_0.6.33
## [34] stringi_1.7.12      insight_0.20.3      bookdown_0.35
## [37] numDeriv_2016.8-1.1 grid_4.2.3          cli_3.6.1
## [40] tools_4.2.3         magrittr_2.0.3      pkgconfig_2.0.3
## [43] MASS_7.3-58.2       timechange_0.2.0    minqa_1.2.5
## [46] rmarkdown_2.24      rstudioapi_0.15.0   R6_2.5.1
## [49] boot_1.3-28.1       nlme_3.1-162        compiler_4.2.3
```
