# Supplementary material for: Optimization of meropenem continuous infusion based on Monte Carlo simulation integrating with degradation study
Source: PLoS One. 2024 Dec 23;19(12):e0313764. doi: 10.1371/journal.pone.0313764 (PMC11666027; doi:10.1371/journal.pone.0313764)
Supplement: S4 File — (PDF) [file pone.0313764.s004.pdf]

Optimization of meropenem continuous infusion based on Monte Carlo simulation integrating with degradation study

PK/PD simulation studies

Person-in-charge: Nguyen Tran Nam Tien, *National DI and ADR Centre, HUP*
Validation: Do Ngoc Tuan, *Department of Computing, Goldsmiths, University of London*
Supervision: Vu Dinh Hoa, *National DI and ADR Centre, HUP*

26 August, 2024

Contents

1 Load packages **2**

2 Model, omega, and V-Cov matrix **2**

2.1 Test for re-coding the model via a deterministic simulation 4

3 Test alternative method to introduce drug into `Central` comp **5**

3.1 Dosage regimen 5

3.2 Traditional method (Default) 5

3.3 Alternative method 6

4 No degradation simulation - stochastic simulation **8**

4.1 Dosage regimen for no-degra (3g/day) 8

4.2 Covariate 8

4.3 Perform simulation 9

5 Simulation to compare CI vs EI standard regimen **9**

5.1 No degradation simulation - stochastic simulation 9

5.2 Consider drug degradation 11

5.3 PTA calculation and visualisation 19

6 Simulation with changing Clcr **23**

6.1 No degradation simulation - stochastic simulation 23

6.2 Consider drug degradation 24

6.3 PTA visualization for Clcr changing 30

7 Simulation with changing Clcr & duration & dose **33**

7.1 Consider drug degradation 33

7.2 PTA visualization 41

8 Perfrom deterministic simulation **43**

8.1 Stability parameters 43

8.2 No degradaion 44

8.3 Degradation 45

8.4 Visualization 50

1 Load packages

```
library(tidyverse)
library(viridis)

library(RxODE)

library(foreach)
library(doParallel)
`%!in%` = Negate(`%in%`)
```

2 Model, omega, and V-Cov matrix

- Using a supplementary material in Ehmann2019 *et al.*
- Function to calculate `Var` from `RSE` function

```
rseVar <- function(est, rse) {
    return(abs(est) * rse/100)^2
}
```

- Deterministic model

```
Ehmann2019_deter <- RxODE({
    # @param
    tvCL = 9.25
    tvVc = 7.89
    tvQ = 28.4
    tvVp = 16.1

    CLCR_CL = 0.00977
    CLCR_INF = 154
    WT_Vc = 0.945
    ALB_Vp = -0.202
```

```
    # @pre
    if (Clcr <= CLCR_INF) {
        Cl = tvCL * (1 + CLCR_CL * (Clcr - 80.8))  #*exp(eta.IIV.Cl + eta.IOV.Cl)
    } else {
        Cl = CLCR_INF * (60/1000)  #*exp(eta.IIV.Cl + eta.IOV.Cl)
    }

    Vc = tvVc * (Wt/70)^WT_Vc  #*exp(eta.IIV.Vc)
    Q = tvQ
    Vp = tvVp * (1 + ALB_Vp * (Alb - 2.8))  #*exp(eta.IIV.Vp)

    # @dynamics
    d/dt(Central) = -Central * (Cl/Vc + Q/Vc) + Peri * Q/Vp
    d/dt(Peri) = Central * Q/Vc - Peri * Q/Vp

    # @derived
    Cp = Central/Vc
    # AMT_c = Central # Output automatically have both Central and Peri AMT_p =
    # Peri
})
```

- Stochastic model

```
Ehmann2019 <- RxODE({
    # @param
    tvCL = 9.25
    tvVc = 7.89
    tvQ = 28.4
    tvVp = 16.1

    CLCR_CL = 0.00977
    CLCR_INF = 154
    WT_Vc = 0.945
    ALB_Vp = -0.202

    # @pre
    if (Clcr <= CLCR_INF) {
        Cl = tvCL * (1 + CLCR_CL * (Clcr - 80.8)) * exp(eta.IIV.Cl + eta.IOV.Cl)
    } else {
        Cl = CLCR_INF * (60/1000)  #*exp(eta.IIV.Cl + eta.IOV.Cl)
    }

    Vc = tvVc * (Wt/70)^WT_Vc * exp(eta.IIV.Vc)
    Q = tvQ
    Vp = tvVp * (1 + ALB_Vp * (Alb - 2.8)) * exp(eta.IIV.Vp)

    # @dynamics
    d/dt(Central) = -Central * (Cl/Vc + Q/Vc) + Peri * Q/Vp
    d/dt(Peri) = Central * Q/Vc - Peri * Q/Vp

    # @derived
    Cp = Central/Vc
    # AMT_c = Central # Output automatically have both Central and Peri AMT_p =
```

```
    # Peri
})

#
Omega = lotri(eta.IIV.Cl ~ log((27.1/100)^2 + 1), eta.IIV.Vc ~ log((31.5/100)^2 +
    1), eta.IIV.Vp ~ log((16.9/100)^2 + 1), eta.IOV.Cl ~ log((12.5/100)^2 + 1))

# V-Cov matrix
thetaMat = lotri(tvCL ~ rseVar(9.25, 4.6), tvVc ~ rseVar(7.89, 11.9), tvQ ~ rseVar(28.4,
    16.1), tvVp ~ rseVar(16.1, 7.4), CLCR_CL ~ rseVar(0.00977, 9.2), CLCR_INF ~
    ↪  rseVar(154,
    6.9), WT_Vc ~ rseVar(0.945, 16.6), ALB_Vp ~ rseVar(-0.202, 36.6)  # don't use a
    ↪  negative number?
)
```

2.1 Test for re-coding the model via a deterministic simulation

```
no.patients = 3

df_covariate = data.frame(
  id = 1:no.patients,
  Wt = rep(70, no.patients),
  Clcr = c(154, 80.8, 24.8),  # uniform distribution
  Alb = rep(2.8, no.patients)
)
```

- Perform simulation for first dose

```
Ehmann2019_deter %>%
    et(amt = 1000, cmt = 1, dur = 1/2) %>%
    et(seq(0, 8, by = 0.1)) %>%
    et(id = 1:no.patients) %>%
    rxSolve(iCov = df_covariate, keep = c("Wt", "Alb", "Clcr")) %>%
    data.frame() %>%
    ggplot(aes(x = time, y = Cp)) + geom_line(aes(color = as.factor(id)), size = 1) +
    scale_color_manual(values = c("#F8766D", "#619CFF", "#00BA38"), labels = c("154
    ↪  mL/min",
        "80.8 mL/min", "24.8 mL/min")) + labs(subtitle = "First dosing interval",
    x = "Time (hours)", y = "Meropenem Concentration (mg/L)", color = "Creatinine
    ↪  clearance") +
    theme_bw()
```

- Perform simulation for 4 days

```
Ehmann2019_deter %>%
    et(amt = 1000, cmt = 1, dur = 1/2, ii = 8, addl = 4) %>%
    et(seq(0, 24, by = 0.1)) %>%
    et(id = 1:no.patients) %>%
    rxSolve(iCov = df_covariate, keep = c("Wt", "Alb", "Clcr")) %>%
```

```
    data.frame() %>%
    ggplot(aes(x = time, y = Cp)) + geom_line(aes(color = as.factor(id)), size = 1) +
    scale_color_manual(values = c("#F8766D", "#619CFF", "#00BA38"), labels = c("154
    ↪  mL/min",
        "80.8 mL/min", "24.8 mL/min")) + labs(subtitle = "First day of treatment",
    x = "Time (hours)", y = "Meropenem Concentration (mg/L)", color = "Creatinine
    ↪  clearance") +
    theme_bw()
```

3 Test alternative method to introduce drug into `Central` comp

3.1 Dosage regimen

```
duration = c(8, 6, 3, 0.5)

no.patients = 1

# use 6 cores
registerDoParallel(6)

list_dosage_regimen = foreach(i = 1:length(duration), .packages = c("tidyverse",
↪  "RxODE")) %dopar% {
  # loading dose for CI
  if (i == 1){
    et(amt = 500, cmt = 1, dur = 0.5) %>%
      et(time = 0.5, amt = 1000, addl = 3, duration = duration[i], ii = 8, cmt = 1) %>%
      et(id = 1:no.patients)

  # EI without LD
  } else {
    et(amt = 1000, addl = 3, duration = duration[i], ii = 8, cmt = 1) %>%
      et(id = 1:no.patients)
  }
}

# apply LD for all dose
list_dosage_regimen_all_LD = foreach(i = 1:length(duration), .packages = c("tidyverse",
↪  "RxODE")) %dopar% {
  et(amt = 500, cmt = 1, dur = 0.5) %>%  # 500 loading
    et(time = 0.5, amt = 1000, addl = 3, duration = duration[i], ii = 8, cmt = 1) %>%
    et(id = 1:no.patients)
}
```

3.2 Traditional method (Default)

```
foreach(i = 1:length(list_dosage_regimen), .packages = c("tidyverse", "RxODE")) %dopar%
    {
        Ehmann2019_deter %>%
```

```
            rxSolve(list_dosage_regimen[[i]] %>%
                et(seq(0, 24, by = 0.1)), iCov = data.frame(id = 1:1, Wt = 70, Clcr =
                ↪  80.8,
                Alb = 2.8), keep = c("Wt", "Alb", "Clcr")) %>%
            plot(Cp)
    }
```

3.3 Alternative method

```
list_dosage_regimen_al_method = foreach(i = 1:length(duration), .packages =
↪  c("tidyverse", "RxODE")) %dopar% {
  # loading dose for CI
  if (i == 1){
    et(amt = 500, cmt = 1, dur = 0) %>%
      et(time = 0.5, amt = 500, cmt = 1, dur = 0) %>%  # MD needs 500 mg instead of 1000
      ↪  because 500 mg above always inside Depot as 500 (see Depot Comp below for this
      ↪  dose)
      et(id = 1:no.patients)

  # EI without LD
  } else {
    et(amt = 1000, cmt = 1, dur = 0) %>%
      et(id = 1:no.patients)
  }
}

Ehmann2019_degra_deter = RxODE({
  # Test to include degradation parameter
  k = 0#0.008735422 + 0.01821446 + 0.004073311
  # @pre
  if(Clcr <= CLCR_INF){
    Cl = tvCL*(1 + CLCR_CL*(Clcr - 80.8))#*exp(eta.IIV.Cl + eta.IOV.Cl)
  } else {
    Cl = CLCR_INF*(60/1000)#*exp(eta.IIV.Cl + eta.IOV.Cl)
  }

  Vc = tvVc*(Wt/70)^WT_Vc#*exp(eta.IIV.Vc)
  Q = tvQ
  Vp = tvVp*(1 + ALB_Vp*(Alb - 2.8))#*exp(eta.IIV.Vp)

  # @dynamics
  d/dt(Depot) = -k*Depot

  d/dt(Central) = ifelse(
    # Strategy for Loading dose, note that duration = 0.5
    duration == 8 & time <= 0.5,
    Depot/0.5 - Central*(Cl/Vc + Q/Vc) + Peri*Q/Vp,

    # Strategy for CI
    ifelse(
      (duration == 8 & (
```

```
        (0.5 < time & time <= (duration + 0.5)) |
          ((interval*1 + 0.5) <= time & time <= (interval*1 + duration + 0.5)) |
          ((interval*2 + 0.5) <= time & time <= (interval*2 + duration + 0.5)) |
          ((interval*3 + 0.5) <= time & time <= (interval*3 + duration + 0.5))
      )) |
        # Strategy for EI
        (duration != 8 & (
          (time <= duration) |
            (interval*1 <= time & time <= (interval*1 + duration)) |
            (interval*2 <= time & time <= (interval*2 + duration)) |
            (interval*3 <= time & time <= (interval*3 + duration))
        )),
      Depot/duration - Central*(Cl/Vc + Q/Vc) + Peri*Q/Vp,  # drug from Depot into
↪  Central during infusion,
      -Central*(Cl/Vc + Q/Vc) + Peri*Q/Vp  # stop deliver
    )
  )

  d/dt(Peri) = Central*Q/Vc - Peri*Q/Vp

  # @derived
  Cp = Central/Vc
})

# Compare with traditional method
foreach(i = 1:length(duration), .packages = c("tidyverse", "RxODE")) %dopar% {
  param_degra = c(tvCL = 9.25,   # popPK param
                 tvVc = 7.89,
                 tvQ = 28.4,
                 tvVp = 16.1,

                 CLCR_CL = 0.00977,
                 CLCR_INF = 154,
                 WT_Vc = 0.945,
                 ALB_Vp  = -0.202,

                 interval = 8,
                 duration = duration[i])


  Ehmann2019_degra_deter %>%
    rxSolve(
      list_dosage_regimen_al_method[[i]] %>% et(seq(0, 24, by = 0.1)),
      param_degra,
      iCov = data.frame(
        id = 1:1, Wt = 70, Clcr = 80.8, Alb = 2.8
      ),
      keep = c("Wt", "Alb", "Clcr")
    ) %>%
    # Check the pattern with visualization
    plot(Cp)
    # Or print the data.frame to check
    #data.frame()
```

```
}
```

Conclusion 1: Model and method are ready to further evaluation

4 No degradation simulation - stochastic simulation

4.1 Dosage regimen for no-degra (3g/day)

```
duration = c(8, 6, 3, 3)
interval = c(8, 6, 3, 8)
amt_vec = c(1000, 750, 375, 1000)
interval_minus_duration = interval - duration

# Define num of patients for stochastic simulation
no.patients = 1000

# use 6 cores
registerDoParallel(6)

# traditional method (no degradation): only LD for CI, not for EI
# list_dosage_regimen = foreach(i = 1:length(duration), .packages = c("tidyverse",
↪  "RxODE")) %dopar% {
#   # loading dose for CI
#   if (i == 1){
#     et(amt = 500, cmt = 1, dur = 0.5) %>%  # 500 loading for CI
#       et(time = 0.5, amt = 1000, addl = 3, duration = duration[i], ii = 8, cmt = 1) %>%
#       et(id = 1:no.patients)
#
#   # EI without LD
#   } else {
#     et(amt = 1000, addl = 3, duration = duration[i], ii = 8, cmt = 1) %>%
#       et(id = 1:no.patients)
#   }
# }

# apply LD for all dose (just for no-degradation)
list_dosage_regimen_all_LD = foreach(i = 1:length(duration), .packages = c("tidyverse",
↪  "RxODE")) %dopar% {
  et(amt = 500, cmt = 1, dur = 0.5) %>%  # 500 loading
    et(time = 0.5, amt = amt_vec[i], addl = 3, duration = duration[i], ii = 8, cmt = 1)
    ↪  %>%
    et(id = 1:no.patients)
}
```

4.2 Covariate

- Consider a “typical” case in Ehmann2019 *et al.*

```
df_covariate = data.frame(id = 1:no.patients, Wt = rep(70, no.patients), Clcr = 80.8,
    Alb = 2.8)
```

4.3 Perform simulation

- Apply LD for all dose

```
# use 6 cores
registerDoParallel(6)

set.seed(123)
rxSetSeed(123)
no_degra_simu_list = foreach(i = 1:length(list_dosage_regimen_all_LD), .packages =
↪   c("tidyverse",
    "RxODE")) %dopar% {
    Ehmann2019 %>%
        rxSolve(list_dosage_regimen_all_LD[[i]] %>%
            et(seq(0, 24, by = 0.1)), iCov = df_covariate, keep = c("Wt", "Alb",
            "Clcr"), omega = Omega, cores = 6) %>%
        data.frame() %>%
        mutate(duration = duration[i], group = "No degradation")
}
```

5 Simulation to compare CI vs EI standard regimen

5.1 No degradation simulation - stochastic simulation

5.1.1 Model

```
Ehmann2019 <- RxODE({
    # @param
    tvCL = 9.25
    tvVc = 7.89
    tvQ = 28.4
    tvVp = 16.1

    CLCR_CL = 0.00977
    CLCR_INF = 154
    WT_Vc = 0.945
    ALB_Vp = -0.202

    # @pre
    if (Clcr <= CLCR_INF) {
        Cl = tvCL * (1 + CLCR_CL * (Clcr - 80.8)) * exp(eta.IIV.Cl + eta.IOV.Cl)
    } else {
        Cl = CLCR_INF * (60/1000)  #*exp(eta.IIV.Cl + eta.IOV.Cl)
    }
```

```
    Vc = tvVc * (Wt/70)^WT_Vc * exp(eta.IIV.Vc)
    Q = tvQ
    Vp = tvVp * (1 + ALB_Vp * (Alb - 2.8)) * exp(eta.IIV.Vp)

    # @dynamics
    d/dt(Central) = -Central * (Cl/Vc + Q/Vc) + Peri * Q/Vp
    d/dt(Peri) = Central * Q/Vc - Peri * Q/Vp

    # @derived
    Cp = Central/Vc
    # AMT_c = Central # Output automatically have both Central and Peri AMT_p =
    # Peri
})

#
Omega = lotri(eta.IIV.Cl ~ log((27.1/100)^2 + 1), eta.IIV.Vc ~ log((31.5/100)^2 +
    1), eta.IIV.Vp ~ log((16.9/100)^2 + 1), eta.IOV.Cl ~ log((12.5/100)^2 + 1))
```

5.1.2 Dosage regimen for no-degra (3g/day)

```
duration = c(8, 6, 3, 3)
interval = c(8, 6, 3, 8)
amt_vec = c(1000, 750, 375, 1000)
interval_minus_duration = interval - duration

# Define num of patients for stochastic simulation
no.patients = 1000

# use 6 cores
registerDoParallel(6)

# apply LD for all dose (just for no-degradation)
list_dosage_regimen_all_LD = foreach(i = 1:length(duration), .packages = c("tidyverse",
↪   "RxODE")) %dopar% {
  et(amt = 500, cmt = 1, dur = 0.5) %>%  # 500 loading
    et(time = 0.5, amt = amt_vec[i], addl = 8, duration = duration[i], ii = interval[i],
    ↪   cmt = 1) %>%
    et(id = 1:no.patients)
}
```

5.1.3 Covariate

- Consider a "typical" case in Ehmann2019 *et al.*

```
df_covariate = data.frame(id = 1:no.patients, Wt = rep(70, no.patients), Clcr = 80.8,
    Alb = 2.8)
```

5.1.4 Perform simulation

- Apply LD for all dose

```
# use 6 cores
registerDoParallel(6)

no_degra_simu = foreach(i = 1:length(list_dosage_regimen_all_LD), .packages =
↪  c("tidyverse",
    "RxODE"), .combine = "rbind") %dopar% {
    # to guarantee that all ID were identical between regimen -> but bias are
    # able to introduce -> the best way are using of no-degradation strategy
    set.seed(123)

    Ehmann2019 %>%
        rxSolve(list_dosage_regimen_all_LD[[i]] %>%
            et(seq(0, 24, by = 0.1)), iCov = df_covariate, keep = c("Wt", "Alb",
            "Clcr"), omega = Omega, cores = 6) %>%
        data.frame() %>%
        mutate(duration = duration[i], amt = amt_vec[i], interval = interval[i],
            group = "No degradation") %>%
        filter(time <= 24)  # & time != 0.0
}
```

5.2 Consider drug degradation

5.2.1 Stability parameters

- Read from stability studies, estimated via mixed-effects model

```
df_stability_param =
↪  read.csv("C:/Users/OneDrive/ADR/meropenem/GitHub/Mixed_effect_stability/stability_param_mixed_eff.c[illegible]
↪  %>%
    mutate(b0 = b0 * -1, b1 = b1 * -1, b2 = b2 * -1, Brand = "All") %>%
    dplyr::rename(Temp = temp, Conc = conc)
```

- Derive Beta parameters

```
b0 = df_stability_param$b0
b1 = df_stability_param$b1
b2 = df_stability_param$b2
Temp = df_stability_param$Temp
Conc = df_stability_param$Conc
Brand = rep("All", length(Temp))
```

5.2.2 Define model & parameters

```
# model
Ehmann2019_degra = RxODE({
  # @param Set outside RxODE function due to using vector as described above
  k = b0 + b1 + b2
```

```
  # @pre
  if(Clcr <= CLCR_INF){
    Cl = tvCL*(1 + CLCR_CL*(Clcr - 80.8))*exp(eta.IIV.Cl + eta.IOV.Cl)
  } else {
    Cl = CLCR_INF*(60/1000)*exp(eta.IIV.Cl + eta.IOV.Cl)
  }

  Vc = tvVc*(Wt/70)^WT_Vc*exp(eta.IIV.Vc)
  Q = tvQ
  Vp = tvVp*(1 + ALB_Vp*(Alb - 2.8))*exp(eta.IIV.Vp)

  # @dynamics
  d/dt(Depot) = -k*Depot

  d/dt(Central) = ifelse(
    (duration == 8) |
      # Strategy for EI
      (duration != 8 & (
        (time <= duration) |
          (interval*1 <= time & time <= (interval*1 + duration)) |
          (interval*2 <= time & time <= (interval*2 + duration)) |
          (interval*3 <= time & time <= (interval*3 + duration))
      )),
    # During infusion, drug from Depot to Central with rate = Depot/Center
    Depot/duration - Central*(Cl/Vc + Q/Vc) + Peri*Q/Vp,
    # When infusion ended, Drug just drop from Central
    -Central*(Cl/Vc + Q/Vc) + Peri*Q/Vp
    )

  d/dt(Peri) = Central*Q/Vc - Peri*Q/Vp

  # @derived
  Cp = Central/Vc
})
```

5.2.3 Perfrom simulation

Problems: Because we merge 2 course of drug disposition -> risk to perform stochastic simulation (differences in `sim.id` (`thetaMat`) and `id` (`OMEGA`))

Solution: - Step 1: Obtain PK parameters (`Cli`, `Vdi`, etc) from stochastic simulation - Step 2: Perform deterministic simulation based on above parameters

```
# number of patients to simulate
no.patients = 1000

# Define covariate
Clcr_vec = c(80.8)

list_covariate = foreach(i = 1:length(Clcr_vec), .packages = c("tidyverse")) %dopar% {
```

```
  data.frame(
    id = 1:no.patients,
    Wt = rep(70, no.patients),
    Clcr = Clcr_vec[i],
    Alb = 2.8
  )
}

# omega values
Omega = lotri(
  eta.IIV.Cl ~ log((27.1/100)^2 + 1),
  eta.IIV.Vc ~ log((31.5/100)^2 + 1),
  eta.IIV.Vp ~ log((16.9/100)^2 + 1),
  eta.IOV.Cl ~ log((12.5/100)^2 + 1)
)

registerDoParallel(6)
# Obtain simulated params
list_param_degra = foreach(
  i = 1:length(list_covariate), .packages = c("tidyverse", "RxODE"), .combine = "rbind"
) %dopar% {
  set.seed(1234)

  Ehmann2019_degra %>% # Only use the distribution from this model
    et(amt = 0, cmt = 1, dur = 0) %>%
    et(0) %>%
    et(id = 1:no.patients) %>%
    rxSolve(
      c(tvCL = 9.25, tvVc = 7.89, tvQ = 28.4, tvVp = 16.1,
        CLCR_CL = 0.00977, CLCR_INF = 154, WT_Vc = 0.945, ALB_Vp  = -0.202,
        b0 = df_stability_param$b0[1], b1 = df_stability_param$b1[1], b2 =
        ↪  df_stability_param$b2[1], # This is not necessary, just value for function to
        ↪  work, because the purpose is list of params generated from the distribution
        ↪  (omega matrix above)
        interval = 8, duration = 8),
      iCov = list_covariate[[i]],
      keep = c("Wt", "Alb", "Clcr"),
      omega = Omega,
      cores = 6
    ) %>%
    data.frame() %>%
    select(id, Cl, Vc, Q, Vp, Clcr)
}

list_param_degra
```

5.2.3.1 Step 1: Obtain simulated params

- Create dosage regimen dataframe

```
# Define dosage duration and interval
interval = c(8, 6, 3, 8)
duration = c(8, 6, 3, 3)
```

```
num_of_replace_bottle = 24/interval

total_dose = c(3)
foreach(i = 1:length(total_dose), .packages = "tidyverse", .combine = "rbind") %dopar%
    {
        data.frame(total_dose = total_dose[i], dose_each =
        ↪  total_dose[i]/num_of_replace_bottle *
            1000) %>%
            mutate(num_of_replace_bottle = total_dose/dose_each * 1000, duration = c(8,
                6, 3, 3), interval = 24/num_of_replace_bottle)
    } -> df_dose

df_dose
```

- Create parameter `dataframe`, which include tested duration/interval, tested Temp and tested doses

```
foreach(j = 1:length(Temp), .packages = c("tidyverse", "foreach"), .combine = "rbind")
↪  %dopar% {
  foreach(i = 1:nrow(df_dose), .packages = c("tidyverse"), .combine = "rbind") %dopar% {
    list_param_degra %>%
      mutate(
        duration = df_dose$duration[i],
        interval = df_dose$interval[i],
        amt = df_dose$dose_each[i],
        total_dose = df_dose$total_dose[i],

        Temp = Temp[j],
        Conc = Conc[j],
        Brand = Brand[j],
        b0 = b0[j],
        b1 = b1[j],
        b2 = b2[j]
      )
  }
} -> list_param_degra

list_param_degra %>% distinct(across(everything()))
```

5.2.3.2 Step 2: Define model and param for deterministic simulation

```
Ehmann2019_degra_deter = RxODE({
  # @param Set outside RxODE function due to using vector as described above
  # Time-varying covariates
  k = b0 + b1 + b2

  # theta_i are the PK params from the simulated above
  Cl = Cli
  Vc = Vci
  Q = Qi
```

```
  Vp = Vpi

  # @dynamics
  d/dt(Depot) = -k*Depot

  d/dt(Central) = ifelse(
    (duration == 8) |
      # Strategy for EI
      (duration != 8 & (
        (time <= duration) |
          # because 3 doses were used -> *3
          (interval*1 <= time & time <= (interval*1 + duration)) |
          (interval*2 <= time & time <= (interval*2 + duration)) |
          (interval*3 <= time & time <= (interval*3 + duration))
      )),
    # During infusion, drug from Depot to Central with rate = Depot/duration
    Depot/duration - Central*(Cl/Vc + Q/Vc) + Peri*Q/Vp,
    # When infusion ended, Drug just drop from Central
    -Central*(Cl/Vc + Q/Vc) + Peri*Q/Vp
    )


  d/dt(Peri) = Central*Q/Vc - Peri*Q/Vp

  # @derived
  Cp = Central/Vc
})
```

5.2.3.2.1 Define model first

- We need another model for Loading dose (see d/dt(Central) to the differences)

```
Ehmann2019_degra_for_LD = RxODE({
    # @param Set outside RxODE function due to using vector as described above
    # Time-varying covariates
    k = b0 + b1 + b2

    # theta_i are the PK params from the simulated above
    Cl = Cli
    Vc = Vci
    Q = Qi
    Vp = Vpi

    # @dynamics
    d/dt(Depot) = -k * Depot
    # duration from Depot to Central = 0.5
    d/dt(Central) = Depot/0.5 - Central * (Cl/Vc + Q/Vc) + Peri * Q/Vp
    d/dt(Peri) = Central * Q/Vc - Peri * Q/Vp

    # @derived
    Cp = Central/Vc
})
```

5.2.3.2.2 Simulation with LD applied for all dose

- Define function to simulation

```
degra_simu_func = function(df_param,
                           Ehmann2019_degra_for_LD,
                           Ehmann2019_degra_deter_CI) {
  foreach(
    t = 1:nrow(df_param), .packages = c("tidyverse", "RxODE"), .combine = "rbind"
  ) %dopar% {
    param_degra = c(Cli = df_param$Cl[t],
                    Vci = df_param$Vc[t],
                    Qi = df_param$Q[t],
                    Vpi = df_param$Vp[t],

                    b0 = df_param$b0[t],     # stability param
                    b1 = df_param$b1[t],
                    b2 = df_param$b2[t],     # don't include vector (also for loops) in
                    ↪  RxODE

                    interval = df_param$interval[t],
                    duration = df_param$duration[t])  # set duration for Depot to Central

    # Loading dose for CI (use traditional model to code this stage)
    df_LD = Ehmann2019_degra_for_LD %>%
      et(amt = 500, cmt = 1, dur = 0) %>%
      et(seq(0, 0.5, by = 0.1)) %>%
      rxSolve(
        param_degra
      ) %>%
      data.frame()

    # M. Dose 1
    df_MD1 =
      Ehmann2019_degra_deter_CI %>%
      et(time = 0,
         amt = df_param$amt[t], cmt = 1, dur = 0) %>%
      et(seq(0.1, df_param$interval[t], by = 0.1)) %>%
      rxSolve(
        inits = c(
          Central = df_LD[df_LD$time == 0.5, ]$Central[1],
          Peri = df_LD[df_LD$time == 0.5, ]$Peri[1]
        ),
        param_degra
      ) %>%
      data.frame()

    # M.Dose 2
    df_MD2 = Ehmann2019_degra_deter_CI %>%
      et(amt = df_param$amt[t], cmt = 1, dur = 0) %>%
      et(seq(0.1, df_param$interval[t], by = 0.1)) %>%
      rxSolve(
        inits = c(
          Central = df_MD1[df_MD1$time == df_param$interval[t], ]$Central[1],
```

```
      Peri = df_MD1[df_MD1$time == df_param$interval[t], ]$Peri[1]
    ),
    param_degra
  ) %>%
  data.frame() %>%
  mutate(time = time + df_param$interval[t])

# M.Dose 3
df_MD3 = Ehmann2019_degra_deter_CI %>%
  et(amt = df_param$amt[t], cmt = 1, dur = 0) %>%
  et(seq(0.1, df_param$interval[t], by = 0.1)) %>%
  rxSolve(
    inits = c(
      Central = df_MD2[df_MD2$time == df_param$interval[t]*2, ]$Central[1],
      Peri = df_MD2[df_MD2$time == df_param$interval[t]*2, ]$Peri[1]
    ),
    param_degra
  ) %>%
  data.frame() %>%
  mutate(time = time + df_param$interval[t]*2)

# M.Dose 4
df_MD4 = Ehmann2019_degra_deter_CI %>%
  et(amt = df_param$amt[t], cmt = 1, dur = 0) %>%
  et(seq(0.1, df_param$interval[t], by = 0.1)) %>%
  rxSolve(
    inits = c(
      Central = df_MD3[df_MD3$time == df_param$interval[t]*3, ]$Central[1],
      Peri = df_MD3[df_MD3$time == df_param$interval[t]*3, ]$Peri[1]
    ),
    param_degra
  ) %>%
  data.frame() %>%
  mutate(time = time + df_param$interval[t]*3)

# M.Dose 5
df_MD5 = Ehmann2019_degra_deter_CI %>%
  et(amt = df_param$amt[t], cmt = 1, dur = 0) %>%
  et(seq(0.1, df_param$interval[t], by = 0.1)) %>%
  rxSolve(
    inits = c(
      Central = df_MD4[df_MD4$time == df_param$interval[t]*4, ]$Central[1],
      Peri = df_MD4[df_MD4$time == df_param$interval[t]*4, ]$Peri[1]
    ),
    param_degra
  ) %>%
  data.frame() %>%
  mutate(time = time + df_param$interval[t]*4)

# M.Dose 6
df_MD6 = Ehmann2019_degra_deter_CI %>%
  et(amt = df_param$amt[t], cmt = 1, dur = 0) %>%
  et(seq(0.1, df_param$interval[t], by = 0.1)) %>%
```

```
    rxSolve(
      inits = c(
        Central = df_MD5[df_MD5$time == df_param$interval[t]*5, ]$Central[1],
        Peri = df_MD5[df_MD5$time == df_param$interval[t]*5, ]$Peri[1]
      ),
      param_degra
    ) %>%
    data.frame() %>%
    mutate(time = time + df_param$interval[t]*5)

  # M.Dose 7
  df_MD7 = Ehmann2019_degra_deter_CI %>%
    et(amt = df_param$amt[t], cmt = 1, dur = 0) %>%
    et(seq(0.1, df_param$interval[t], by = 0.1)) %>%
    rxSolve(
      inits = c(
        Central = df_MD6[df_MD6$time == df_param$interval[t]*6, ]$Central[1],
        Peri = df_MD6[df_MD6$time == df_param$interval[t]*6, ]$Peri[1]
      ),
      param_degra
    ) %>%
    data.frame() %>%
    mutate(time = time + df_param$interval[t]*6)

  # M.Dose 8
  df_MD8 = Ehmann2019_degra_deter_CI %>%
    et(amt = df_param$amt[t], cmt = 1, dur = 0) %>%
    et(seq(0.1, df_param$interval[t], by = 0.1)) %>%
    rxSolve(
      inits = c(
        Central = df_MD7[df_MD7$time == df_param$interval[t]*7, ]$Central[1],
        Peri = df_MD7[df_MD7$time == df_param$interval[t]*7, ]$Peri[1]
      ),
      param_degra
    ) %>%
    data.frame() %>%
    mutate(time = time + df_param$interval[t]*7)

  # Merge LD (for CI) with 3 MD
  df_total = rbind(
    df_LD,
    rbind(df_MD1, df_MD2, df_MD3, df_MD4,#) %>%
          df_MD5, df_MD6, df_MD7, df_MD8) %>%
      mutate(time = time + 0.5)
  ) %>%
    filter(time <= 24.0) # & time != 0.0

  # remove un-need df to save memory
  rm(df_LD, df_MD1, df_MD2, df_MD3, df_MD4, df_MD5, df_MD6, df_MD7, df_MD8)
  # merge final df to parameter df
  return(
    df_total = df_total %>%
      mutate(
```

```
        id = df_param$id[t],
        Temp = df_param$Temp[t],
        Conc = df_param$Conc[t],
        Brand = df_param$Brand[t],
        total_dose = df_param$total_dose[t],
        amt = df_param$amt[t],
        duration = df_param$duration[t],
        interval = df_param$interval[t],
        Clcr = df_param$Clcr[t]
      ) %>%
      relocate(id)
    )
  }
}
```

- Simulation

```
registerDoParallel(6)

# Increase memory
gc()
memory.limit(size = 56000)

# Perform simulation
degra_simu = degra_simu_func(
  df_param = list_param_degra, #%>% filter(duration == 3), # Can filter to simulate in
  ↪  each duration to save memory then merge latter
  Ehmann2019_degra_for_LD = Ehmann2019_degra_for_LD,
  Ehmann2019_degra_deter_CI = Ehmann2019_degra_deter  # because 1 regimen was EI
)
```

5.3 PTA calculation and visualisation

- PTA with degradation

```
MIC_vec = c(2, 4, 8, 16)

registerDoParallel(6)
foreach(i = 1:length(MIC_vec), .packages = "tidyverse", .combine = "rbind") %dopar% {
  degra_simu %>%
    filter(time <= 24.0 & time != 0.0) %>%
    mutate(
      MIC = MIC_vec[i],#MIC_vector[j],
      Cp_GT_MIC = ifelse(Cp > MIC, 1, 0),
      regimen = ifelse(duration == 3 & interval == 8, "EI 3 h q8h",
                       paste("CI", duration, "h", sep = " "))
    ) %>% #distinct(amt, interval, duration, regimen)
    relocate(Cp_GT_MIC, .after = Cp) %>%
    group_by(Temp, Conc, regimen, id) %>%
    mutate(N = length(id)) %>%
    summarise(
```

```
      perc = sum(Cp_GT_MIC)/N*100
      #.groups = c("Temp", "duration", "total_dose", "Clcr", "id")
    ) %>%
    distinct(perc, .keep_all = TRUE) %>%
    group_by(Temp, Conc, regimen) %>%
    mutate(
      targer_atain_98 = ifelse(perc >= 98, 1, 0),
      N_sim_pts = length(id)
    ) %>%
    summarise(
      PTA_98 = sum(targer_atain_98)/N_sim_pts*100
    ) %>%
    distinct(.keep_all = TRUE) %>%
    ungroup() %>%
    mutate(
      MIC = MIC_vec[i]
    ) %>%
    pivot_longer(c(PTA_98), names_to = "Target", values_to = "PTA")
} -> PTA_degra
```

- No-degra

```
MIC_vec = c(2, 4, 8, 16)

registerDoParallel(6)

foreach(i = 1:length(MIC_vec), .packages = "tidyverse", .combine = "rbind") %dopar% {
  no_degra_simu %>%
    filter(time <= 24.0 & time != 0.0) %>%
    mutate(
      MIC = MIC_vec[i],#MIC_vector[j],
      Cp_GT_MIC = ifelse(Cp > MIC, 1, 0),
      regimen = ifelse(duration == 3 & interval == 8, "EI 3 h q8h",
                       paste("CI", duration, "h", sep = " "))
    ) %>% #distinct(amt, interval, duration, regimen)
    relocate(Cp_GT_MIC, .after = Cp) %>%
    group_by(regimen, id) %>%
    mutate(N = length(id)) %>%
    summarise(
      perc = sum(Cp_GT_MIC)/N*100
      #.groups = c("Temp", "duration", "total_dose", "Clcr", "id")
    ) %>%
    distinct(perc, .keep_all = TRUE) %>%
    group_by(regimen) %>%
    mutate(
      targer_atain_98 = ifelse(perc >= 98, 1, 0),
      N_sim_pts = length(id)
    ) %>%
    summarise(
      PTA_98 = sum(targer_atain_98)/N_sim_pts*100
    ) %>%
    distinct(.keep_all = TRUE) %>%
    ungroup() %>%
```

```
    mutate(
      MIC = MIC_vec[i]
    ) %>%
    pivot_longer(c(PTA_98), names_to = "Target", values_to = "PTA")
} -> PTA_no_degra
```

- Merge no-deg and deg together

```
PTA_degra %>%
  mutate(
    # Create group variable
    group = paste("Degradation (", Temp, "-", Conc, ")", sep = ""),
    ## and then standardize it
    group = case_when(
      group == "Degradation (25-1)" ~ "Degradation (25 \u00B0C - 1 g/48mL)",
      group == "Degradation (25-2)" ~ "Degradation (25 \u00B0C - 2 g/48mL)",
      group == "Degradation (30-1)" ~ "Degradation (30 \u00B0C - 1 g/48mL)",
      group == "Degradation (30-2)" ~ "Degradation (30 \u00B0C - 2 g/48mL)",
      group == "Degradation (37-1)" ~ "Degradation (37 \u00B0C - 1 g/48mL)",
      group == "Degradation (37-2)" ~ "Degradation (37 \u00B0C - 2 g/48mL)"
    )
  ) %>%
  dplyr::select(-c(Temp, Conc)) %>%
  bind_rows(
    PTA_no_degra %>% mutate(group = "No degradation")
  ) %>%
  mutate(
    # regimen = ifelse(
    #   regimen == "EI 3 h q8h", "EI 3 h\nq8h", regimen
    # ),
    regimen = factor(regimen,
                     levels = c("EI 3 h q8h", "CI 3 h", "CI 6 h", "CI 8 h"))
  ) -> PTA_df

PTA_df
```

- Visualizaion, black and white

```
MIC_labs = c("MIC = 2 mg/L", "MIC = 4 mg/L", "MIC = 8 mg/L", "MIC = 16 mg/L")
names(MIC_labs) <- c("2" ,"4", "8", "16")

PTA_df %>%
  
  ↪  #write.csv("C:/Users/OneDrive/ADR/meropenem/graph_report/20221203_PTA_table_100_4MIC.csv")
  ggplot(aes(x = as.factor(regimen), y = PTA, color = group, group = group)) +
  geom_line(aes(color = group), linewidth = 0.68) +
  facet_grid(.~MIC,
             labeller = labeller(MIC = MIC_labs)) +
  geom_point(aes(shape = group, color = group), size = 2, alpha = 0.8) +
  geom_hline(yintercept = 90, linetype = "dashed") +
  theme_bw() +
```

```
  scale_y_continuous(breaks = seq(0, 100, by = 20)) +
  scale_color_grey() +
  labs(
    x = "",
    y = "PTA (%) for treatment day 1\nof 98%fT>MIC"
    #caption = "Total 3g/day q8h with LD of 500 mg over 30 min"
    #subtitle = "Brand A, 25 \u00B0C"
  ) +
  theme(
    axis.title = element_text(size = 20),
    axis.text = element_text(size = 15),
    plot.caption = element_text(size = 18),
    strip.text = element_text(size = 18),
    legend.title = element_blank(),
    legend.text = element_text(size = 15),
    legend.position = "bottom",
    legend.spacing.y = unit(0.28, 'cm'),
    panel.grid = element_line(colour = "#F3F3F3"),
    strip.background = element_blank()
  ) +
  guides(color = guide_legend(byrow = TRUE))
```

- Visualization, color

```
# Color code
scale_colour_Tien <- function(...){
      library(scales)
      discrete_scale("colour","Publication",manual_pal(values =
       ↪  c("#386cb0","#fdb462","#7fc97f","#a6cee3","#E78AC3","#6f6f6f","#ef3b2c","#662506","#ffff33")),
       ↪  ...)

}

# Label
MIC_labs = c("MIC = 2 mg/L", "MIC = 4 mg/L", "MIC = 8 mg/L", "MIC = 16 mg/L")
names(MIC_labs) <- c("2" ,"4", "8", "16")

# Visu
PTA_df %>%
  #write.csv("C:/Users/Tien
   ↪  Nguyen/OneDrive/ADR/meropenem/graph_report/20221203_PTA_table_100_2MIC.csv")
  ggplot(aes(x = as.factor(regimen), y = PTA, color = group, group = group)) +
  geom_line(aes(color = group), linewidth = 0.68) +
  facet_grid(.~MIC,
             labeller = labeller(MIC = MIC_labs)) +
  geom_point(aes(shape = group, color = group), size = 2, alpha = 0.8) +
  geom_hline(yintercept = 90, linetype = "dashed") +
  theme_bw() +
  scale_y_continuous(breaks = seq(0, 100, by = 20)) +
  #scale_color_grey() +
  scale_colour_Tien() +
  labs(
    x = "",
```

```
    y = "PTA (%) for treatment day 1\nof 98%fT>MIC"
    #caption = "Total 3g/day q8h with LD of 500 mg over 30 min"
    #subtitle = "Brand A, 25 \u00B0C"
  ) +
  theme(
    axis.title = element_text(size = 20),
    axis.text = element_text(size = 15),
    plot.caption = element_text(size = 18),
    strip.text = element_text(size = 18),
    legend.title = element_blank(),
    legend.text = element_text(size = 15),
    legend.position = "bottom",
    legend.spacing.y = unit(0.28, 'cm'),
    panel.grid = element_line(colour = "#F3F3F3"),
    strip.background = element_blank()
  ) +
  guides(color = guide_legend(byrow = TRUE)) -> PTA_CIvsEI_plot

# ggsave(
#   "C:/Users/OneDrive/ADR/meropenem/graph_report/20240824_PTA_CIvsEI.png",
↪   PTA_CIvsEI_plot,
#   width = 16.8, height = 5.8, dpi = 1000
# )
```

6 Simulation with changing Clcr

- Here we perform stochastic simulation with changing `Clcr` for 8h duration dose

6.1 No degradation simulation - stochastic simulation

6.1.1 Dosage regimen for no-degra (3g/day)

```
duration = 8
interval = 8
interval_minus_duration = interval - duration

# Define num of patients for stochastic simulation
no.patients = 1000

# use 6 cores
registerDoParallel(6)

# apply LD for all dose (just for no-degradation)
list_dosage_regimen_all_LD = et(amt = 500, cmt = 1, dur = 0.5) %>%  # 500 loading
  et(time = 0.5, amt = 1000, addl = 3, duration = duration, ii = 8, cmt = 1) %>%
  et(id = 1:no.patients)
```

6.1.2 Covariate

```
Clcr_vec = c(10, 30, 60, 90, 120, 150)

list_covariate = foreach(i = 1:length(Clcr_vec), .packages = c("tidyverse")) %dopar%
    {
        data.frame(id = 1:no.patients, Wt = rep(70, no.patients), Clcr = Clcr_vec[i],
            Alb = 2.8)
    }
```

6.1.3 Perform simulation

- Apply LD for all dose (take ~1min to done)

```
# use 6 cores
registerDoParallel(6)

set.seed(123)
rxSetSeed(123)
no_degra_simu_list = foreach(i = 1:length(list_covariate), .packages = c("tidyverse",
    "RxODE")) %dopar% {
    set.seed(98)
    Ehmann2019 %>%
        rxSolve(list_dosage_regimen_all_LD %>%
            et(seq(0, 24, by = 0.1)), iCov = list_covariate[[i]], keep = c("Wt",
            "Alb", "Clcr"), omega = Omega, cores = 6) %>%
        data.frame() %>%
        mutate(duration = duration, Clcr = Clcr_vec[i], group = "No degradation")
}
```

6.2 Consider drug degradation

6.2.1 Stability parameters

- Read from stability studies estimated and generated via mixed-effects approach

```
df_stability_param =
↪   read.csv("C:/Users/OneDrive/ADR/meropenem/GitHub/Mixed_effect_stability/stability_param_mixed_eff.c
↪   %>%
    mutate(b0 = b0 * -1, b1 = b1 * -1, b2 = b2 * -1, Brand = "All") %>%
    dplyr::rename(Temp = temp, Conc = conc)
```

- Derive Beta parameters

```
b0 = df_stability_param$b0
b1 = df_stability_param$b1
b2 = df_stability_param$b2
Temp = df_stability_param$Temp
Conc = df_stability_param$Conc
Brand = rep("All", length(Temp))
```

6.2.2 Define model & parameters

```
# model
Ehmann2019_degra = RxODE({
  # @param Set outside RxODE function due to using vector as described above
  k = b0 + b1 + b2

  # @pre
  if(Clcr <= CLCR_INF){
    Cl = tvCL*(1 + CLCR_CL*(Clcr - 80.8))*exp(eta.IIV.Cl + eta.IOV.Cl)
  } else {
    Cl = CLCR_INF*(60/1000)*exp(eta.IIV.Cl + eta.IOV.Cl)
  }

  Vc = tvVc*(Wt/70)^WT_Vc*exp(eta.IIV.Vc)
  Q = tvQ
  Vp = tvVp*(1 + ALB_Vp*(Alb - 2.8))*exp(eta.IIV.Vp)

  # @dynamics
  d/dt(Depot) = -k*Depot

  d/dt(Central) = ifelse(
    (duration == 8) |
      # Strategy for EI
      (duration != 8 & (
        (time <= duration) |
          (interval*1 <= time & time <= (interval*1 + duration)) |
          (interval*2 <= time & time <= (interval*2 + duration)) |
          (interval*3 <= time & time <= (interval*3 + duration))
      )),
    # During infusion, drug from Depot to Central with rate = Depot/Center
    Depot/duration - Central*(Cl/Vc + Q/Vc) + Peri*Q/Vp,
    # When infusion ended, Drug just drop from Central
    -Central*(Cl/Vc + Q/Vc) + Peri*Q/Vp
    )

  d/dt(Peri) = Central*Q/Vc - Peri*Q/Vp

  # @derived
  Cp = Central/Vc
})
```

6.2.3 Perfrom simulation

Problems: Because we merge 2 course of drug disposition -> risk to perform stochastic simulation (differences in `sim.id` (`thetaMat`) and `id` (`OMEGA`))

Solution: - Step 1: Obtain PK parameters (`Cli`, `Vdi`, etc) from stochastic simulation - Step 2: Perform deterministic simulation based on above parameters

6.2.4 Step 1: Obtain simulated params

```
# number of patients to simulate
no.patients = 1000

# Define covariates
Clcr_vec = c(10, 30, 60, 90, 120, 150)

list_covariate = foreach(i = 1:length(Clcr_vec), .packages = c("tidyverse")) %dopar% {
  data.frame(
    id = 1:no.patients,
    Wt = rep(70, no.patients),
    Clcr = Clcr_vec[i],
    Alb = 2.8
  )
}

# Define dosage duration and interval
interval = 8
duration = 8

# Obtain simulated params
list_param_degra = foreach(i = 1:length(list_covariate), .packages = c("tidyverse",
↪  "RxODE")) %dopar% {
  set.seed(1234)

  Ehmann2019_degra %>% # Only use the distribution from this model
    et(amt = 0, cmt = 1, dur = 0) %>%
    et(0) %>%
    et(id = 1:no.patients) %>%
    rxSolve(
      c(tvCL = 9.25, tvVc = 7.89, tvQ = 28.4, tvVp = 16.1,
        CLCR_CL = 0.00977, CLCR_INF = 154, WT_Vc = 0.945, ALB_Vp  = -0.202,
        b0 = b0[1], b1 = b1[1], b2 = b2[1], # This is not necessary, just value for
        ↪  function to work, because the purpose is list of params generated from the
        ↪  distribution (omega matrix above)
        interval = 8, duration = duration),
      iCov = list_covariate[[i]],
      keep = c("Wt", "Alb", "Clcr"),
      omega = Omega,
      cores = 6
    ) %>%
    data.frame() %>%
    select(id, Cl, Vc, Q, Vp)
}
```

6.2.5 Step 2: Define model and param for deterministic simulation

6.2.5.1 Define model first

- Degradation model for deterministic simulation

```
Ehmann2019_degra_deter = RxODE({
  # @param Set outside RxODE function due to using vector as described above
  # Time-varying covariates
  k = b0 + b1 + b2

  # theta_i are the PK params from the simulated above
  Cl = Cli
  Vc = Vci
  Q = Qi
  Vp = Vpi

  # @dynamics
  d/dt(Depot) = -k*Depot

  d/dt(Central) = ifelse(
    (duration == 8) |
      # Strategy for EI
      (duration != 8 & (
        (time <= duration) |
          # because 3 doses were used -> *3
          (interval*1 <= time & time <= (interval*1 + duration)) |
          (interval*2 <= time & time <= (interval*2 + duration)) |
          (interval*3 <= time & time <= (interval*3 + duration))
      )),
    # During infusion, drug from Depot to Central with rate = Depot/duration
    Depot/duration - Central*(Cl/Vc + Q/Vc) + Peri*Q/Vp,
    # When infusion ended, Drug just drop from Central
    -Central*(Cl/Vc + Q/Vc) + Peri*Q/Vp
    )


  d/dt(Peri) = Central*Q/Vc - Peri*Q/Vp

  # @derived
  Cp = Central/Vc
})
```

- We need another model for Loading dose (see d/dt(Central) to the differences)

```
Ehmann2019_degra_for_LD = RxODE({
    # @param Set outside RxODE function due to using vector as described above
    # Time-varying covariates
    k = b0 + b1 + b2

    # theta_i are the PK params from the simulated above
    Cl = Cli
    Vc = Vci
    Q = Qi
    Vp = Vpi

    # @dynamics
    d/dt(Depot) = -k * Depot
    # duration from Depot to Central = 0.5
```

```
    d/dt(Central) = Depot/0.5 - Central * (Cl/Vc + Q/Vc) + Peri * Q/Vp
    d/dt(Peri) = Central * Q/Vc - Peri * Q/Vp

    # @derived
    Cp = Central/Vc
})
```

```
# use 6 cores
registerDoParallel(6)

set.seed(1234)
rxSetSeed(1234)

degra_simu_list = foreach(j = 1:length(unique(b0)), # i.e., number of brand (7)
                          .packages = c("tidyverse", "RxODE", "foreach"), .combine =
                          ↪  "rbind") %dopar% {
  foreach(i = 1:length(list_param_degra),  # i.e., number of Clcr group (6)
          .packages = c("tidyverse", "RxODE", "foreach")) %dopar% {
    foreach(t = 1:nrow(list_param_degra[[i]]),  # i.e., number of patient/ech group
    ↪  (1000)
            .packages = c("tidyverse", "RxODE"), .combine = "rbind") %dopar% {
      param_degra = c(Cli = list_param_degra[[i]]$Cl[t],
                      Vci = list_param_degra[[i]]$Vc[t],
                      Qi = list_param_degra[[i]]$Q[t],
                      Vpi = list_param_degra[[i]]$Vp[t],

                      b0 = b0[j],     # stability param
                      b1 = b1[j],
                      b2 = b2[j],     # don't include vector (also for loops) in RxODE

                      interval = 8,
                      duration = duration)   # set duration for Depot to Central

      # Loading dose for CI (use traditional model to code this stage)
      df_LD = Ehmann2019_degra_for_LD %>%
        et(amt = 500, cmt = 1, dur = 0) %>%
        et(seq(0, 0.5, by = 0.1)) %>%
        rxSolve(
          param_degra
        ) %>%
        data.frame()

      # M. Dose 1
      df_MD1 =
        Ehmann2019_degra_deter %>%
        et(time = 0,
           amt = 1000, cmt = 1, dur = 0) %>%
        et(seq(0.1, 8, by = 0.1)) %>%
        rxSolve(
          inits = c(
```

```
      Central = df_LD[df_LD$time == 0.5, ]$Central[1],
      Peri = df_LD[df_LD$time == 0.5, ]$Peri[1]
    ),
    param_degra
  ) %>%
  data.frame()

# M.Dose 2
df_MD2 = Ehmann2019_degra_deter %>%
  et(amt = 1000, cmt = 1, dur = 0) %>%
  et(seq(0.1, 8, by = 0.1)) %>%
  rxSolve(
    inits = c(
      Central = df_MD1[df_MD1$time == interval, ]$Central[1],
      Peri = df_MD1[df_MD1$time == interval, ]$Peri[1]
    ),
    param_degra
  ) %>%
  data.frame() %>%
  mutate(time = time + interval)

# M.Dose 3
df_MD3 = Ehmann2019_degra_deter %>%
  et(amt = 1000, cmt = 1, dur = 0) %>%
  et(seq(0.1, 8, by = 0.1)) %>%
  rxSolve(
    inits = c(
      Central = df_MD2[df_MD2$time == interval*2, ]$Central[1],
      Peri = df_MD2[df_MD2$time == interval*2, ]$Peri[1]
    ),
    param_degra
  ) %>%
  data.frame() %>%
  mutate(time = time + interval*2)

# Merge LD (for CI) with 3 MD
df_total = rbind(
  df_LD,
  rbind(df_MD1, df_MD2, df_MD3) %>% mutate(time = time + 0.5)
)

# remove un-need df to save memory
rm(df_LD, df_MD1, df_MD2, df_MD3)

# merge final df to parameter df
df_total = left_join(
  df_total,
  list_param_degra[[i]],
  by = c("Cl", "Vc", "Q", "Vp")
) %>%
  select(id, everything()) %>%
  mutate(
    duration = duration,
```

```
          Clcr = Clcr_vec[i],
          group = paste("Degradation",
                        paste("(",
                              df_stability_param$Temp[j], "-",
                              df_stability_param$Conc[j],
                              ")", sep = ""),
                        sep = " ")
        )
      }
    }
}
```

6.2.5.2 Simulation with LD applied for all dose

6.3 PTA visualization for Clcr changing

6.3.1 Re-difine function

```
PTA_calcu_Clcr <- function(simulation_list, MIC_vector) {
    foreach(i = 1:length(simulation_list), .packages = c("tidyverse", "foreach"),
        .combine = "rbind") %dopar% {
        foreach(j = 1:length(MIC_vector), .packages = c("tidyverse"), .combine = "rbind")
        ↪  %dopar%
            {
                # j = length(MIC)
                simulation_list[[i]] %>%
                  relocate(group) %>%
                  filter(time <= 24 & time != 0) %>%
                  mutate(MIC = MIC_vector[j], Cp_GT_MIC = ifelse(Cp > MIC, 1, 0)) %>%
                  relocate(Cp_GT_MIC, .after = Cp) %>%
                  group_by(group, Clcr, duration, id) %>%
                  mutate(N = length(id)) %>%
                  summarise(perc = sum(Cp_GT_MIC)/N * 100) %>%
                  distinct(perc, .keep_all = TRUE) %>%
                  group_by(group, Clcr, duration) %>%
                  mutate(targer_atain_98 = ifelse(perc >= 98, 1, 0), targer_atain_40 =
                  ↪  ifelse(perc >=
                    40, 1, 0), N_sim_pts = length(id)) %>%
                  summarise(PTA_98 = sum(targer_atain_98)/N_sim_pts * 100, PTA_40 =
                  ↪  sum(targer_atain_40)/N_sim_pts *
                    100) %>%
                  distinct(.keep_all = TRUE) %>%
                  ungroup() %>%
                  mutate(MIC = MIC_vector[j]) %>%
                  pivot_longer(c(PTA_98, PTA_40), names_to = "Target", values_to = "PTA")
            }
    }
}
```

6.3.2 PTA computation

- For no degradation

```
PTA_calcu_Clcr(simulation_list = no_degra_simu_list, MIC_vector = c(4, 8, 16)) ->
    PTA_no_degra_df
```

- For degradation

```
PTA_calcu_Clcr(simulation_list = degra_simu_list, MIC_vector = c(4, 8, 16)) ->
    PTA_degra_df
```

- Merge degra and no degra together

```
PTA_no_degra_df %>%
    rbind(PTA_degra_df) %>%
    arrange(group, Target) %>%
    mutate(group = ifelse(group == "Degradation (25-1)", "Degradation (25 °C - 1
    ↪  g/48mL)",
        ifelse(group == "Degradation (25-2)", "Degradation (25 °C - 2 g/48mL)",
            ifelse(group == "Degradation (30-1)", "Degradation (30 °C - 1 g/48mL)",
                ifelse(group == "Degradation (30-2)", "Degradation (30 °C - 2 g/48mL)",
                  ifelse(group == "Degradation (37-1)", "Degradation (37 °C - 1 g/48mL)",
                    ifelse(group == "Degradation (37-2)", "Degradation (37 °C - 2
                    ↪  g/48mL)",
                      "No degradation"))))))) -> PTA_df
```

6.3.3 Visualization

Black and white

```
MIC_labs = c("MIC = 4 mg/L", "MIC = 8 mg/L", "MIC = 16 mg/L")
names(MIC_labs) <- c("4", "8", "16")

target_labs = c("40%fT>MIC", "100%fT>MIC")
names(target_labs) <- c("PTA_40", "PTA_98")

PTA_df %>%
  filter(MIC %in% c(4,8,16) & Target == "PTA_98") %>%
  filter(duration == 8) %>%
  #write.csv("C:/Users/Tien
  ↪  Nguyen/OneDrive/ADR/meropenem/graph_report/20221223_PTA_Clcr.csv")
  ggplot(aes(x = as.factor(Clcr), y = PTA, color = group, group = group)) +
  geom_line(aes(color = group), linewidth = 0.68) +
  facet_grid(.~MIC,
             labeller = labeller(MIC = MIC_labs)) +
  geom_point(aes(shape = group, color = group), size = 2, alpha = 0.8) +
  geom_hline(yintercept = 90, linetype = "dashed") +
  theme_bw() +
  scale_y_continuous(breaks = seq(0, 100, by = 20)) +
```

```
  scale_color_grey() +
  labs(
    x = "Clcr (mL/min)",
    y = "PTA (%) for treatment day 1\nof 98%fT>MIC"
    #caption = "Total 3g/day q8h with LD of 500 mg over 30 min"
    #subtitle = "Brand A, 25 \u00B0C"
  ) +
  theme(
    axis.title = element_text(size = 20),
    axis.text = element_text(size = 15),
    plot.caption = element_text(size = 18),
    strip.text = element_text(size = 18),
    legend.title = element_blank(),
    legend.text = element_text(size = 15),
    legend.position = "bottom",
    legend.spacing.y = unit(0.28, 'cm'),
    panel.grid = element_line(colour = "#F3F3F3"),
    strip.background = element_blank()
  ) +
  guides(color = guide_legend(byrow = TRUE))
```

Color

```
# Label
MIC_labs = c("MIC = 4 mg/L", "MIC = 8 mg/L", "MIC = 16 mg/L")
names(MIC_labs) <- c("4", "8", "16")

target_labs = c("40%fT>MIC", "100%fT>MIC")
names(target_labs) <- c("PTA_40", "PTA_98")

# Color code
scale_colour_Tien <- function(...){
      library(scales)
      discrete_scale("colour","Publication",manual_pal(values =
       ↪  c("#386cb0","#fdb462","#7fc97f","#a6cee3","#E78AC3","#6f6f6f","#ef3b2c","#662506","#ffff33")),
       ↪  ...)

}

# Read again the calculated data
read.csv("C:/Users/Tien
↪  Nguyen/OneDrive/ADR/meropenem/graph_report/20221223_PTA_Clcr_Copy.csv", row.names =
↪  1) -> PTA_df

PTA_df %>%
  mutate(group = gsub(replacement = "\u00B0C", pattern = "\xb0C", PTA_df$group)) ->
  ↪  PTA_df

PTA_df %>%
  ggplot(aes(x = as.factor(Clcr), y = PTA, color = group, group = group)) +
  geom_line(aes(color = group), linewidth = 0.68) +
  facet_grid(.~MIC,
             labeller = labeller(MIC = MIC_labs)) +
```

```
  geom_point(aes(shape = group, color = group), size = 2, alpha = 0.8) +
  geom_hline(yintercept = 90, linetype = "dashed") +
  theme_bw() +
  scale_y_continuous(breaks = seq(0, 100, by = 20)) +
  #scale_color_grey() +
  scale_colour_Tien() +
  labs(
    x = "Clcr (mL/min)",
    y = "PTA (%) for treatment day 1\nof 98%fT>MIC"
    #caption = "Total 3g/day q8h with LD of 500 mg over 30 min"
    #subtitle = "Brand A, 25 \u00B0C"
  ) +
  theme(
    axis.title = element_text(size = 20),
    axis.text = element_text(size = 15),
    plot.caption = element_text(size = 18),
    strip.text = element_text(size = 18),
    legend.title = element_blank(),
    legend.text = element_text(size = 15),
    legend.position = "bottom",
    legend.spacing.y = unit(0.28, 'cm'),
    panel.grid = element_line(colour = "#F3F3F3"),
    strip.background = element_blank()
  ) +
  guides(color = guide_legend(byrow = TRUE)) -> PTA_by_Clcr_plot

# ggsave(
#   "C:/Users/Tien Nguyen/OneDrive/ADR/meropenem/graph_report/20240824_PTA_Clcr.png",
↪   PTA_by_Clcr_plot,
#   width = 14.8, height = 5.8, dpi = 1000
# )
```

7 Simulation with changing Clcr & duration & dose

7.1 Consider drug degradation

7.1.1 Stability parameters

- Read from stability studies estimated and generated via mixed-effect approach

```
df_stability_param =
↪   read.csv("C:/Users/OneDrive/ADR/meropenem/GitHub/Mixed_effect_stability/stability_param_mixed_eff.c
↪   %>%
    mutate(b0 = b0 * -1, b1 = b1 * -1, b2 = b2 * -1, Brand = "All") %>%
    dplyr::rename(Temp = temp, Conc = conc)
```

- Derive Beta parameters

```
b0 = df_stability_param$b0
b1 = df_stability_param$b1
```

```
b2 = df_stability_param$b2
Temp = df_stability_param$Temp
Conc = df_stability_param$Conc
Brand = rep("All", length(Temp))
```

7.1.2 Define model & parameters

```
# model
Ehmann2019_degra = RxODE({
  # @param Set outside RxODE function due to using vector as described above
  k = b0 + b1 + b2

  # @pre
  if(Clcr <= CLCR_INF){
    Cl = tvCL*(1 + CLCR_CL*(Clcr - 80.8))*exp(eta.IIV.Cl + eta.IOV.Cl)
  } else {
    Cl = CLCR_INF*(60/1000)*exp(eta.IIV.Cl + eta.IOV.Cl)
  }

  Vc = tvVc*(Wt/70)^WT_Vc*exp(eta.IIV.Vc)
  Q = tvQ
  Vp = tvVp*(1 + ALB_Vp*(Alb - 2.8))*exp(eta.IIV.Vp)

  # @dynamics
  d/dt(Depot) = -k*Depot

  d/dt(Central) = ifelse(
    (duration == 8) |
      # Strategy for EI
      (duration != 8 & (
        (time <= duration) |
          (interval*1 <= time & time <= (interval*1 + duration)) |
          (interval*2 <= time & time <= (interval*2 + duration)) |
          (interval*3 <= time & time <= (interval*3 + duration))
      )),
    # During infusion, drug from Depot to Central with rate = Depot/Center
    Depot/duration - Central*(Cl/Vc + Q/Vc) + Peri*Q/Vp,
    # When infusion ended, Drug just drop from Central
    -Central*(Cl/Vc + Q/Vc) + Peri*Q/Vp
    )

  d/dt(Peri) = Central*Q/Vc - Peri*Q/Vp

  # @derived
  Cp = Central/Vc
})
```

7.1.3 Perfrom simulation

Problems: Because we merge 2 course of drug disposition -> risk to perform stochastic simulation (differences in `sim.id` (`thetaMat`) and `id` (`OMEGA`))

Solution: - Step 1: Obtain PK parameters (`Cli`, `Vdi`, etc) from stochastic simulation - Step 2: Perform deterministic simulation based on above parameters

7.1.4 Step 1: Obtain simulated params

```
# number of patients to simulate
no.patients = 10

# Define covariate
Clcr_vec = c(10, 30, 60, 90, 120, 150)

list_covariate = foreach(i = 1:length(Clcr_vec), .packages = c("tidyverse")) %dopar% {
  data.frame(
    id = 1:no.patients,
    Wt = rep(70, no.patients),
    Clcr = Clcr_vec[i],
    Alb = 2.8
  )
}

# omega values
Omega = lotri(
  eta.IIV.Cl ~ log((27.1/100)^2 + 1),
  eta.IIV.Vc ~ log((31.5/100)^2 + 1),
  eta.IIV.Vp ~ log((16.9/100)^2 + 1),
  eta.IOV.Cl ~ log((12.5/100)^2 + 1)
)

registerDoParallel(6)
# Obtain simulated params
list_param_degra = foreach(
  i = 1:length(list_covariate), .packages = c("tidyverse", "RxODE"), .combine = "rbind"
) %dopar% {
  set.seed(1234)

  Ehmann2019_degra %>% # Only use the distribution from this model
    et(amt = 0, cmt = 1, dur = 0) %>%
    et(0) %>%
    et(id = 1:no.patients) %>%
    rxSolve(
      c(tvCL = 9.25, tvVc = 7.89, tvQ = 28.4, tvVp = 16.1,
        CLCR_CL = 0.00977, CLCR_INF = 154, WT_Vc = 0.945, ALB_Vp  = -0.202,
        b0 = df_stability_param$b0[1], b1 = df_stability_param$b1[1], b2 =
        ↪  df_stability_param$b2[1],
        # This is not necessary, just value, because the purpose is list of params
        interval = 8, duration = 8),
      iCov = list_covariate[[i]],
      keep = c("Wt", "Alb", "Clcr"),
      omega = Omega,
      cores = 6
    ) %>%
    data.frame() %>%
```

```
    select(id, Cl, Vc, Q, Vp, Clcr)
}
```

- Create dosage regimen dataframe

```
# Define dosage duration and interval
interval = c(3, 4, 6, 8)  #, 12, 24)
num_of_replace_bottle = 24/interval

total_dose = c(3, 4.5, 6)
foreach(i = 1:length(total_dose), .packages = "tidyverse", .combine = "rbind") %dopar%
    {
        data.frame(total_dose = total_dose[i], dose_each =
        ↪  total_dose[i]/num_of_replace_bottle *
            1000) %>%
            mutate(num_of_replace_bottle = total_dose/dose_each * 1000, duration =
            ↪  24/num_of_replace_bottle,
                interval = 24/num_of_replace_bottle)
    } -> df_dose

df_dose
```

- Create parameter `dataframe`, which include tested `duration/interval`, tested `Temp` and tested `doses`

```
foreach(j = 1:length(Temp), .packages = c("tidyverse", "foreach"), .combine = "rbind")
↪  %dopar%
    {
        foreach(i = 1:nrow(df_dose), .packages = c("tidyverse"), .combine = "rbind")
        ↪  %dopar%
            {
                list_param_degra %>%
                  mutate(duration = df_dose$duration[i], interval = df_dose$interval[i],
                    amt = df_dose$dose_each[i], total_dose = df_dose$total_dose[i],
                    Temp = Temp[j])
            }
    } %>%
    left_join(df_stability_param, by = "Temp") -> list_param_degra

list_param_degra
```

7.1.5 Step 2: Define model and param for deterministic simulation

7.1.5.1 Define model first

- Re-define model because all regimens were CI

```
Ehmann2019_degra_deter_CI = RxODE({
    # @param Set outside RxODE function due to using vector as described above
    k = b0 + b1 + b2
```

```
    # theta_i are the PK params from the simulated above
    Cl = Cli
    Vc = Vci
    Q = Qi
    Vp = Vpi

    # @dynamics
    d/dt(Depot) = -k * Depot

    # Because all regimens were CI -> use for CI strategy
    d/dt(Central) = Depot/duration - Central * (Cl/Vc + Q/Vc) + Peri * Q/Vp

    d/dt(Peri) = Central * Q/Vc - Peri * Q/Vp

    # @derived
    Cp = Central/Vc
})
```

- We need another model for Loading dose (see d/dt(Central) to the differences)

```
Ehmann2019_degra_for_LD = RxODE({
    # @param Set outside RxODE function due to using vector as described above
    # Time-varying covariates
    k = b0 + b1 + b2

    # theta_i are the PK params from the simulated above
    Cl = Cli
    Vc = Vci
    Q = Qi
    Vp = Vpi

    # @dynamics
    d/dt(Depot) = -k * Depot
    # duration from Depot to Central = 0.5
    d/dt(Central) = Depot/0.5 - Central * (Cl/Vc + Q/Vc) + Peri * Q/Vp
    d/dt(Peri) = Central * Q/Vc - Peri * Q/Vp

    # @derived
    Cp = Central/Vc
})
```

7.1.5.2 Simulation with LD applied for all dose

- Define function to simulation

```
degra_simu_func = function(df_param,
                           Ehmann2019_degra_for_LD,
                           Ehmann2019_degra_deter_CI) {
  foreach(
    t = 1:nrow(df_param), .packages = c("tidyverse", "RxODE"), .combine = "rbind"
```

```
) %dopar% {
  param_degra = c(Cli = df_param$Cl[t],
                  Vci = df_param$Vc[t],
                  Qi = df_param$Q[t],
                  Vpi = df_param$Vp[t],

                  b0 = df_param$b0[t],    # stability param
                  b1 = df_param$b1[t],
                  b2 = df_param$b2[t],    # don't include vector (also for loops) in
                   ↪  RxODE

                  interval = df_param$interval[t],
                  duration = df_param$duration[t])  # set duration for Depot to Central

  # Loading dose for CI (use traditional model to code this stage)
  df_LD = Ehmann2019_degra_for_LD %>%
    et(amt = 500, cmt = 1, dur = 0) %>%
    et(seq(0, 0.5, by = 0.1)) %>%
    rxSolve(
      param_degra
    ) %>%
    data.frame()

  # M. Dose 1
  df_MD1 =
    Ehmann2019_degra_deter_CI %>%
    et(time = 0,
       amt = df_param$amt[t], cmt = 1, dur = 0) %>%
    et(seq(0.1, df_param$interval[t], by = 0.1)) %>%
    rxSolve(
      inits = c(
        Central = df_LD[df_LD$time == 0.5, ]$Central[1],
        Peri = df_LD[df_LD$time == 0.5, ]$Peri[1]
      ),
      param_degra
    ) %>%
    data.frame()

  # M.Dose 2
  df_MD2 = Ehmann2019_degra_deter_CI %>%
    et(amt = df_param$amt[t], cmt = 1, dur = 0) %>%
    et(seq(0.1, df_param$interval[t], by = 0.1)) %>%
    rxSolve(
      inits = c(
        Central = df_MD1[df_MD1$time == df_param$interval[t], ]$Central[1],
        Peri = df_MD1[df_MD1$time == df_param$interval[t], ]$Peri[1]
      ),
      param_degra
    ) %>%
    data.frame() %>%
    mutate(time = time + df_param$interval[t])

  # M.Dose 3
```

```
df_MD3 = Ehmann2019_degra_deter_CI %>%
  et(amt = df_param$amt[t], cmt = 1, dur = 0) %>%
  et(seq(0.1, df_param$interval[t], by = 0.1)) %>%
  rxSolve(
    inits = c(
      Central = df_MD2[df_MD2$time == df_param$interval[t]*2, ]$Central[1],
      Peri = df_MD2[df_MD2$time == df_param$interval[t]*2, ]$Peri[1]
    ),
    param_degra
  ) %>%
  data.frame() %>%
  mutate(time = time + df_param$interval[t]*2)

# M.Dose 4
df_MD4 = Ehmann2019_degra_deter_CI %>%
  et(amt = df_param$amt[t], cmt = 1, dur = 0) %>%
  et(seq(0.1, df_param$interval[t], by = 0.1)) %>%
  rxSolve(
    inits = c(
      Central = df_MD3[df_MD3$time == df_param$interval[t]*3, ]$Central[1],
      Peri = df_MD3[df_MD3$time == df_param$interval[t]*3, ]$Peri[1]
    ),
    param_degra
  ) %>%
  data.frame() %>%
  mutate(time = time + df_param$interval[t]*3)

# # M.Dose 5
# df_MD5 = Ehmann2019_degra_deter_CI %>%
#   et(amt = df_param$amt[t], cmt = 1, dur = 0) %>%
#   et(seq(0.1, df_param$interval[t], by = 0.1)) %>%
#   rxSolve(
#     inits = c(
#       Central = df_MD4[df_MD4$time == df_param$interval[t]*4, ]$Central[1],
#       Peri = df_MD4[df_MD4$time == df_param$interval[t]*4, ]$Peri[1]
#     ),
#     param_degra
#   ) %>%
#   data.frame() %>%
#   mutate(time = time + df_param$interval[t]*4)
#
# # M.Dose 6
# df_MD6 = Ehmann2019_degra_deter_CI %>%
#   et(amt = df_param$amt[t], cmt = 1, dur = 0) %>%
#   et(seq(0.1, df_param$interval[t], by = 0.1)) %>%
#   rxSolve(
#     inits = c(
#       Central = df_MD5[df_MD5$time == df_param$interval[t]*5, ]$Central[1],
#       Peri = df_MD5[df_MD5$time == df_param$interval[t]*5, ]$Peri[1]
#     ),
#     param_degra
#   ) %>%
#   data.frame() %>%
```

```r
#   mutate(time = time + df_param$interval[t]*5)
#
# # M.Dose 7
# df_MD7 = Ehmann2019_degra_deter_CI %>%
#   et(amt = df_param$amt[t], cmt = 1, dur = 0) %>%
#   et(seq(0.1, df_param$interval[t], by = 0.1)) %>%
#   rxSolve(
#     inits = c(
#       Central = df_MD6[df_MD6$time == df_param$interval[t]*6, ]$Central[1],
#       Peri = df_MD6[df_MD6$time == df_param$interval[t]*6, ]$Peri[1]
#     ),
#     param_degra
#   ) %>%
#   data.frame() %>%
#   mutate(time = time + df_param$interval[t]*6)
#
# # M.Dose 8
# df_MD8 = Ehmann2019_degra_deter_CI %>%
#   et(amt = df_param$amt[t], cmt = 1, dur = 0) %>%
#   et(seq(0.1, df_param$interval[t], by = 0.1)) %>%
#   rxSolve(
#     inits = c(
#       Central = df_MD7[df_MD7$time == df_param$interval[t]*7, ]$Central[1],
#       Peri = df_MD7[df_MD7$time == df_param$interval[t]*7, ]$Peri[1]
#     ),
#     param_degra
#   ) %>%
#   data.frame() %>%
#   mutate(time = time + df_param$interval[t]*7)

# Merge LD (for CI) with 3 MD
df_total = rbind(
  df_LD,
  rbind(df_MD1, df_MD2, df_MD3, df_MD4) %>%
        #df_MD5, df_MD6, df_MD7, df_MD8) %>%
    mutate(time = time + 0.5)
)

# remove un-need df to save memory
rm(df_LD, df_MD1, df_MD2, df_MD3, df_MD4)#, df_MD5, df_MD6, df_MD7, df_MD8)
# merge final df to parameter df
return(
  df_total = df_total %>%
    mutate(
      id = df_param$id[t],
      Temp = df_param$Temp[t],
      Conc = df_param$Conc[t],
      Brand = df_param$Brand[t],
      total_dose = df_param$total_dose[t],
      amt = df_param$amt[t],
      duration = df_param$duration[t],
      interval = df_param$interval[t],
      Clcr = df_param$Clcr[t]
```

```
        ) %>%
        relocate(id)
    )
  }
}
```

```
registerDoParallel(6)

# Increase memory
gc()
memory.limit(size = 56000)

# Perform simulation
degra_simu_list = degra_simu_func(
  df_param = list_param_degra, #%>% filter(duration == 3), #can simulate for each
  ↪  duration to save memory
  Ehmann2019_degra_for_LD = Ehmann2019_degra_for_LD,
  Ehmann2019_degra_deter_CI = Ehmann2019_degra_deter_CI
)
```

7.1.5.2.1 Simulate

7.2 PTA visualization

- PTA for MIC 8 and 16 mg/L

```
#MIC = 8
degra_simu_list %>%
  filter(time <= 24.0 & time != 0.0) %>%
  mutate(
    MIC = 8,#MIC_vector[j],
    Cp_GT_MIC = ifelse(Cp > MIC, 1, 0)
  ) %>%
  relocate(Cp_GT_MIC, .after = Cp) %>%
  group_by(Temp, duration, total_dose, Clcr, id) %>%
  mutate(N = length(id)) %>%
  summarise(
    perc = sum(Cp_GT_MIC)/N*100
    #.groups = c("Temp", "duration", "total_dose", "Clcr", "id")
  ) %>%
  distinct(perc, .keep_all = TRUE) %>%
  group_by(Temp, duration, total_dose, Clcr) %>%
  mutate(
    targer_atain_98 = ifelse(perc >= 98, 1, 0),
    targer_atain_40 = ifelse(perc >= 40, 1, 0),
    N_sim_pts = length(id)
  ) %>%
  summarise(
```

```
    PTA_98 = sum(targer_atain_98)/N_sim_pts*100,
    PTA_40 = sum(targer_atain_40)/N_sim_pts*100
  ) %>%
  distinct(.keep_all = TRUE) %>%
  ungroup() %>%
  mutate(
    MIC = 8
  ) %>%
  pivot_longer(c(PTA_98, PTA_40), names_to = "Target", values_to = "PTA") ->
  ↪  PTA_Clcr_dosage

#MIC = 16
degra_simu_list %>%
  filter(time <= 24.0 & time != 0.0) %>%
  mutate(
    MIC = 16,#MIC_vector[j],
    Cp_GT_MIC = ifelse(Cp > MIC, 1, 0)
  ) %>%
  relocate(Cp_GT_MIC, .after = Cp) %>%
  group_by(Temp, duration, total_dose, Clcr, id) %>%
  mutate(N = length(id)) %>%
  summarise(
    perc = sum(Cp_GT_MIC)/N*100
    #.groups = c("Temp", "duration", "total_dose", "Clcr", "id")
  ) %>%
  distinct(perc, .keep_all = TRUE) %>%
  group_by(Temp, duration, total_dose, Clcr) %>%
  mutate(
    targer_atain_98 = ifelse(perc >= 98, 1, 0),
    targer_atain_40 = ifelse(perc >= 40, 1, 0),
    N_sim_pts = length(id)
  ) %>%
  summarise(
    PTA_98 = sum(targer_atain_98)/N_sim_pts*100,
    PTA_40 = sum(targer_atain_40)/N_sim_pts*100
  ) %>%
  distinct(.keep_all = TRUE) %>%
  ungroup() %>%
  mutate(
    MIC = 16
  ) %>%
  pivot_longer(c(PTA_98, PTA_40), names_to = "Target", values_to = "PTA") ->
  ↪  PTA_Clcr_dosage_MIC16
```

- Plot

```
PTA_Clcr_dosage %>%
    mutate(MIC = 8) %>%
    bind_rows(PTA_Clcr_dosage_MIC16 %>%
        mutate(MIC = 16)) %>%
    mutate(group = cut(PTA, breaks = c(0, 10, 30, 50, 80, 90, 100))) -> PTA_df
```

```
library(ggh4x)
PTA_df %>%
    mutate(MIC = paste("MIC: ", MIC, " mg/L", sep = ""), MIC = factor(MIC, levels =
    ↪  c("MIC: 8 mg/L",
        "MIC: 16 mg/L")), total_dose = paste("Dose: ", total_dose, " g/day", sep = ""))
        ↪  %>%
    filter(Clcr != 10) %>%
    filter(Target == "PTA_98") %>%
    ggplot(aes(x = as.factor(Clcr), y = as.factor(duration), fill = group)) +
    ↪  geom_tile(alpha = 0.68) +
    # scale_fill_gradient(low = 'red', high = 'green') +
facet_nested(Temp ~ MIC + total_dose) + #facet_grid(Temp~total_dose) + facet_nested(Temp
facet_nested(Temp ~ MIC + total_dose) + #facet_grid(Temp~total_dose) + ~ MIC +
facet_nested(Temp ~ MIC + total_dose) + #facet_grid(Temp~total_dose) + total_dose)
facet_nested(Temp ~ MIC + total_dose) + #facet_grid(Temp~total_dose) + +
facet_nested(Temp ~ MIC + total_dose) + #facet_grid(Temp~total_dose) +
↪  #facet_grid(Temp~total_dose)
facet_nested(Temp ~ MIC + total_dose) + #facet_grid(Temp~total_dose) + +
geom_text(aes(label = PTA), size = 3) + scale_fill_manual(breaks = levels(PTA_df$group),
    values = c("red", "#FF1919", "#FF4747", "orange", "yellow", "green")) + theme_bw() +
    labs(x = "Clcr (mL/min)", y = "Period of Bottle Renewal (h)") + theme(axis.title =
    ↪  element_text(size = 16),
    axis.text = element_text(size = 13.2), plot.caption = element_text(size = 18),
    strip.text = element_text(size = 15.2), legend.title = element_blank(), legend.text =
    ↪  element_text(size = 15),
    legend.position = "none", panel.grid = element_line(colour = "#F3F3F3"),
    ↪  strip.background = element_blank())
```

8 Perfrom deterministic simulation

```
no.patients = 1

df_covariate = data.frame(id = 1:no.patients, Wt = rep(70, no.patients), Clcr = 80.8,
    Alb = 2.8)
```

8.1 Stability parameters

- Read from stability studies estimated and generated via mixed-effect approach

```
df_stability_param = read.csv("C:/Users/Tien
↪  Nguyen/OneDrive/ADR/meropenem/code/stability_param_mixed_eff.csv") %>%
    mutate(b0 = b0 * -1, b1 = b1 * -1, b2 = b2 * -1)
```

- Derive Beta parameters

```
b0 = df_stability_param$b0
b1 = df_stability_param$b1
```

```
b2 = df_stability_param$b2
Temp = df_stability_param$Temp
Conc = df_stability_param$Conc
Brand = rep("All", length(Temp))
```

- Run this block of code to perform deterministic simulation for all brands

```
# Re-read parameters
df_stability_param = read.csv(
  "C:/Users/Tien Nguyen/OneDrive/ADR/meropenem/code/stability_param_mixed_eff_EBE_each_brand.csv"
) %>%
  mutate(
    #Temp = as.factor(Temp),
    #Conc = as.factor(Conc),
    b0 = b0*-1, b1 = b1*-1, b2 = b2*-1
  )

b0 = df_stability_param$b0
b1 = df_stability_param$b1
b2 = df_stability_param$b2
Temp = df_stability_param$Temp
Conc = df_stability_param$Conc
Brand = df_stability_param$Brand
```

8.2 No degradaion

```
# Dosage Regimen
duration = c(8)
interval = 8

list_dosage_regimen_all_LD = foreach(i = 1:length(duration), .packages = c("tidyverse",
↪  "RxODE")) %dopar% {
  et(amt = 500, cmt = 1, dur = 0.5) %>%  # 500 loading
    et(time = 0.5, amt = 1000, addl = 10, duration = duration[i], ii = 8, cmt = 1) %>%
    et(id = 1:no.patients)
}

# Deterministic simulation
registerDoParallel(6)
foreach(i = 1:length(list_dosage_regimen_all_LD), .packages = c("tidyverse", "RxODE"),
        .combine = "rbind") %dopar% {
  Ehmann2019 %>%
    rxSolve(
      list_dosage_regimen_all_LD[[i]] %>% et(seq(0, 72, by = 0.1)),
      iCov = df_covariate,
      keep = c("Wt", "Alb", "Clcr"),
      omega = lotri(
        eta.IIV.Cl ~ 0, eta.IIV.Vc ~ 0,
        eta.IIV.Vp ~ 0, eta.IOV.Cl ~ 0
      ),
```

```
      cores = 6
    ) %>%
    data.frame() %>%
    mutate(
      duration = duration[i],
      group = "No degradation"
    )
} -> df_deter_no_degra
```

8.3 Degradation

8.3.1 Re-define model

- Because all regimens were CI

```
Ehmann2019_degra_deter_CI = RxODE({
    # @param Set outside RxODE function due to using vector as described above
    k = b0 + b1 + b2

    # theta_i are the PK params from the simulated above
    Cl = Cli
    Vc = Vci
    Q = Qi
    Vp = Vpi

    # @dynamics
    d/dt(Depot) = -k * Depot

    # Because all regimens were CI -> use for CI strategy
    d/dt(Central) = Depot/duration - Central * (Cl/Vc + Q/Vc) + Peri * Q/Vp

    d/dt(Peri) = Central * Q/Vc - Peri * Q/Vp

    # @derived
    Cp = Central/Vc
})
```

- We need another model for `Loading dose`

```
Ehmann2019_degra_for_LD = RxODE({
    # @param Set outside RxODE function due to using vector as described above
    # Time-varying covariates
    k = b0 + b1 + b2

    # theta_i are the PK params from the simulated above
    Cl = Cli
    Vc = Vci
    Q = Qi
    Vp = Vpi

    # @dynamics
```

```
    d/dt(Depot) = -k * Depot
    # duration from Depot to Central = 0.5
    d/dt(Central) = Depot/0.5 - Central * (Cl/Vc + Q/Vc) + Peri * Q/Vp
    d/dt(Peri) = Central * Q/Vc - Peri * Q/Vp

    # @derived
    Cp = Central/Vc
})
```

8.3.2 Simulate

- Generate parameters

```
# duration = c(24, 12, 8, 6, 3)
# interval = c(24, 12, 8, 6, 3)
# amount = c(3000, 1500, 1000, 750, 375)

duration = c(8, 6, 3)
interval = c(8, 6, 3)
amount = c(1000, 750, 375)

# Obtain simulated parameters first
Ehmann2019 %>% # Only use the distribution from this model
  et(amt = 0, cmt = 1, dur = 0) %>%
  et(0) %>%
  et(id = 1:no.patients) %>%
  rxSolve(
    # This is not necessary, just value, because the purpose is list of params
    c(tvCL = 9.25, tvVc = 7.89, tvQ = 28.4, tvVp = 16.1,
      CLCR_CL = 0.00977, CLCR_INF = 154, WT_Vc = 0.945, ALB_Vp  = -0.202,
      b0 = 0, b1 = 0, b2 = 0,
      interval = interval[1], duration = duration[1]),
    iCov = df_covariate,
    keep = c("Wt", "Alb", "Clcr"),
    omega = lotri(
      eta.IIV.Cl ~ 0, eta.IIV.Vc ~ 0,
      eta.IIV.Vp ~ 0, eta.IOV.Cl ~ 0
    ),
    cores = 6
  ) %>%
  data.frame() -> df_param_degra;

df_param_degra
```

- Simulate

```
gc()
# Then perform deterministic simulation
registerDoParallel(6)

df_deter_degra = foreach(j = 1:nrow(df_stability_param),
```

```
        .packages = c("tidyverse", "RxODE", "foreach"), .combine = "rbind") %dopar% {
foreach(i = 1:length(duration), .packages = c("tidyverse", "RxODE", "foreach")) %dopar%
↪  {
  foreach(t = 1:nrow(df_param_degra), .packages = c("tidyverse", "RxODE"), .combine =
  ↪  "rbind") %dopar% {
    param_degra = c(Cli = df_param_degra$Cl[t],
                    Vci = df_param_degra$Vc[t],
                    Qi = df_param_degra$Q[t],
                    Vpi = df_param_degra$Vp[t],

                    b0 = b0[j],    # stability param
                    b1 = b1[j],
                    b2 = b2[j],    # don't include vector (also for loops) in RxODE

                    duration = duration[i])  # set duration for Depot to Central

    # Loading dose for CI (use traditional model to code this stage)
    df_LD = Ehmann2019_degra_for_LD %>%
      et(amt = 500, cmt = 1, dur = 0) %>%
      et(seq(0, 0.5, by = 0.1)) %>%
      rxSolve(
        param_degra
      ) %>%
      data.frame()

    # M. Dose 1
    df_MD1 =
      Ehmann2019_degra_deter_CI %>%
      et(time = 0,
         amt = amount[i], cmt = 1, dur = 0) %>%
      et(seq(0.1, interval[i], by = 0.1)) %>%
      rxSolve(
        inits = c(
          Central = df_LD[df_LD$time == 0.5, ]$Central[1],
          Peri = df_LD[df_LD$time == 0.5, ]$Peri[1]
        ),
        param_degra
      ) %>%
      data.frame()

    # M.Dose 2
    df_MD2 = Ehmann2019_degra_deter_CI %>%
      et(amt = amount[i], cmt = 1, dur = 0) %>%
      et(seq(0.1, interval[i], by = 0.1)) %>%
      rxSolve(
        inits = c(
          Central = df_MD1[df_MD1$time == interval[i], ]$Central[1],
          Peri = df_MD1[df_MD1$time == interval[i], ]$Peri[1]
        ),
        param_degra
      ) %>%
      data.frame() %>%
      mutate(time = time + interval[i])
```

```
# M.Dose 3
df_MD3 = Ehmann2019_degra_deter_CI %>%
  et(amt = amount[i], cmt = 1, dur = 0) %>%
  et(seq(0.1, interval[i], by = 0.1)) %>%
  rxSolve(
    inits = c(
      Central = df_MD2[df_MD2$time == interval[i]*2, ]$Central[1],
      Peri = df_MD2[df_MD2$time == interval[i]*2, ]$Peri[1]
    ),
    param_degra
  ) %>%
  data.frame() %>%
  mutate(time = time + interval[i]*2)

# M.Dose 4
df_MD4 = Ehmann2019_degra_deter_CI %>%
  et(amt = amount[i], cmt = 1, dur = 0) %>%
  et(seq(0.1, interval[i], by = 0.1)) %>%
  rxSolve(
    inits = c(
      Central = df_MD3[df_MD3$time == interval[i]*3, ]$Central[1],
      Peri = df_MD3[df_MD3$time == interval[i]*3, ]$Peri[1]
    ),
    param_degra
  ) %>%
  data.frame() %>%
  mutate(time = time + interval[i]*3)

# M.Dose 5
df_MD5 = Ehmann2019_degra_deter_CI %>%
  et(amt = amount[i], cmt = 1, dur = 0) %>%
  et(seq(0.1, interval[i], by = 0.1)) %>%
  rxSolve(
    inits = c(
      Central = df_MD4[df_MD4$time == interval[i]*4, ]$Central[1],
      Peri = df_MD4[df_MD4$time == interval[i]*4, ]$Peri[1]
    ),
    param_degra
  ) %>%
  data.frame() %>%
  mutate(time = time + interval[i]*4)

# M.Dose 6
df_MD6 = Ehmann2019_degra_deter_CI %>%
  et(amt = amount[i], cmt = 1, dur = 0) %>%
  et(seq(0.1, interval[i], by = 0.1)) %>%
  rxSolve(
    inits = c(
      Central = df_MD5[df_MD5$time == interval[i]*5, ]$Central[1],
      Peri = df_MD5[df_MD5$time == interval[i]*5, ]$Peri[1]
    ),
    param_degra
```

```
    ) %>%
    data.frame() %>%
    mutate(time = time + interval[i]*5)

  # M.Dose 7
  df_MD7 = Ehmann2019_degra_deter_CI %>%
    et(amt = amount[i], cmt = 1, dur = 0) %>%
    et(seq(0.1, interval[i], by = 0.1)) %>%
    rxSolve(
      inits = c(
        Central = df_MD6[df_MD6$time == interval[i]*6, ]$Central[1],
        Peri = df_MD6[df_MD6$time == interval[i]*6, ]$Peri[1]
      ),
      param_degra
    ) %>%
    data.frame() %>%
    mutate(time = time + interval[i]*6)

  # M.Dose 8
  df_MD8 = Ehmann2019_degra_deter_CI %>%
    et(amt = amount[i], cmt = 1, dur = 0) %>%
    et(seq(0.1, interval[i], by = 0.1)) %>%
    rxSolve(
      inits = c(
        Central = df_MD7[df_MD7$time == interval[i]*7, ]$Central[1],
        Peri = df_MD7[df_MD7$time == interval[i]*7, ]$Peri[1]
      ),
      param_degra
    ) %>%
    data.frame() %>%
    mutate(time = time + interval[i]*7)

  # Merge LD (for CI) with 3 MD
  df_total = rbind(
    df_LD,
    rbind(df_MD1, df_MD2, df_MD3, df_MD4,
          df_MD5, df_MD6, df_MD7, df_MD8) %>% mutate(time = time + 0.5)
  )

  # remove un-need df to save memory
  rm(df_LD, df_MD1, df_MD2, df_MD3, df_MD4, df_MD5, df_MD6, df_MD7, df_MD8)

  # merge final df to parameter df
  df_total = left_join(
    df_total,
    df_param_degra %>% select(Cl, Vc, Q, Vp, Wt, Clcr, Alb),
    by = c("Cl", "Vc", "Q", "Vp")
  ) %>%
    #select(id, everything()) %>%
    mutate(
      duration = duration[i],
      Temp = Temp[j],
      Conc = Conc[j],
```

```
          Brand = Brand[j]
          # group = paste("Degradation",
          #               paste("(", df_stability_param$Temp[j], "-",
          #                     df_stability_param$Conc[j],
          #                     ")", sep = ""),
          #               sep = " ")
        )
    }
  }
}
```

8.4 Visualization

8.4.1 Prepare data

```
do.call("rbind", df_deter_degra) %>%
    left_join(df_stability_param) -> df_deter_degra

df_deter_degra %>%
    mutate(duration = paste(duration, "h", sep = " "), Conc = ifelse(Conc == "1g/48mL",
        "1 g/48mL", "2 g/48mL")) -> df_deter_degra
```

8.4.2 Figure 3A (time-course)

```
df_deter_degra %>%
  filter(time <= 24.5) %>%
  #filter(Brand == "Q") %>% # & duration %in% c(6)) %>% #view() #%>%
  ggplot(aes(x = time, y = Cp)) +
  geom_line(aes(color = duration), linewidth = 0.8) +
  facet_grid(Conc ~ Temp) +
  theme_bw() +
  scale_color_grey(start = 0.8, end = 0.2) +
  labs(
    x = "Time (h)",
    y = "Concentration (mg/L)"
    #caption = "3g/day continuous infusion with LD of 500 mg over 30 min"
    #subtitle = "Brand A, 25 \u00B0C"
  ) +
  theme(
    axis.title = element_text(size = 18),
    axis.text = element_text(size = 15),
    plot.caption = element_text(size = 15),
    strip.text = element_text(size = 15),
    legend.title = element_text(size = 15),
    legend.text = element_text(size = 15),
    legend.position = "bottom",
    panel.grid = element_line(colour = "#F3F3F3"),
    strip.background = element_blank()
  ) +
```

```
  guides(
    color = guide_legend(title = "Degradation with duration (CI)", order = 2),
    linetype = guide_legend(title = "", order = 1)
  ) +
  geom_line(
    data = df_deter_no_degra %>% filter(time <= 24.5),
    aes(x = time, y = Cp, linetype = group),
    #linetype = "dashed",
    color = "red",
    size = 0.8,
    alpha = 0.46
  ) +
  scale_x_continuous(breaks = seq(0, 24, by = 4))
```

8.4.3 Figure 3B Cmin/Cno-deg

```
# Calculate Css_avg
Css_avg = df_deter_no_degra %>% filter(time <= 24.5 & time >= 12) %>% summarise(Css_avg =
↪  min(Cp)) %>% .$Css_avg

#
df_deter_degra %>%
  # mutate(
  #   duration = ifelse(duration == "3 h", "3 h (CI)",
  #                     ifelse(duration == "6 h", "6 h (CI)", "8h (CI)"))
  # ) %>%
  filter(time <= 24.5 & time >= 12) %>%
  group_by(Brand, Temp, Conc, duration) %>%
  summarise(
    Cmin = min(Cp)
  ) %>% #ungroup() %>% group_by(Brand) %>% summarise(min(Cmin))
  mutate(
    Css_avg = Css_avg,
    perc = Cmin/Css_avg*100
  ) %>%
  rename(Temperature = Temp) %>%
  ggplot(
    aes(x = as.factor(duration), y = perc)
  ) +
  geom_point(aes(color = Brand, shape = Temperature), size = 3.2, alpha = 0.48) +
  facet_wrap(~Conc, nrow = 2) +
  theme_bw() +
  theme(
    axis.title = element_text(size = 20),
    axis.text = element_text(size = 15),
    plot.caption = element_text(size = 18),
    strip.text = element_text(size = 18),
    legend.text = element_text(size = 18),
    legend.title = element_text(size = 19),
    legend.position = "right",
    panel.grid = element_line(colour = "#F3F3F3"),
```

```
  strip.background = element_blank()
) +
guides(color = guide_legend(ncol = 1)) +
labs(
  x = "Duration (continuous infusion)",
  y = expression(""*C["min"]/C["no-deg"]*" (%)")#"Ratio of Ctrough (%)"
) +
scale_y_continuous(limits = c(80, 100))
```
