# Supplementary material for: Optimization of meropenem continuous infusion based on Monte Carlo simulation integrating with degradation study
Source: PLoS One. 2024 Dec 23;19(12):e0313764. doi: 10.1371/journal.pone.0313764 (PMC11666027; doi:10.1371/journal.pone.0313764)
Supplement: S1 Table — (DOCX) [file pone.0313764.s005.docx]

**S1 Table. Estimated parameters for the model 4 in the stability study.**

|  | **Estimate** | **SE** | **95 % Confidence Interval** | **p-value** | |
| --- | --- | --- | --- | --- | --- |
| **Fixed effects** | | | | | |
| Intercept (µ) | –0.0003 | 0.0013 | –0.0024 – 0.0030 | 0.854 | |
| Time (β_0_) | 0.0086 | 0.0009 | 0.0068 – 0.0103 | <0.001 | |
| Temperature (β_1_) |  |  |  |  | |
| 25 °C | Reference |  |  |  | |
| 30 °C | 0.0042 | 0.0007 | 0.0028 – 0.0055 | <0.001 | |
| 37 °C | 0.0181 | 0.0007 | 0.0168 – 0.0194 | <0.001 | |
| Infusion concentration (β_2_) |  |  |  |  | |
| 1 g/48mL | Reference |  |  |  | |
| 2 g/48mL | 0.0040 | 0.0005 | 0.0029 – 0.0051 | <0.001 | |
| Brand (β_3_) |  |  |  |  | |
| Brand Q | Reference |  |  |  | |
| Brand A | 0.0005 | 0.0010 | -0.0025 – 0.0015 | 0.619 | |
| Brand E | -0.0010 | 0.0010 | -0.0011 – 0.0030 | 0.337 | |
| Brand I | 0.0003 | 0.0010 | -0.0023 – 0.0018 | 0.801 | |
| Brand R | -0.0012 | 0.0010 | -0.0009 – 0.0032 | 0.247 | |
| Brand V | -0.0019 | 0.0010 | -0.0002 – 0.0039 | 0.075 | |
| Brand X | 0.0010 | 0.0010 | -0.0031 – 0.0010 | 0.332 | |
| **Random effects** | | | | | |
| ω_1_ | 0.0064 |  | 0.0040 – 0.0090 |  | |
| ω_2_ | 0.0012 |  | 0.0003 – 0.0014 |  | |
| σ | 0.0101 |  | 0.0092 – 0.0108 |  | |
| SE: Standard Error | | | | |  |
